# The NUTRIENT Trial (NUTRitional Intervention among myEloproliferative Neoplasms): Results from a Randomized Phase I Pilot Study for Feasibility and Adherence

**DOI:** 10.1158/2767-9764.CRC-23-0380

**Published:** 2024-03-05

**Authors:** Laura F. Mendez Luque, Julio Avelar-Barragan, Hellen Nguyen, Jenny Nguyen, Eli M. Soyfer, Jiarui Liu, Jane H. Chen, Nitya Mehrotra, Xin Huang, Heidi E. Kosiorek, Amylou Dueck, Alexander Himstead, Elena Heide, Melinda Lem, Kenza El Alaoui, Eduard Mas, Robyn M. Scherber, Ruben A. Mesa, Katrine L. Whiteson, Andrew Odegaard, Angela G. Fleischman

**Affiliations:** 1 University of California, Irvine School of Medicine, Irvine, California.; 2 Teaching and Research Department, Institute of Public Health Services of the State of Baja California, Baja California, Mexico.; 3 Department of Molecular Biology and Biochemistry, University of California Irvine, Irvine, California.; 4 Department of Quantitative Health Sciences, Mayo Clinic Arizona, Scottsdale, Arizona.; 5 Mays Cancer Center, UT Health San Antonio, San Antonio, Texas.; 6 Atrium Health, Levine Cancer Institute, Charlotte, North Carolina.

## Abstract

**Purpose:**

Chronic inflammation is integral to myeloproliferative neoplasm (MPN) pathogenesis. JAK inhibitors reduce cytokine levels, but not without significant side effects. Nutrition is a low-risk approach to reduce inflammation and ameliorate symptoms in MPN. We performed a randomized, parallel-arm study to determine the feasibility of an education-focused Mediterranean diet intervention among patients with MPN.

**Experimental Design:**

We randomly assigned patients with MPN to either a Mediterranean diet or standard U.S. Dietary Guidelines for Americans (USDA). Groups received equal but separate education with registered dietician counseling and written dietary resources. Patients were prospectively followed for feasibility, adherence, and symptom burden assessments. Biological samples were collected at four timepoints during the 15-week study to explore changes in inflammatory biomarkers and gut microbiome.

**Results:**

The Mediterranean diet was as easy to follow for patients with MPN as the standard USDA diet. Approximately 80% of the patients in the Mediterranean diet group achieved a Mediterranean Diet Adherence Score of ≥8 throughout the entire active intervention period, whereas less than 50% of the USDA group achieved a score of ≥8 at any timepoint. Improvement in symptom burden was observed in both diet groups. No significant changes were observed in inflammatory cytokines. The diversity and composition of the gut microbiome remained stable throughout the duration of the intervention.

**Conclusions:**

With dietician counseling and written education, patients with MPN can adhere to a Mediterranean eating pattern. Diet interventions may be further developed as a component of MPN care, and potentially incorporated into the management of other hematologic conditions.

**Significance:**

Diet is a central tenant of management of chronic conditions characterized by subclinical inflammation, such as cardiovascular disease, but has not entered the treatment algorithm for clonal hematologic disorders. Here, we establish that a Mediterranean diet intervention is feasible in the MPN patient population and can improve symptom burden. These findings warrant large dietary interventions in patients with hematologic disorders to test the impact of diet on clinical outcomes.

## Introduction

Myeloproliferative neoplasms (MPN), including polycythemia vera (PV), essential thrombocythemia (ET), and primary myelofibrosis (PMF), are hematologic malignancies characterized by the clonal outgrowth of hematopoietic cells with a somatically acquired mutation most commonly in *JAK2* (*JAK2^V617F^*; refs. 1–5). The clinical consequences of MPN include thrombosis, transformation to acute leukemia, abnormal blood counts, and a significant symptom burden. MPN is a highly inflammatory disease, with increased plasma cytokines as a hallmark feature of the disease.

Chronic inflammation is pervasive in MPN, contributing to symptomatology (6), blood count abnormalities (7), and disease progression (8, 9). JAK inhibitors reduce inflammation (10, 11), resulting in amelioration of symptoms and improvement in quality of life (12, 13). However, JAK inhibitors are not without risks including immunosuppression, weight gain (14), and skin cancers (15), are extremely costly, and are not indicated for all patients with MPN. Recently developed National Comprehensive Cancer Network guidelines for MPN address the importance of symptom burden, and recommend intervention to reduce symptom burden regardless of prognosis scoring category (16). However, many patients with MPN do not meet criteria for a cytoreductive agent. Therefore, many patients with MPN are maintained without intervention that adequately address symptoms nor impact disease progression. Lifestyle modifications to reduce inflammation, such as diet, could have significant short-term as well as long-term positive impacts on the disease. In the short term, adopting a healthful diet rich in anti-inflammatory foods may serve to improve symptom burden among patients with MPN. In the long term, minimizing inflammation through diet may potentially delay or prevent disease progression.

The Mediterranean diet, characterized by increased consumption of extra virgin olive oil (EVOO), nuts, legumes, vegetables, fruits, fish, and whole grain products, has proven to be beneficial in diseases where chronic subclinical inflammation plays a key role (17). For example, the PREDIMED (Prevención con Dieta Mediterránea) study demonstrated that a Mediterranean diet supplemented with EVOO or nuts reduced the incidence of major cardiovascular events (18). The Mediterranean diet's anti-inflammatory properties are attributed to its richness in phenolic compounds and nutrient density (19).

Data are emerging that dietary and microbiome factors may be beneficial in clonal hematologic disorders (20, 21). In a cohort of patients with multiple myeloma on lenalidomide maintenance consumption of dietary flavonoids correlated with stool butyrate concentration, and higher stool butyrate concentration was associated with sustained minimal residual disease negativity (21). An ongoing dietary intervention study (NUTRIVENTION) of a whole food plant-based diet in patients with monoclonal gammopathy and smoldering multiple myeloma will evaluate the impact of diet in these precursor conditions (22).

Nutritional control of inflammation represents a unique low-risk therapeutic approach to alleviate the symptom burden of patients with MPN and to also possibly blunt disease progression. Here, we investigated the feasibility of employing an education-focused Mediterranean diet intervention in an MPN patient cohort. Our main goal was to establish that patients with MPN are willing and able to initiate dietary education as a potential symptom burden management. Furthermore, we also collected preliminary efficacy and mechanistic data on symptom burden, inflammatory cytokines, and the gut microbiome.

## Materials and Methods

### Study Information

The NUTRIENT (NUTRitional Intervention among myEloproliferative Neoplasms) study was a single center interventional pilot study of an educational dietary intervention among patients with MPN performed at University of California, Irvine (Irvine, CA) from October 2018 through December 2019. The protocol was approved by the Institutional Review Board of University of California Irvine and registered on clinicaltrials.gov (NCT03907436). The study was conducted in accordance with the U.S. Common Rule.

### Patient Recruitment and Demographics

We recruited individuals at UC Irvine (UCI) Health clinics who were 18 years of age or older and who had been previously diagnosed with a Philadelphia chromosome negative MPN including ET, PV, or myelofibrosis (MF, includes primary myelofibrosis as well as post-ET or post-PV myelofibrosis). All participants provided written informed consent. Any type of MPN directed therapy was allowed. A complete list of inclusion and exclusion criteria is provided in Supplementary Table S1. Thirty-one participants were randomized to either a Mediterranean Diet (MED) or U.S. Dietary Guidelines for Americans (USDA) arm, two withdrew due to family illness and one was lost to follow-up. Demographics of the 28 patients who completed the study are shown in Table 1. The representativeness of study participants is shown in Supplementary Table S2.

**TABLE 1:** Demographics of study cohort

	USDA (*n* = 13)	Mediterranean Diet (*n* = 15)	Total Cohort (*n* = 28)
Female *n* (%)	10 (76%)	10 (67%)	20 (71%)
Age mean (range)	58 (21–77)	57 (25–71)	58 (21–77)
Disease *n* (%)			
PV	6 (46%)	8 (53%)	14 (50%)
ET	3 (23%)	3 (20%)	6 (21%)
MF	4 (31%)	4 (27%)	8 (29%)
Race			
Asian	2 (15%)	1 (7%)	3 (11%)
White	11 (85%)	14 (93%)	25 (89%)
Ethnicity			
Hispanic	1 (8%)	0	1 (3%)
Mutation *n* (%)			
JAK2	12 (92%)	13 (87%)	15 (89%)
CALR	0	2 (13%)	2 (7%)
MPL	1 (8%)	0	1 (4%)
Treatment *n* (%)			
Hydroxyurea	4 (31%)	5 (34%)	9 (32%)
Ruxolitinib	1 (8%)	2 (13%)	3 (11%)
Interferon	2 (15%)	3 (20%)	5 (18%)
Other	6 (46%)	5 (33%)	11 (39%)
MPN-SAF median at baseline (interquartile range, IQR)	15.5 (17)	10.5 (12)	11.5 (13.5)
Mediterranean Diet Adherence Score median at baseline (IQR)	6.0 (2.5)	6.0 (4.5)	6.0 (3.0)

### Randomization

No stratification factors were used to randomize participants to each arm. At the initiation of the study, 20 sealed opaque envelopes were placed in a box, 15 with a paper slip labeled MED and 15 with a paper slip labeled USDA. Participants were randomized one day prior to their scheduled first dietician visit. At randomization, a blindfolded study staff reached into the box, picked an envelope, wrote the subject no. on the envelope, then opened the envelope to reveal MED or USDA. One replacement envelope was placed in the box to replace one participant who withdrew early in the study.

### Endpoints

The central study objective was to assess whether patients with MPN can adopt a Mediterranean eating pattern with dietician counseling and educational materials. We had a combined primary endpoint of both feasibility of and adherence to a Mediterranean diet assessed via surveys. Feasibility was assessed via a single-item question on each of the online surveys administered while participants were actively receiving intervention of “how easy do you feel this diet is to follow?” on a 0 to 10 numerical score (0 very easy to 10 very difficult) with a score of <5/10 being regarded as reasonably easy to follow. Our predefined feasibility benchmark was as at least three of the four assessments achieving a score of <5/10 on a 0–10 numerical score, with the goal of a Mediterranean diet being at least as easy to follow as the standard USDA.

Adherence was assessed using the 14-point Mediterranean Diet Adherence Screener (MEDAS), included in Supplementary Materials and Methods. We defined good adherence to a Mediterranean style eating pattern as a score of ≥8 on the MEDAS, which is the top tertile and has been used as a benchmark for good adherence in other U.S.-based studies (23). Our predefined goal was to have at least 80% of the participants in the Mediterranean group maintaining a MEDAS score of ≥8 during the active intervention period.

As a second mode of dietary assessment, participants completed online 24-hour diet recalls using the NCI Automated Self-Administered 24-hour (ASA24) dietary assessment tool at unannounced days during weeks 1, 2, 3, 6, 9, 12, and 15 which was used to calculate the USDA Healthy Eating Index 2015 score (HEI-2015; ref. 24); however, these data were used for exploratory observation only. Other exploratory endpoints included plasma concentration of inflammatory cytokines, reduction in symptom burden, changes in hematologic parameters, lipids, and change in the gut microbiome.

Symptom burden was assessed using the myeloproliferative neoplasm symptom assessment form, total symptom score (MPN-SAF TSS) (also known as MPN-10), which grades the 10 most clinically relevant symptoms of patients with MPN (25). We supplemented this questionnaire with extended gastrointestinal focused symptoms (survey available in Supplementary Materials and Methods).

### Study Schedule

**FIGURE 1:**
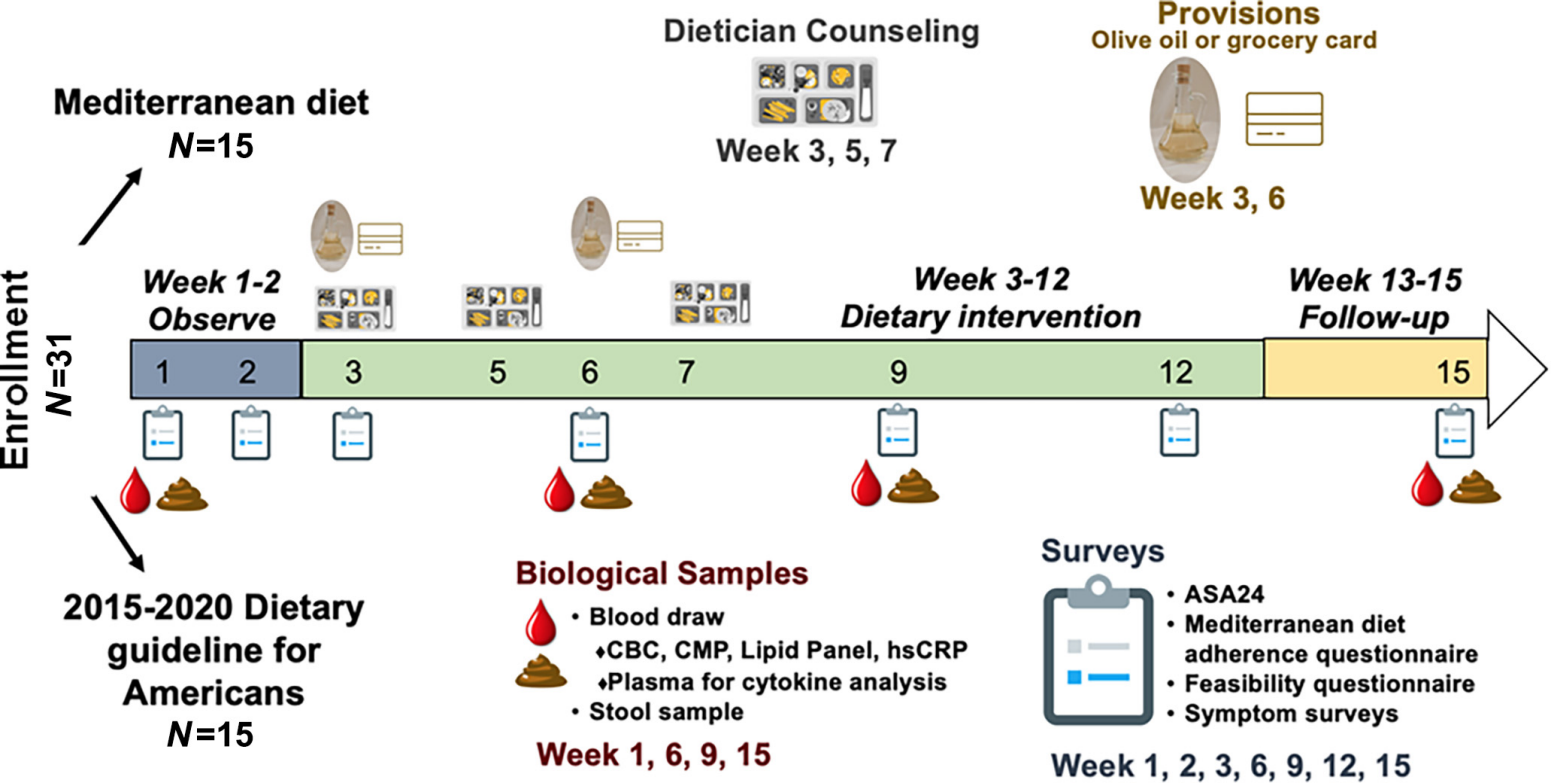
NUTRIENT study design.

The total duration of the study was 15 weeks (Fig. 1). During weeks 1–2, participants were followed without intervention, during which we obtained two baseline measures of dietary intake and symptom burden (one at enrollment and a second unannounced one during the 2-week lead-in time) and one biological sample (blood and stool). Week 3–12 was the active intervention period, which included registered dietician (RD) counseling and written educational materials, and completion of four surveys, four dietary recalls, and collection two biological samples (stool and blood). Week 13–15 was the follow-up period where participants no longer received weekly educational materials. At week 15, participants completed one survey, one 24-hour dietary recall, and contributed one set of biological samples (blood, stool).

### Intervention

Participants were told they would be randomized to one of two diets that are conventionally regarded to be healthful but were not informed of the specific diets being studied nor which group they were randomized to. The USDA was chosen as an intervention that would provide the participants with equal counseling attention but did not encourage a Mediterranean diet eating pattern.

All participants met once at the start of the intervention period (week 3) with a RD for one-on-one counseling to educate the participant on the central components of the MED or the USDA and to tailor the diet to meet each participant's medical needs and/or cultural preferences. Participants had two follow-up dietary counseling visits during weeks 5 and 7. Participants were emailed 10 weekly installments of educational materials on their respective diet in a pdf format during week 3–12 (Supplementary Materials and Methods). All participants in the MED arm were gifted 750 mL of EVOO at weeks 3 and 6, and all participants following the standard U.S. Guidelines diet were given a $10 grocery gift card at weeks 3 and 6. The participants were encouraged to utilize the EVOO but this was not a requirement.

### Data Collection

#### Patient Reported Outcomes Instruments

Copies of all instruments used are included in Supplementary Materials and Methods. Surveys were administered online using Qualtrics for ease of capturing data electronically; however, paper surveys were also available for participants to use if preferred.

#### Laboratory Studies

Four biological sample datapoints were collected during the 15-week study which included collection of blood, stool, and urine. Peripheral blood was collected at weeks 1, 6, 9, and 15, complete blood count (CBC), comprehensive metabolic panel, lipid panel, and high-sensitivity C-reactive protein (hs-CRP) were performed by the clinical laboratory of UCI Health. Blood was centrifuged within 2 hours of draw to obtain plasma and stored at −80°C for cytokine measurements.

#### Cytokine Analysis

Frozen plasma was performed by Quanterix in Billerica, MA for analysis. A Human CorPlex 10 Cytokine Array kit #85-0329 (IL12p70, IL1B, IL4, IL5, IFNɣ, IL6, IL8, IL22, TNFα, and IL10) was used according to manufacturer's protocol and analyzed using a Quanterix SPX imager system on-site at Quanterix Headquarters in Billerica, MA. Cytokines were transformed to a log base 2 scale for analysis purposes. Mixed models (with a random intercept for each participant) were used to explore changes over time where group and time were fixed effects and an interaction term was included.

#### Collection of Fecal Samples

Study participants provided a fecal sample at four different timepoints (weeks 1, 6, 9, and 15) stored in Zymo DNA/RNA shield preservation buffer (catalog no. R1101). These were returned in person or by mail. Samples were then stored at −80°C until analysis.

#### Extraction of DNA from Fecal Samples

DNA was extracted from feces by thawing the samples on ice and homogenizing them. Afterward, 1 mL of the fecal slurry was extracted using the ZymoBiomics DNA Miniprep Kit (catalog no. D4300) in accordance with the manufacturer's suggested protocol. Bead lysis during the extraction was performed at 6.5 m/s for 5 minutes total using a MPBio FastPrep-24 instrument.

#### Library Preparation and Sequencing

Libraries for shotgun metagenomic sequencing of extracted fecal DNA were prepared using the Illumina DNA prep kit (catalog no. 20018705), using an adapted low-volume protocol (26). DNA quantification of the final library pool was performed using the Quanti-iT PicoGreen dsDNA kit (catalog no. P7589). Synthetic microbial DNA standards were included as positive sequencing controls (ZymoBIOMICS Microbial Community DNA Standard, catalog no. D6305), and PCR grade water was used as a negative sequencing control. Sequencing was performed by Novogene Corporation Inc. using Illumina's Hiseq 4000. An average of 2,819,107 ± 670,543 paired-end reads per sample, 150 bases in length, was obtained. Data are available on the Sequence Read Archive (SRA) under the BioProject ID, PRJNA918651.

#### Microbiome Analysis

First, raw sequencing data were quality filtered, and host-derived reads were removed. Taxonomic assignment of sequences was performed using MetaPhlAn3 and its default parameters (27). A table of species relative abundances per sample was produced and subsequently analyzed in R v4.2.1. The Shannon diversity index, Bray–Curtis dissimilarity matrix, and principal coordinate ordination were performed using the Vegan v2.5-6 package in R (28). Significance testing of microbiome diversity and composition metrics was done using linear mixed effect models in the nlme v3.1-148 package (29). Further details regarding the analysis of microbiomes can be found in our companion article (30). The code used for this analysis is available at https://github.com/Javelarb/MPN_diet_intervention.

### Data Availability

Raw data are available upon request by emailing corresponding author Angela Fleischman at agf@uci.edu. Sequencing data are available under SRA Accession PRJNA647720 “Human gut microbiome sequencing during a high-fiber diet intervention”.

## Results

### Adoption of a Mediterranean Eating Pattern

**FIGURE 2:**
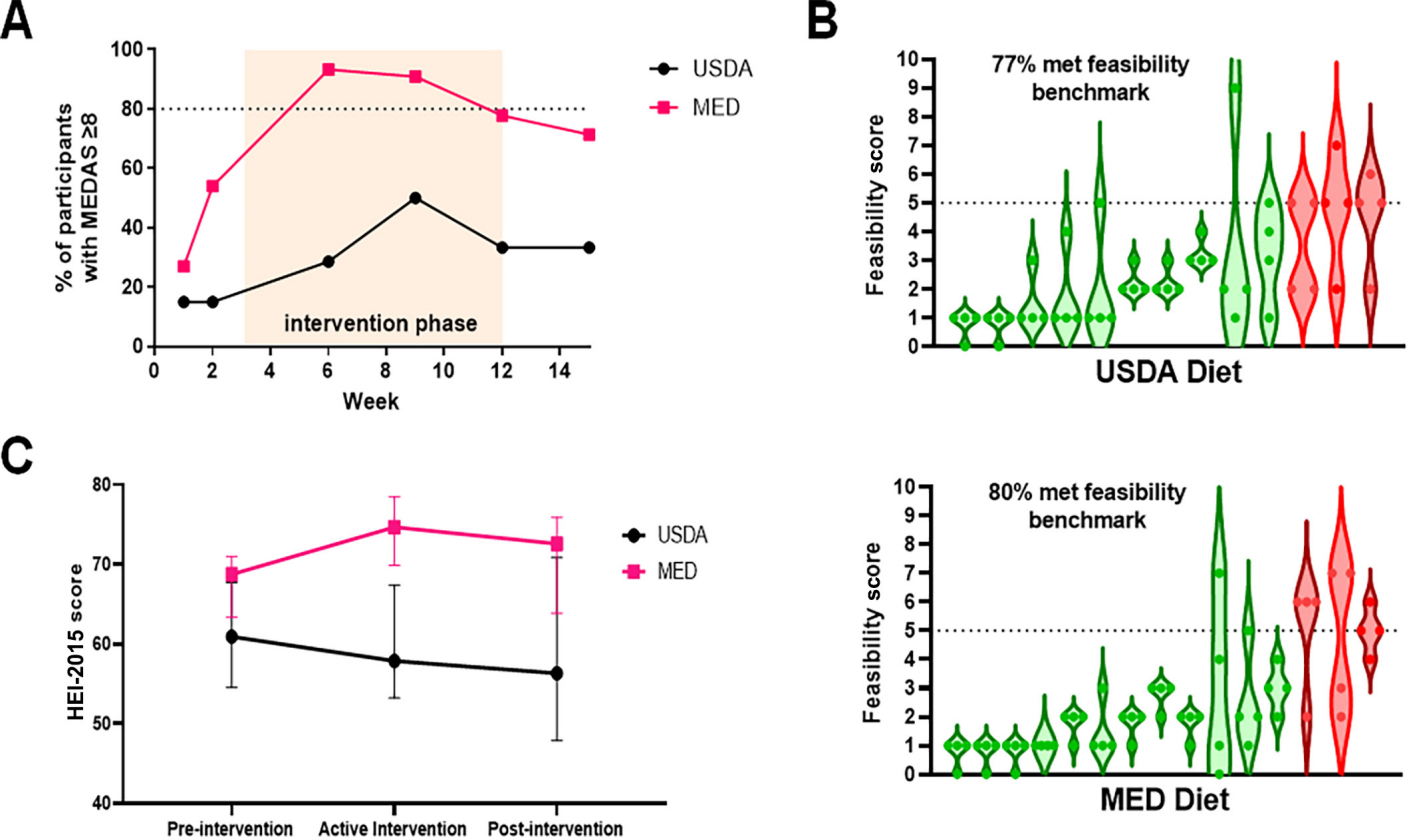
Patients with MPN can adopt a Mediterranean eating pattern with dietician counseling and education. **A,** Percentage of participants with MEDAS scores ≥8 at each timepoint with orange shaded area depicting the active intervention period. **B,** Participant responses to feasibility question during active intervention period, green represents a participant who met the feasibility benchmark of HEI-2015 (**C**) was calculated from each 24-hour diet recall, and scores for each participant were averaged for the pre-intervention (weeks 1–2), active intervention (weeks 3–12), and post-intervention (weeks 13–15) period. Data shown represent median with interquartile range.

Adherence to a Mediterranean style eating pattern was assessed at weeks 1, 2, 3, 6, 9, 12, and 15 using a 14-point MEDAS (31). Because of inconsistent timing of the week 3 surveys with relation to the initial dietician visit, this timepoint was removed from the analysis. A MEDAS score of ≥8 was defined as having adequate adherence to a Mediterranean style eating pattern (23). At weeks 6 and 9, ≥80% of the MED group maintained a MEDAS score of ≥8. At week 12, 79% of the MED group maintained a MEDAS score of ≥8 which was slightly below our predefined goal of 80%. In contrast, at no timepoint did at least 80% of the USDA group achieve a MEDAS score of ≥8 (Fig. 2A). These data demonstrate that patients with MPN can adopt a Mediterranean eating pattern with dietary counseling; however, ongoing education may be necessary for them to maintain high adherence. These data also highlight that the adoption of a Mediterranean diet eating pattern was specific to those who received Mediterranean diet education rather than general dietary guidance.

An easy to follow facilitates ongoing adherence. We assessed feasibility of each diet with a single question, “how easy do you feel this diet is to follow?” on a 0 to 10 numerical score (0 very easy to 10 very difficult). Seventy seven percent of the patients on the USDA diet achieved our predefined feasibility benchmark, and 80% of patients in the Mediterranean arm achieved this feasibility benchmark (Fig. 2B). This demonstrates that a Mediterranean diet is at least as easy to follow for patients with MPN as the standard USDA.

### Diet Quality of Participants

We used the 24-hour diet recall data (ASA24) to calculate the HEI-2015 (24) as a measure of general diet quality. The median HEI-2015 score for the MED group rose from 69 pre-intervention to 75 during the active intervention and maintained at 73 post-intervention. The median HEI-2015 scores for the USDA group was 61 pre-intervention, 58 during the active intervention, and 56 post-intervention (Fig. 2C). We also measured total caloric intake and percentage of calories from fat, protein, and carbohydrates at baseline, weeks 9, 12, and 15 (Table 2).

**TABLE 2:** Energy intake, fiber intake, and percentage of calories from carbohydrates, sugar, protein, and fat calculated from 24-hour diet recalls. Data are represented as mean ± SD

	USDA	MED
Energy intake (kcal/day)		
Baseline	1,585±513	1,960±835
Week 6	1,720±574	1,665±645
Week 9	2,104±761	1,736±492
Week 12	1,557±608	2,168±665
Week 15	1,694±687	2,201±1284
Fiber intake (g/day)		
Baseline	20±8	26±10
Week 6	24±12	26±9
Week 9	24±12	29±13
Week 12	18±9	33±18
Week 15	21±9	29±18
Energy change from baseline		
Week 6	77±668	−295±604
Week 9	519±644	−224±690
Week 12	−147±543	207±543
Week 15	109±534	240±721
Carbohydrate (% of energy)		
Baseline	36±13	42±9
Week 6	42±9	47±9
Week 9	50±14	46±13
Week 12	40±12	42±10
Week 15	45±11	42±7
Sugar (% of energy)		
Baseline	15±6	17±5
Week 6	21±14	18±5
Week 9	16±7	19±13
Week 12	16±6	17±6
Week 15	15±8	14±5
Protein (% of energy)		
Baseline	19±9	17±5
Week 6	17±6	17±6
Week 9	14±4	20±8
Week 12	18±4	18±6
Week 15	18±7	17±5
Fat (% of energy)		
Baseline	46±12	40±9
Week 6	42±9	36±8
Week 9	35±12	35±11
Week 12	41±10	39±10
Week 15	37±8	41±8

### Impact of Diet on Symptom Burden

**FIGURE 3:**
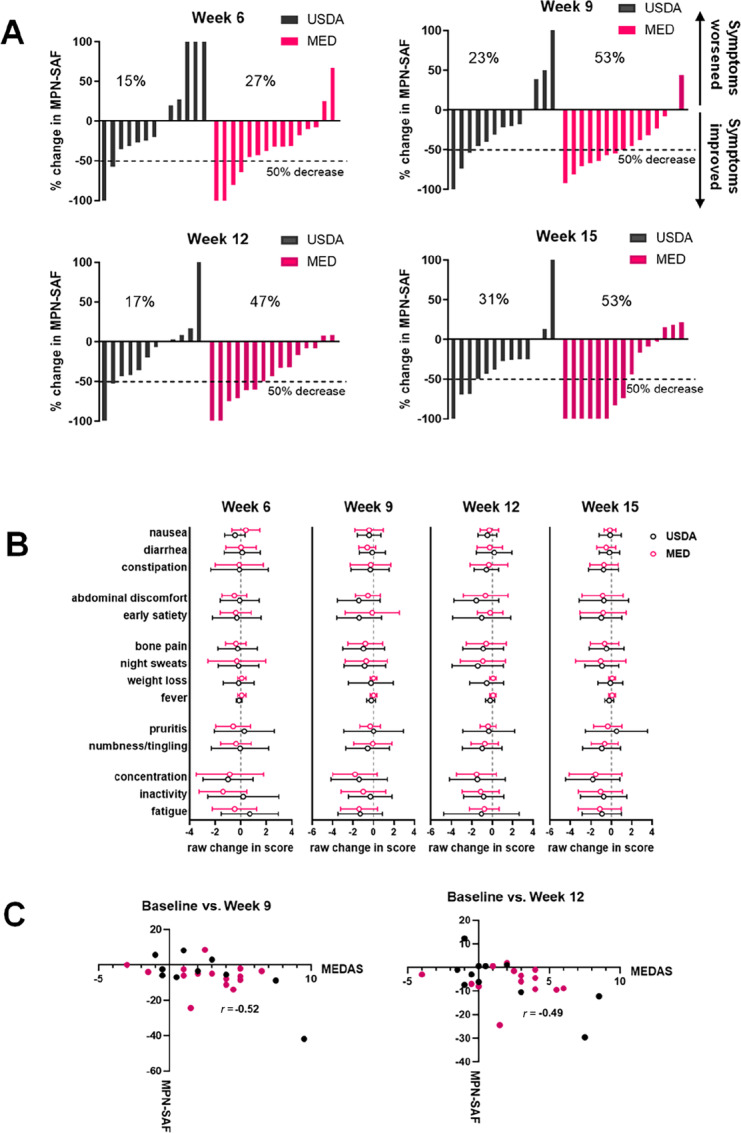
Changes in symptom burden during study. **A,** Waterfall plots of percentage change in MPN-SAF (MPN-SAF TSS) at each week compared with baseline (baseline defined as average MPN-SAF TSS of weeks 1 and 2). **B**, Raw change in specific symptoms at each week compared with baseline (mean ± SD). **C,** Correlation between change in raw MEDAS score and change in raw MPN-SAF score from baseline to week 9 (left) or baseline to week 12 (right).

At weeks 9, 12, and 15, 53%, 47%, and 53% of the MED cohort achieved a >50% reduction in their MPN-SAF TSS. In comparison, at weeks 9, 12, and 15, 23%, 17%, and 31% of the USDA cohort achieved a >50% reduction in their MPN-SAF TSS (Fig. 3A). We also calculated the mean change in each specific symptom queried on the surveys to visualize the impact of the diets on specific symptoms (Fig. 3B). To determine whether movement toward a Mediterranean eating pattern is associated with an improvement in symptoms a Pearson correlation coefficient was computed to assess the linear relationship between the change in MEDAS score from baseline and change in MPN-SAF score from baseline at weeks 9 and 12 from the entire cohort. There was a negative correlation between the two variables at week both week 9, *r*(24) = −0.52, *P* = 0.007 and week 12, *r*(24) = −0.49, *P* = 0.012 (Fig. 3C), demonstrating a correlation between improvement in MEDAS score and reduction in MPN-SAF.

### Impact of Diet on BMI and Laboratory Parameters

**FIGURE 4:**
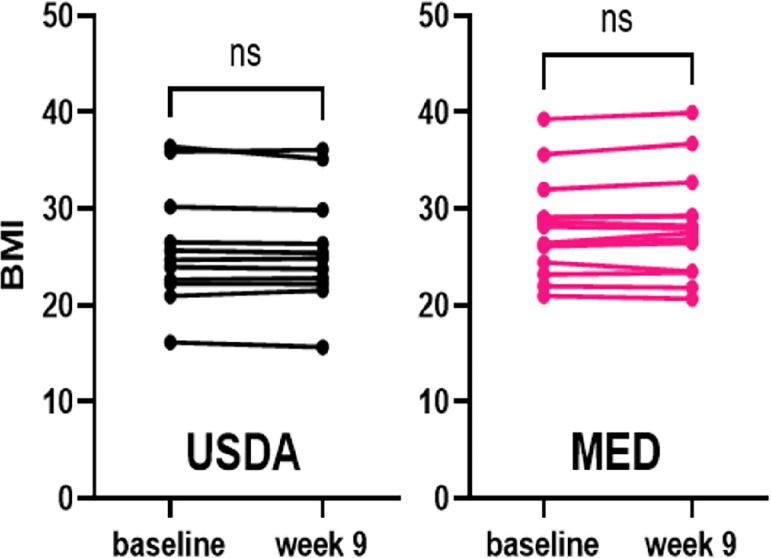
Baseline and week 9 BMI. Baseline BMI was calculated using height and weight at enrollment, with no significant differences between USDA and MED groups. Weight was followed throughout the study, no significant changes in BMI were observed in either group.

Weight loss or gain toward an ideal body mass index (BMI) was not a goal of the dietary counseling; however, we measured baseline BMI with height and weight at baseline and followed weight at each visit. Baseline BMI was not significantly different between the MED and USDA groups. Neither group experienced a significant change in BMI at week 9 compared with baseline (Fig. 4).

Laboratory data, including complete blood counts with differential (CBC w/diff; Supplementary Fig. S1), comprehensive metabolic panel (Supplementary Table S3), and lipid profiles (Supplementary Table S4) were collected at week 1 (pre-intervention), weeks 6 and 9 (during active intervention), and week 15 (post-intervention). There was a median change in low-density lipoprotein of −13 g/dL in the MED group and +2 g/dL in the USDA group at week 9 versus baseline. Blood counts, kidney function, and liver function remained stable during the intervention, demonstrating that a diet intervention is safe in the MPN patient population.

### Impact of Diet on Inflammatory Biomarkers

**FIGURE 5:**
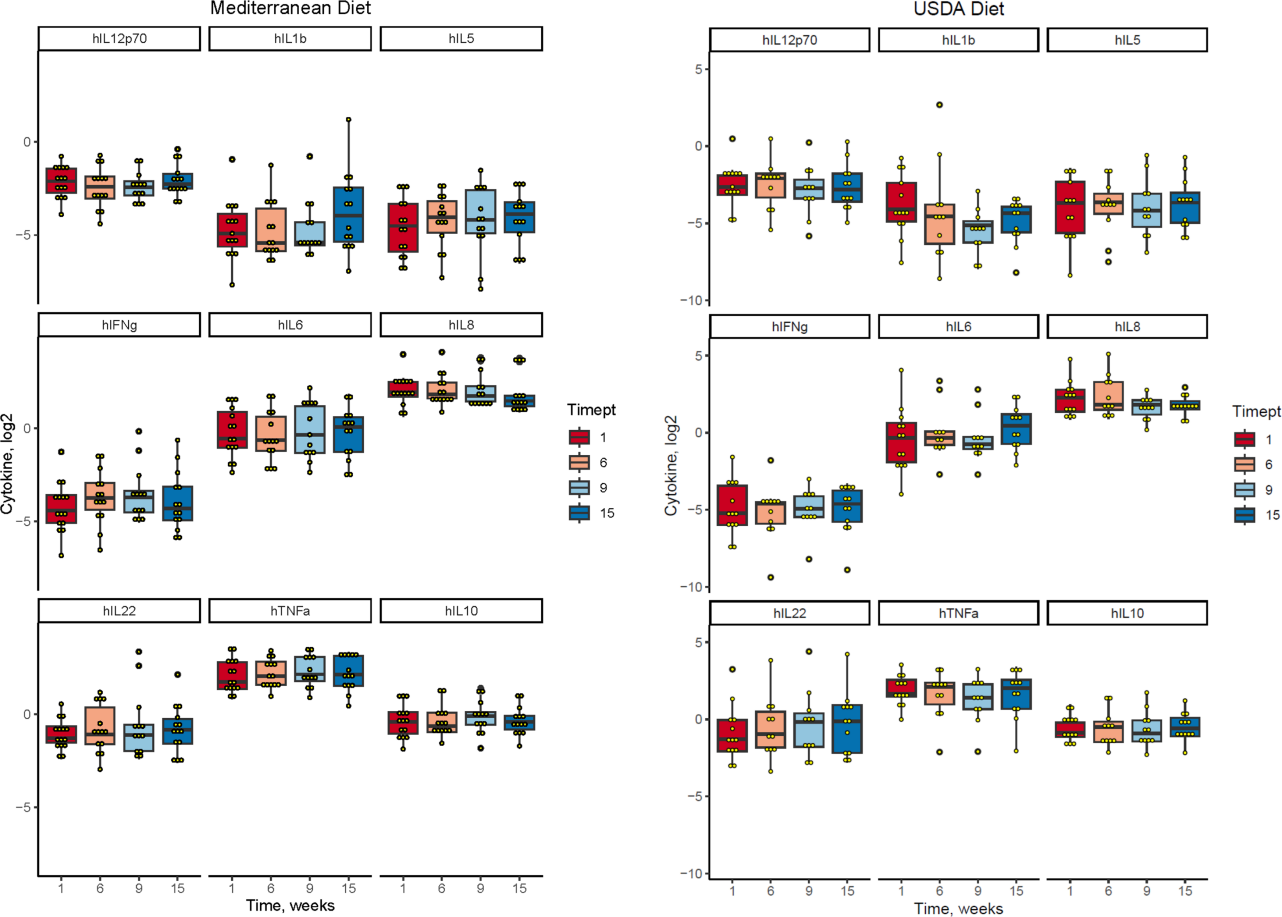
Changes in plasma cytokine concentrations throughout the study. Plasma cytokines were measured at weeks 1, 6, 9, and 15.

We explored the impact of the diet intervention on biological measures of inflammation, including hs-CRP (Supplementary Fig. S2) and plasma cytokines. Out of the 10 cytokines measured, five cytokines (TNFα, IL6, IL8, IL10, IL22) yielded levels within the detectable range (Fig. 5). In these five cytokines, there were no significant differences in changes over time by group.

### Impact of Diet on Gut Microbiome

**FIGURE 6:**
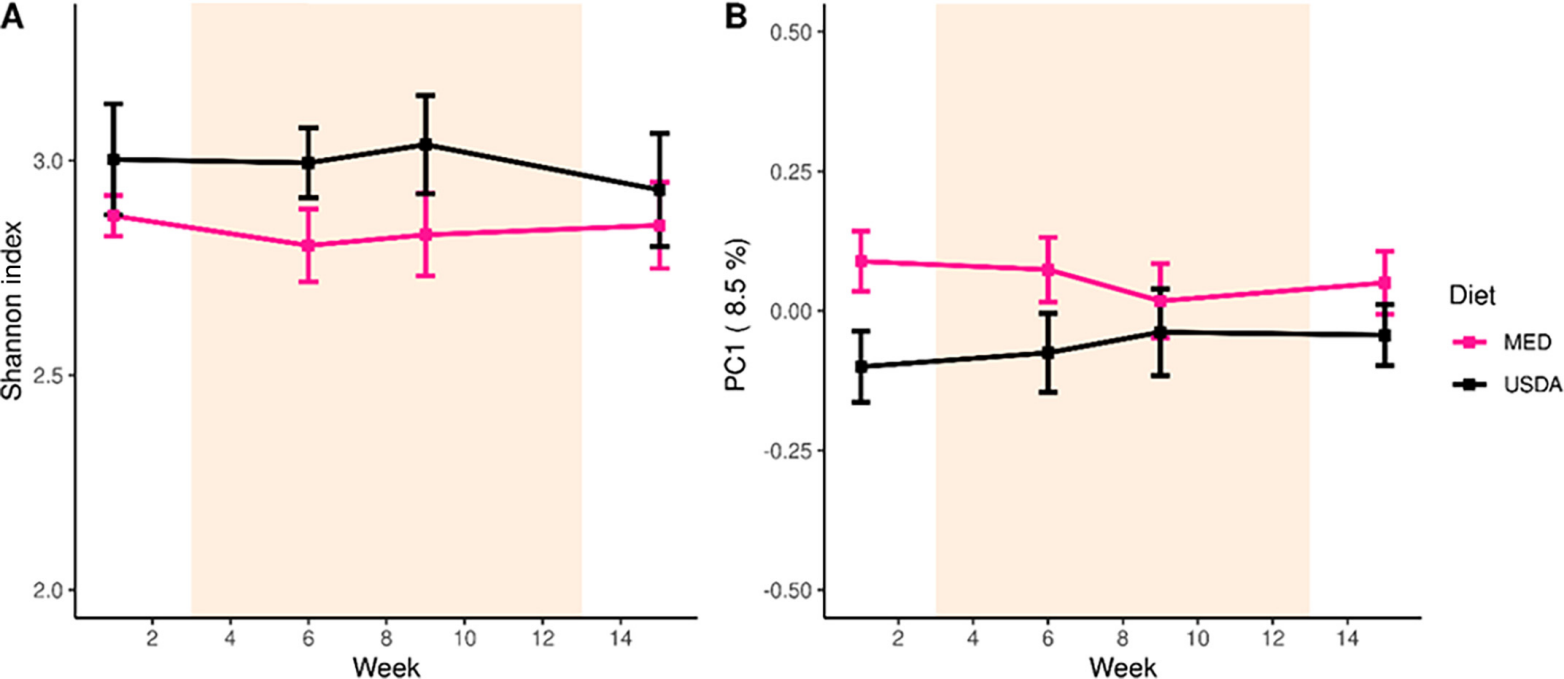
Fecal microbiome composition. **A,** A line plot displaying the microbial diversity, as measured by the Shannon index, of individuals over time. **B,** A line plot displaying the microbial composition of individuals over time. The *y*-axis is the first principal coordinate produced by Bray–Curtis dissimilarity ordination of the microbiome. In both A and B, the orange shaded area depicts the active intervention period and the SE is represented by error bars.

We used shotgun metagenomic sequencing to evaluate the gut microbiome at weeks 1 (pre-intervention), weeks 6 and 9 (during active intervention), and week 15 (post-intervention). We found that microbiome diversity and composition was stable throughout the study duration in both cohorts, with no differences in the Shannon diversity index (*P* = 0.57) or first principal coordinate of microbiome composition (*P* = 0.25) due to diet (Fig. 6). A more detailed analysis of the microbiome of this cohort and correlations with cytokines are described in a separate article (30).

### MPN Driver Mutation Allele Burden

We quantified the *JAK2^V617F^* allele burden of JAK2-positive patients from whole blood during the trial using digital PCR. We observed minimal changes of the *JAK2^V617F^* allele burden in both diet groups (Supplementary Fig. S3) over the short duration of the study.

## Discussion

The central purpose of the NUTRIENT trial was to establish the feasibility of diet as a therapeutic approach in MPN. Diet may be a low-risk, low-cost approach to reduce inflammatory cytokines thus alleviating symptoms and potentially preventing disease progression. In addition, empowering patients with MPN to take an active role in their treatment through diet may instill a greater sense of well-being and improve quality of life. Demonstrating feasibility builds a foundation on which to build much larger interventional studies to rigorously assess the impact of diet on clinical outcomes in MPN.

The Mediterranean diet was chosen because it is generally accepted to be a healthful diet, is rich in anti-inflammatory and antioxidant compounds, and has been found to reduce inflammatory biomarkers (19). Patients with MPN reported that a Mediterranean diet program was just as easy to follow as a program based on the US Guidelines for Americans (Fig. 2). This is important because for long-term adherence a diet must be easy to follow.

Using the MEDAS as the primary measure to quantify adherence to a Mediterranean style eating pattern the Mediterranean diet group met the predefined goal at weeks 6 and 9 but dipped slightly below the goal adherence threshold at week 12. In contrast, at no timepoint did the USDA group meet the goal adherence. This demonstrates that patients with MPN can adopt a Mediterranean diet eating pattern with dietician counseling and written curriculum. In our study dietician counseling was concentrated at the beginning of the intervention period which likely explained why adherence to a Mediterranean eating pattern waned toward the end of the intervention period. Longitudinal engagement with Mediterranean education may be necessary for patients with MPN to maintain good adherence to a Mediterranean diet eating pattern. We are currently developing a multi-modality Mediterranean diet curriculum structured as a course, including online lectures, knowledge checks, recipes and meal plans, group classes, and one on one dietician counseling to be used in subsequent trials.

Targeting symptoms is an important goal in MPN, as symptoms negatively impact quality of life, increase use of medical care, and result in loss of productivity. Although not statistically powered to detect a change, we explored the impact of a diet intervention on symptom burden. A general improvement in diet quality would be expected to lead to an improvement in well-being which would translate into a reduction in symptom score. Participants in both groups enjoyed a reduction in symptom burden. In the USDA group, 15%, 23%, 17%, and 31% had a >50% reduction in their MPN-SAF TSS score at 6, 9, 12, and 15 weeks, respectively. In the MED group 27, 53, 47, and 53% had a >50% reduction in their MPN-SAF TSS at 6, 9, 12, and 15 weeks. These data suggest that a general improvement in diet quality can impact symptoms, but that the components of a Mediterranean diet may augment symptom improvement.

The length of the diet intervention and intensity of sessions may be an important factor in creating a change in eating habits of participants. Our active intervention period consisted of 10 weeks, with an additional 2-week lead-in time and 3 weeks post-intervention follow-up. For dynamic endpoints such as symptom burden, it appears that a 10-week intervention is sufficient to detect change. However, with a longer intervention period one could examine whether there is sustained improvement in symptoms. A longer intervention period may also identify a subset of people with delayed symptom improvement, potentially revealing that symptoms stem from different root causes, some of which are quickly changed and others of which take some time to change.

The lack of identifying a reduction in inflammatory cytokines was not surprising given our small sample size. Future larger studies are required to assess the impact on inflammatory cytokines, in addition potentially longer time periods are needed to observe decreases in inflammatory cytokines. Or, perhaps alternative approaches to capture and quantify inflammation are required.

This trial afforded us the opportunity to explore potential changes in the gut microbiome over time with a diet intervention. Although the microbiome remained stable throughout the study in both cohorts, a Mediterranean diet has been shown to beneficially alter the gut microbiome over time (32). In addition, the polyphenolic compounds found in EVOO select for microbes associated with reduced inflammation (33, 34). This suggests that adherence to a MED diet may indeed be impactful on the gut microbiome of patients with MPN and is worthwhile to evaluate in a larger diet intervention cohort.

Limitations of this study include a small patient number, the limited follow-up and other confounders that maybe contributing. However, we could establish that a Mediterranean diet intervention is feasible in the MPN patient population. This population is receptive to using diet as a therapeutic approach and can alter their diet toward a Mediterranean diet eating pattern. The benefits of diet may be seen most when the intervention is started early, for example in patients with low-risk PV or ET who are commonly not given MPN specific therapy. Diet may also be a useful intervention in precursor conditions such as clonal hematopoiesis of indeterminate potential (CHIP), acting not only to ameliorate the negative health consequences of CHIP but to also prevent progression to hematologic malignancy.

## Supplementary Material

Mediterranean Diet education materials Educational materials given to MED diet group

Supplementary Figure 1 Supplementary Figure 1. Complete blood count (CBC) data from participants.

Supplementary Figure 2 Supplementary Figure 2. Changes in hsCRP during the study

Supplementary Figure 3 Supplementary Figure 3. Changes in JAK2V617F allele burden over time.

Supplementary Table 1 Supplementary Table 1. Inclusion and Exclusion Criteria

Supplementary Table 2 Supplementary Table 2. Representativeness of Study Participants.

Supplementary Table 3 Supplementary Table 3. Longitudinal complete metabolic panels of participants.

Supplementary Table 4 Supplementary Table 4. Longitudinal lipid values.

Surveys Surveys used in the study

USDA diet educational materials Education materials given to the USDA group
