# Supplementary material for: The NUTRIENT Trial (NUTRitional Intervention among myEloproliferative Neoplasms): Results from a Randomized Phase I Pilot Study for Feasibility and Adherence
Source: Cancer Res Commun. 2024 Mar 5;4(3):660–70. doi: 10.1158/2767-9764.CRC-23-0380 (PMC10913729; doi:10.1158/2767-9764.CRC-23-0380)
Supplement: Mediterranean Diet education materials — Educational materials given to MED diet group [file crc-23-0380-s01.pdf]

Healthy Mediterranean Diet for MPN Patients

Week 1

Extra Virgin Olive Oil

Olive oil is used from lip balm to salad dressing! Extra virgin olive oil can give your cooking a unique aroma and flavor.

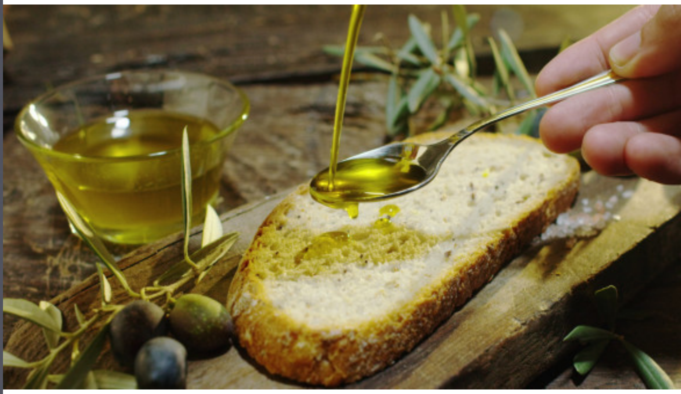

Extra virgin olive oil is a good source monounsaturated fat also known as "good fat" and high in antioxidants.

Recipe:

Balsamic Vinegar and Olive Oil Dressing

You will need:

- A jar
- 1/2 cup extra virgin olive oil
- 1/4 cup balsamic vinegar
- 1 teaspoon honey
- 1 teaspoon Dijon mustard
- 1 shallot, minced
- 1 clove garlic, minced
- salt and ground pepper to taste

Servings: 8
Prep time: ~10 minutes

- Combine the olive oil, balsamic vinegar, honey, Dijon mustard, shallot, garlic, salt, and black pepper together in a jar or container with good-sealing lid.
- Screw on lid and shake vigorously.
- Adjust to taste.
- Refrigerate or enjoy immediately!
- Contents will separate after a few minutes, shake to recombine oil and vinegar.

Fact #1

Olive Oil vs. Extra Virgin Olive Oil

Virgin or extra virgin olive oil is derived from simply pressing the olives. Olive oil marketed as solely "olive oil", "pure", or "light" undergoes additional refining similar as to the process of making lower quality oils such as Canola.

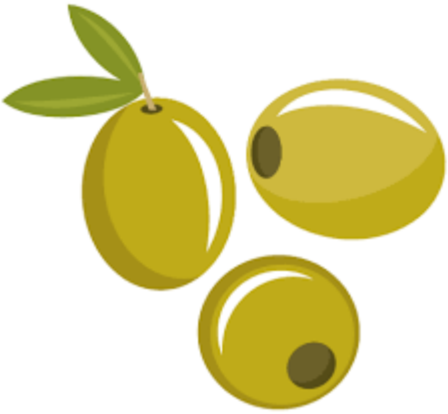

We are MPN

HEALTH TIP!

Olive oil is extremely high in oleic acid which is used to reduce blood pressure. Olive oil also contains antioxidants including vitamin E, carotenoids and oleuropein.

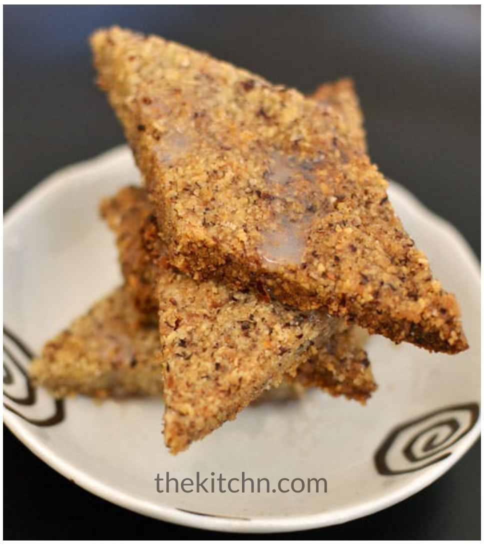

Recipe:

Hazelnut & Olive Oil Shortbread

You will need:

- 1 1/4 cup hazelnut meal
- 3/4 cup flour
- 1/4 cup brown sugar
- 1/4 cup powdered sugar, plus 1/4 cup for glaze
- 1 teaspoon kosher salt
- 1 lemon, zested and juiced
- 1 teaspoon vanilla
- 1/2 cup extra-virgin light olive oil

- Heat the ove not 375F. Whisk together the hazelnut meal, flour granulated sugar, 1/4 cup powdered sugar, salt and lemon zest. Whisk in the vanilla and olive oil. The dough will be sandy and quite crumbly.
- Press the dough firmly into a 8x8-inch (or 9x9-inch) dish. Bake for 20 minutes or until just lightly browned around the edges. Immediately ct the shortbread into diamonds or squares. Let cool completely before lifting them out of the pan, however.
- Meanwhile, whisk together 1 tablespoon of the lemon juice and the remaining 1/4 cup powdered sugar and drizzle over the warm cookies.

By Faith Durand
@thekitchn.com

Fact #2

The fresher the olive oil the better the taste

California Olive Ranch

Unlike wine that can improve with age, olive oil is meant to be consumed fresh. Once you buy it, store it in a dark cool place to keep it fresh. After a year or so, oil is best used for cooking and fresh oil used for salad dressings and other cold uses.

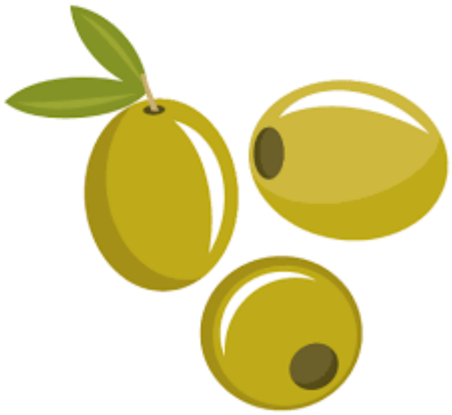

Olive Oil 101

Olive oil has been the hallmark of the healthy Mediterranean Diet for over 2,500 years. It adds vibrant flavors and textures to Mediterranean foods and is high in healthy, monounsaturated fats along with antioxidants. Simply drizzle it on cooked fish or vegetables, or use it as a dip for bread. Vegetables roasted, grilled, or sautéed in olive oil are simply tastier – so you'll find yourself eating more of them!

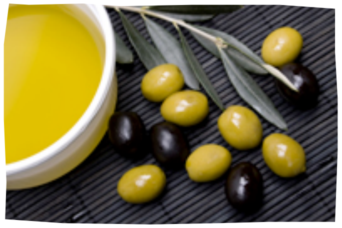

Olive Oil Flavors

Olives are the fruit of the olive tree. Soon after being picked, they're cleaned in a water bath and then crushed into a mash. This mash has three unique parts: olive solids, olive water, and olive oil. First, the olive solids are separated. Next, the olive water and oil are quickly separated to keep the olive water from changing the oil's taste and odor. Finally, the oil is bottled.

The best quality olive oils are obtained from the first pressing of the olives and are "cold pressed." This means they're not heated during the pressing process. Heating produces larger amounts of oil, but decreases important flavor and healthy compounds, including flavenols and polyphenols, abundant in extra-virgin olive oil.

Olive Oil Grades

Olive oil is graded on taste, acidity level, and processing method. The table below lists the main types of olive oil in order of decreasing quality.

Grade	Description	Taste	Uses
Extra Virgin	Highest quality oil made from first pressing with no heat or chemicals	Superior	Dips, salads and drizzled on fish
Virgin	Lacks perfect taste of extra-virgin, but not refined	Good	Frying, grilling and roasting
Olive	Blend of virgin and refined (chemically treated) oils	Lacks Flavor	When flavor is not needed
Lite	The word "lite" means the oil has been refined, not that it is lower fat.	Lacks Flavor	When flavor is not needed
Pomace	Lowest quality made by blending virgin and pomace	Lacks Flavor	Frying or cooking

Buying and Storing Olive Oil

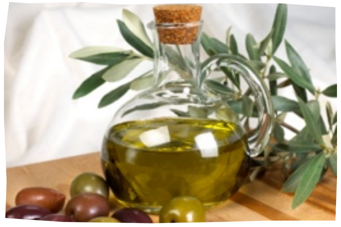

The four foes of olive oil are age, heat, air, and light. When you buy olive oil, make sure it is no more than 18 months old. (Look at the bottling date on the label.) At home, store olive oil in a cool, dark place.

Sauteing with Olive Oil

There's no better way to bring out the flavor of vegetables and seafood than sautéing. It's an easy, healthy way to prepare your favorite dishes. To sauté, pour olive oil into a cold skillet or sauté pan and heat over low heat. When the oil is heated through, add the food item. Stir, toss, or turn until cooked and enjoy!

Baking with Olive Oil

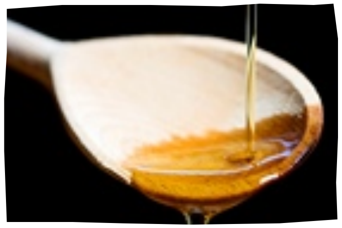

Baking with olive oil, instead of butter, cuts the amount of cholesterol and saturated fat in your favorite recipes. Olive oil produces lighter-tasting breads, brownies, biscotti, and cakes. Even more good news – You need less olive oil than butter when baking! See chart on next page.

Frying with Olive Oil

Frying in olive oil leaves food less greasy, and crunchier, than frying in other fats. Also, foods fried in olive oil have less cholesterol and saturated fat than foods fried in most other fats. Here are some tips when frying with olive oil:

- Deep fry at 350 to 365 °F, and heat the oil slowly.
- Use enough oil to properly cover foods.
- Avoid putting too much food in the oil at once.
- Place food on wire racks after cooking to drain excess fat.

Mediterranean Foods Alliance — An Oldways Program

Copyright 2012 | Oldways | Mediterranean Foods Alliance | 266 Beacon St., Boston, MA 02116 | www.oldwayspt.org

Oldways — Health Through Heritage

Understanding the Pyramid

The Mediterranean Diet Pyramid depicts the traditional foods and drinks that make up the healthy, balanced Mediterranean Diet. It contains many of the foods you will find in other dietary pyramids. The principal difference is in the frequency that some foods are eaten. Almost all foods can be part of a balanced and healthy diet – but your overall health and well-being can be greatly affected by how often you eat different foods, and the portion size you choose.

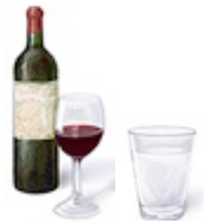

Wine and Water – Wine can be consumed regularly but moderately: up to one glass per day for women, two for men. Water is essential for proper hydration, and contributes to health, well-being, and energy.

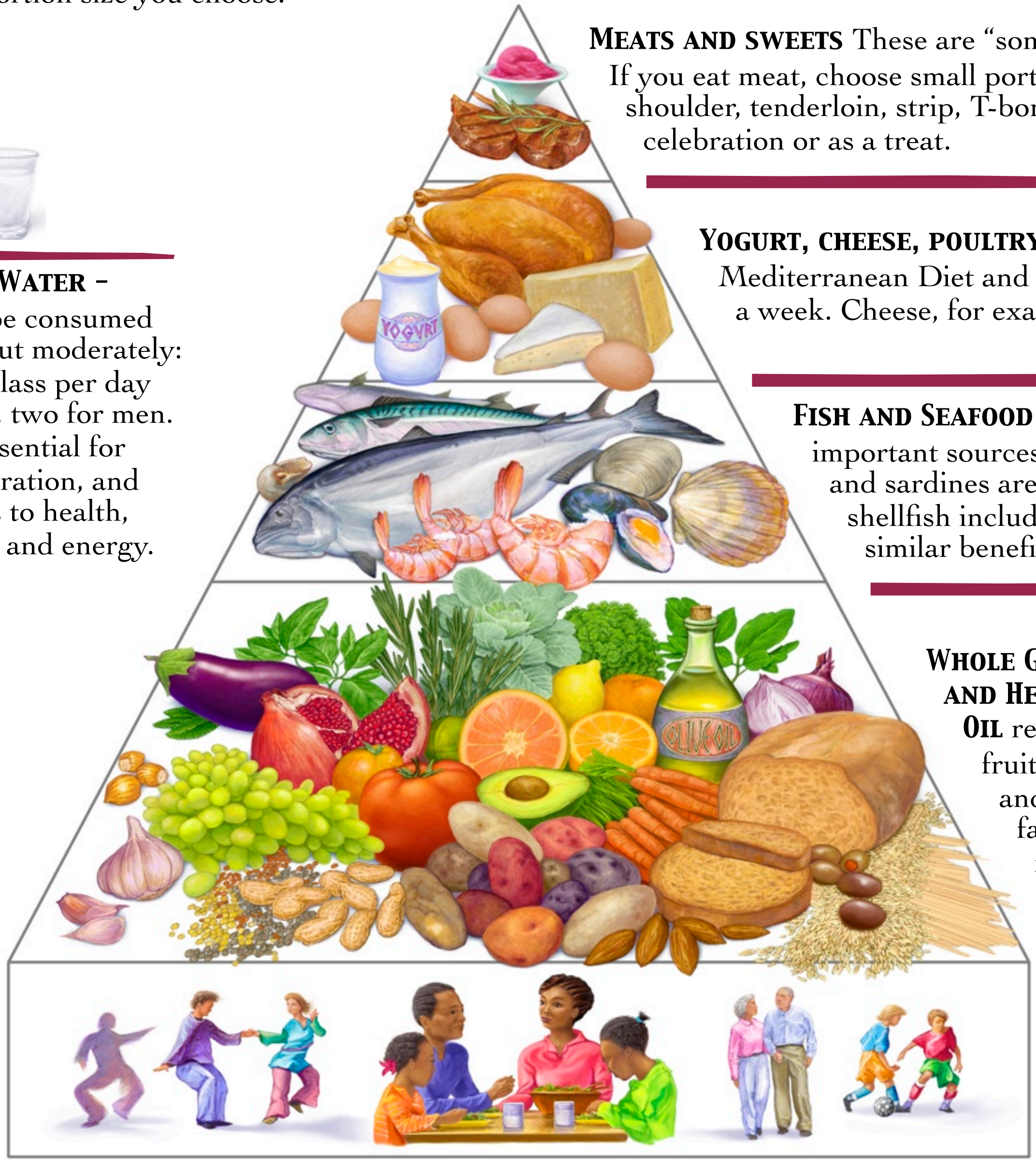

Illustration by George Middleton

© 2009 Oldways Preservation and Exchange Trust • www.oldwayspt.org

Meats and Sweets These are "sometimes" foods to eat less often. If you eat meat, choose small portions of lean cuts, such as round, shoulder, tenderloin, strip, T-bone, and flank. Enjoy sweets at a celebration or as a treat.

Yogurt, cheese, poultry, and eggs form a central part of the Mediterranean Diet and are eaten in moderate portion sizes several times a week. Cheese, for example, is eaten regularly but in small amounts.

Fish and Seafood occupy their own section, since they are important sources of protein. Fish such as tuna, herring, salmon, and sardines are rich in heart-healthy omega-3 fatty acids, and shellfish including mussels, oysters, shrimp, and clams have similar benefits. Enjoy at least twice a week.

Whole Grains, Fruits, Vegetables, Beans, Herbs and Healthy Fats, Such as Those Found in Olive Oil represent the core of the diet. Base every meal on fruits, vegetables, whole grains, legumes, and herbs and spices. Olive oil, the main source of dietary fat, is used for almost all cooking and baking, and for dressing salads and vegetables.

Daily Physical Activity, which is important for overall good health, includes strenuous exercise such as running and aerobics, more leisurely activities such as walking and house-or-yard work, and simple changes, such as taking the stairs instead of the elevator. Add physical activity to each day.

Copyright 2012 | Oldways | Mediterranean Foods Alliance | 266 Beacon St., Boston, MA 02116 | www.oldwayspt.org

THE SCIENCE BEHIND THE DIET

It has been clear for decades that the Mediterranean Diet offers one of the healthiest eating patterns on earth. But why is it so healthy?

Is it the olive oil?

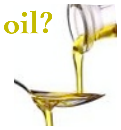

The abundance of leafy greens?

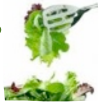

The lack of 24-ounce steaks?

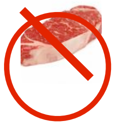

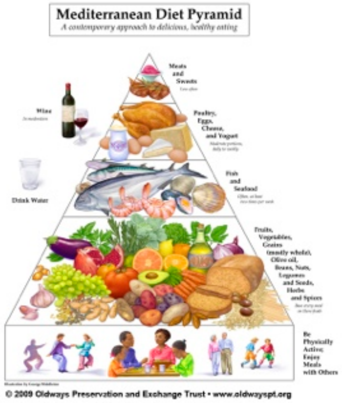

Although research continues to analyze individual foods, it also repeatedly shows that a healthy diet is much more than the sum of its nutrient parts. One early study of the "whole diet" approach was published in 1995 by Antonia Trichopoulou, Walter Willett, Frank Sacks, and others, in which the original Oldways Mediterranean Diet Pyramid was given center stage.

The study documented the health benefits of a diet "characterized by abundant plant foods (fruit, vegetables, breads, other forms of cereals, potatoes, beans, nuts, and seeds) fresh fruit as the typical daily dessert, olive oil as the principal source of fat, dairy products (principally cheese and yogurt), and fish and poultry consumed in low to moderate amounts, zero to four eggs consumed weekly, red meat consumed in low amounts, and wine consumed in low to moderate amounts, normally with meals." In subsequent years the body of scientific evidence supporting the healthfulness of the traditional Mediterranean Diet has continued to grow. See all the latest studies at www.oldwayspt.org.

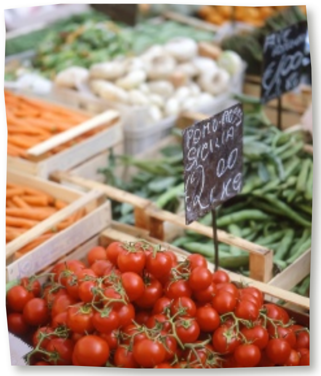

LIVE A LONGER, HEALTHIER LIFE WITH THE MEDITERRANEAN DIET

The Med Diet can help you:

- Lengthen Your Life
- Prevent Asthma
- Fight Certain Cancers
- Protect From Diabetes
- Keep Depression Away
- Prevent Chronic Diseases
- Nurture Healthier Babies
- Ward off Parkinson's Disease
- Safeguard from Alzheimer's Disease
- Aid Your Weight Loss and Management Efforts
- Lower Risk of Heart Disease and High Blood Pressure

Healthy Mediterranean Diet for MPN Patients

Eggplants or Aubergines are purple fruits that have a wide variety of nutritional value such as vitamin C, Potassium, Magnesium, and fiber!

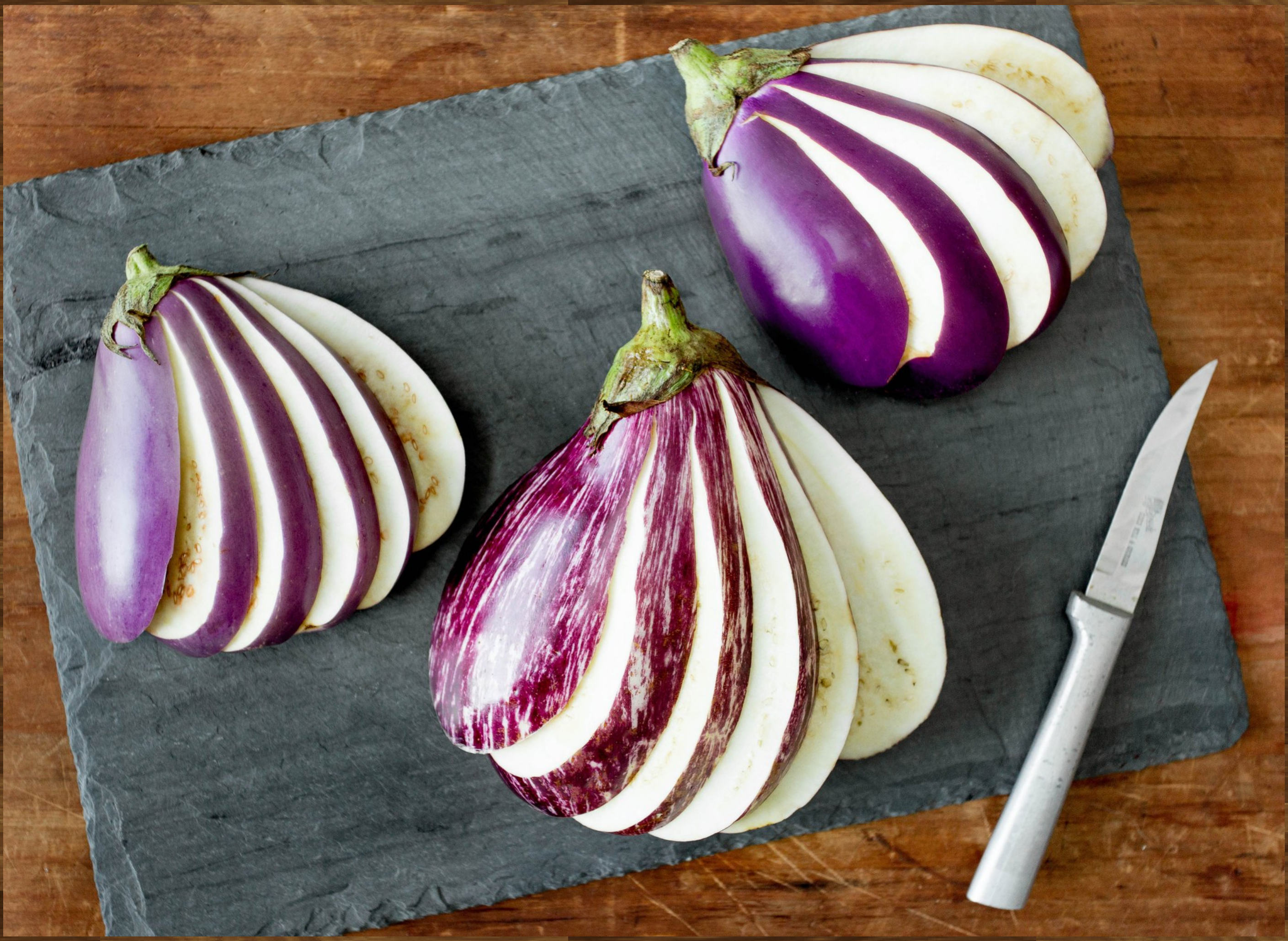

Eggplants are commonly referred to as the "King of Vegetables"- despite being fruits!

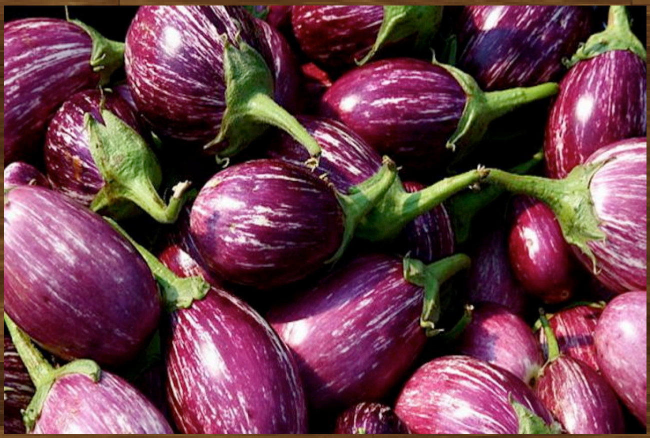

HEALTH TIP!
Eggplant skin is rich in antioxidants so when preparing this recipe, don't skin your eggplants!

02 Week 2: Eggplants

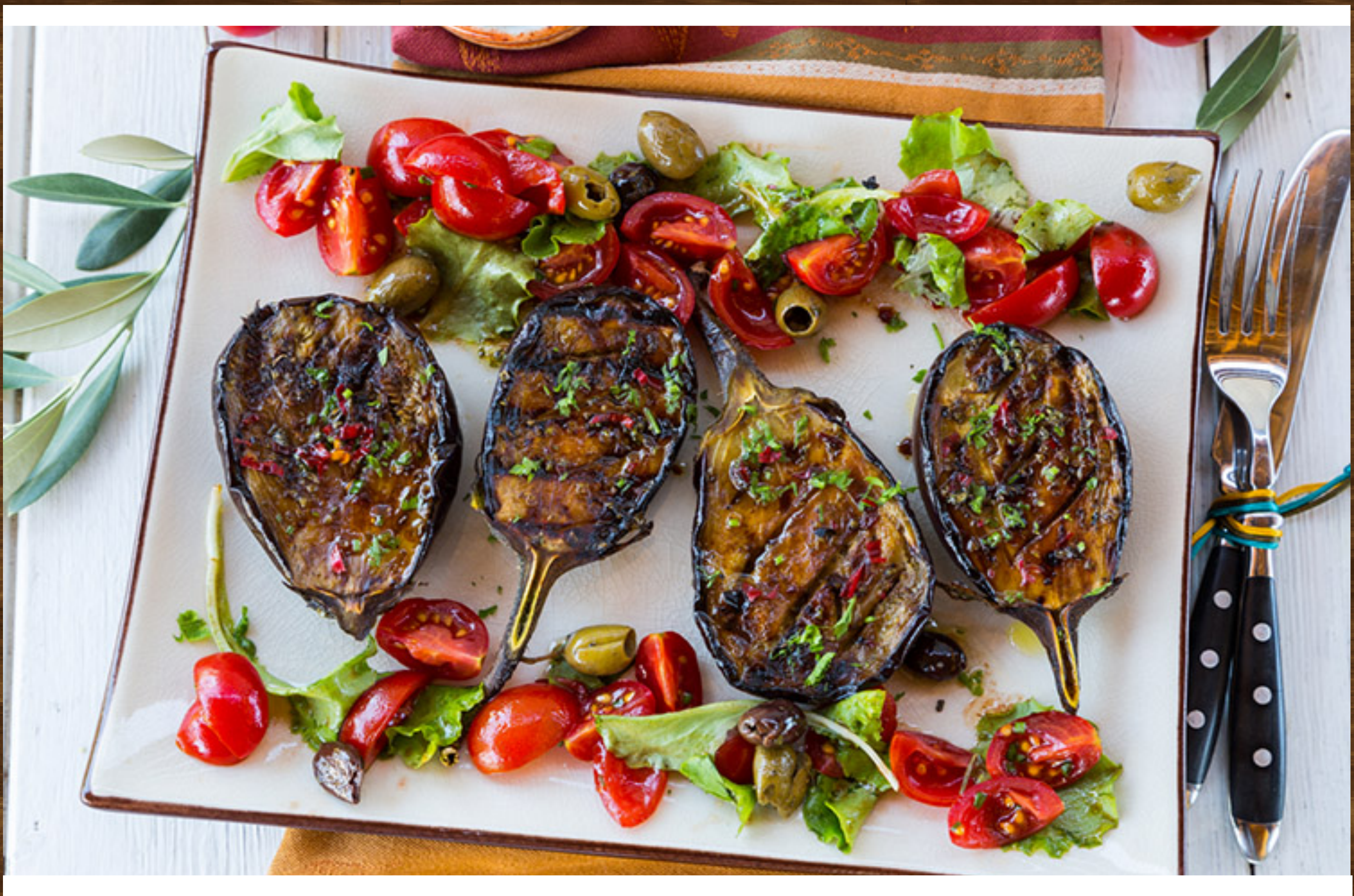

Recipe: Mediterranean Eggplant and Tomato Salad

Directions:

1. Mix 4 tbsp of olive oil, 2 tbsp of balsamic vinegar, 1 tbsp parsley, 1/2 teaspoon garlic, dried oregano, and desired salt into a bowl. Optional: include chili
2. Spread mixture from step 1 over halved eggplants
3. Grill the eggplants until slightly charred
4. While waiting for eggplants, mix all of the tomatoes, olives, leftover olive oil, balsamic vinegar, and desired salt into another bowl.
5. Combine finished eggplant and salad and enjoy!

You will need:

- 4 eggplants, cut lengthwise
- 6 tbsp extra virgin olive oil
- 5 tbsp balsamic vinegar
- 1 finely diced garlic clove
- 1 teaspoon dried oregano
- 2 tbsp chopped parsley
- 4 cups of sliced cherry tomatoes
- 2 cups of choice lettuce
- 1/4 cup pitted olives
- Salt
- Optional: 1 deseeded and finely diced chili

Mediterranean Plate

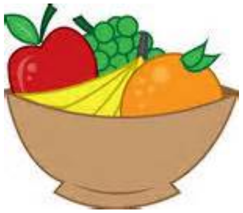

Choose fresh fruit for dessert or snacks

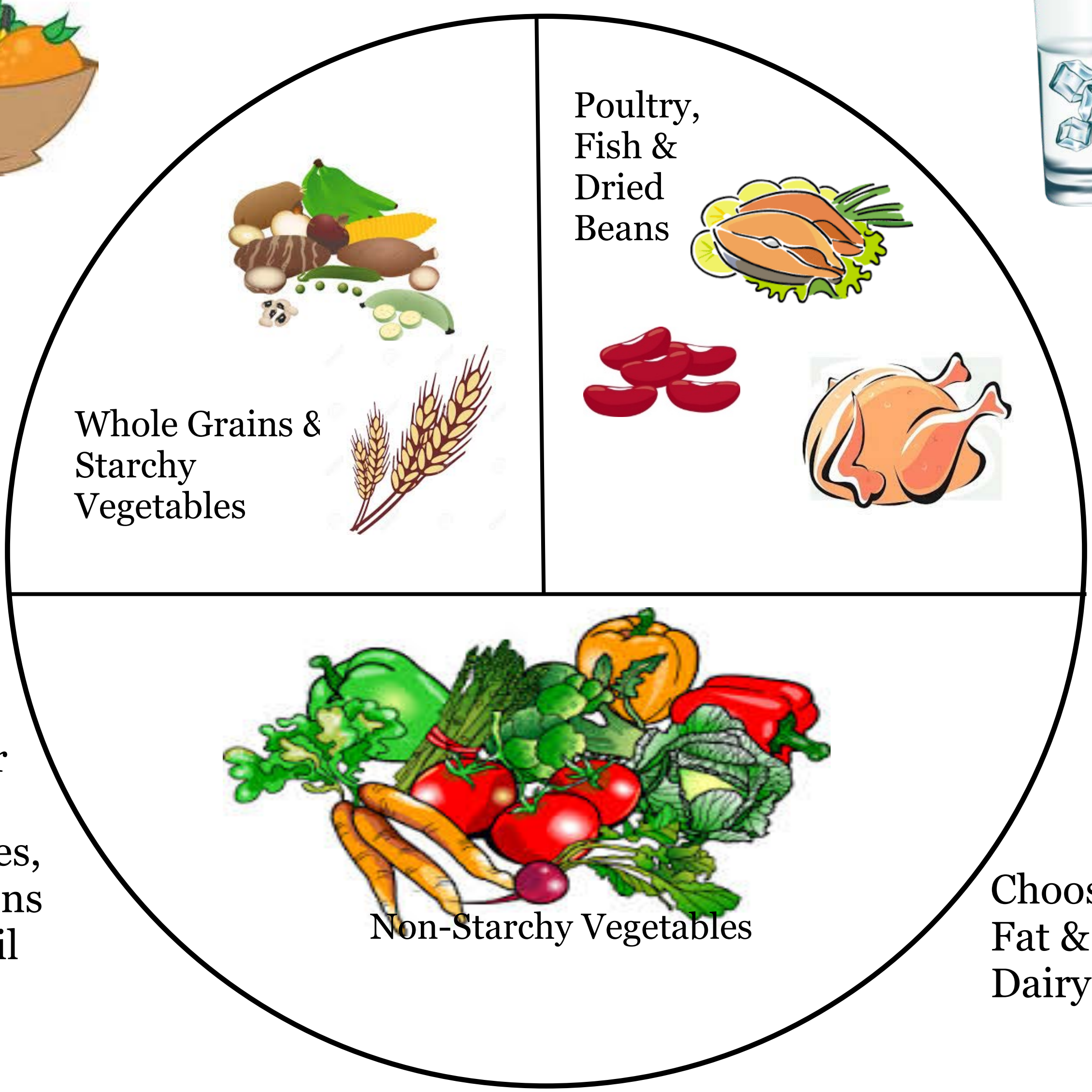

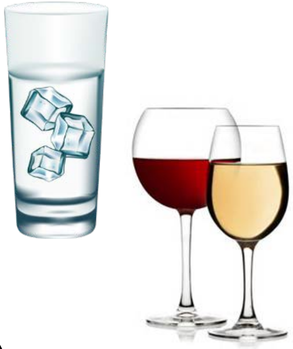

Drink water, tea or coffee with little or no sugar. May have 1-2 glasses of wine each day, but discuss with your medical team first.

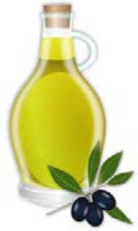

Flavor your food with herbs, spices, garlic, onions and olive oil

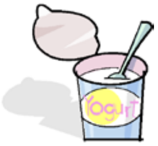

Choose Non-Fat & Low-Fat Dairy Products

Mediterranean Diet

Choosing a diet similar to one eaten by people living around the Mediterranean Sea may lower the risk of cardiovascular disease and have additional health benefits. The diet is mostly plant-based with high amounts of fresh vegetables, fruits, nuts, dried beans, olive oil, and fish. Follow these tips to eat the Mediterranean way!

Food/Food Group	**Recommended Intake***	Tips
Vegetables	4 or more servings each day (one portion each day should be raw vegetables)	A serving is 1 cup raw or ½ cup cooked vegetables. Eat a variety of colors and textures.
Fruits	3 or more servings each day	Make fruit your dessert
Grains 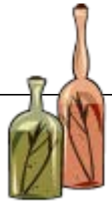	4 or more servings each day	Choose mostly whole grains.<br>1 serving = 1 slice bread or ½ cup cooked oatmeal
Fats/Oils	Olive Oil: 4 Tablespoons or more each day 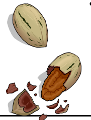	Choose extra virgin olive oil (EVOO) and use in salad dressings and cooking; choose avocado or natural peanut butter instead of butter or margarine
Dried Beans/Nuts/Seeds	Nuts/Seeds: 3 or more servings each week<br>Beans/Legumes: 3 or more servings each week	1 ounce or 1 serving = 23 almonds or 14 walnut halves; 1 serving of beans = ½ cup
Fish and Seafood	2-3 times each week 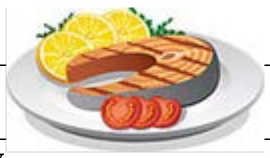	Choose salmon, sardines, and tuna which are rich in Omega-3 fatty acids
Herbs and Spices	Use daily	Season foods with herbs, garlic, onions and spices instead of salt
Yogurt/Cheese/Egg Poultry	Choose daily to weekly	Choose low-fat yogurt and cheeses; choose skinless chicken or turkey in place of red meat
Alcohol/Wine	Men: 1-2 glasses each day<br>Women: 1 glass each day	Always ask your medical team if alcohol is ok for you to consume.

***Serving sizes should be individualized to meet energy and nutrient needs.**

❖ Red meats, processed meats, and sweets should be limited

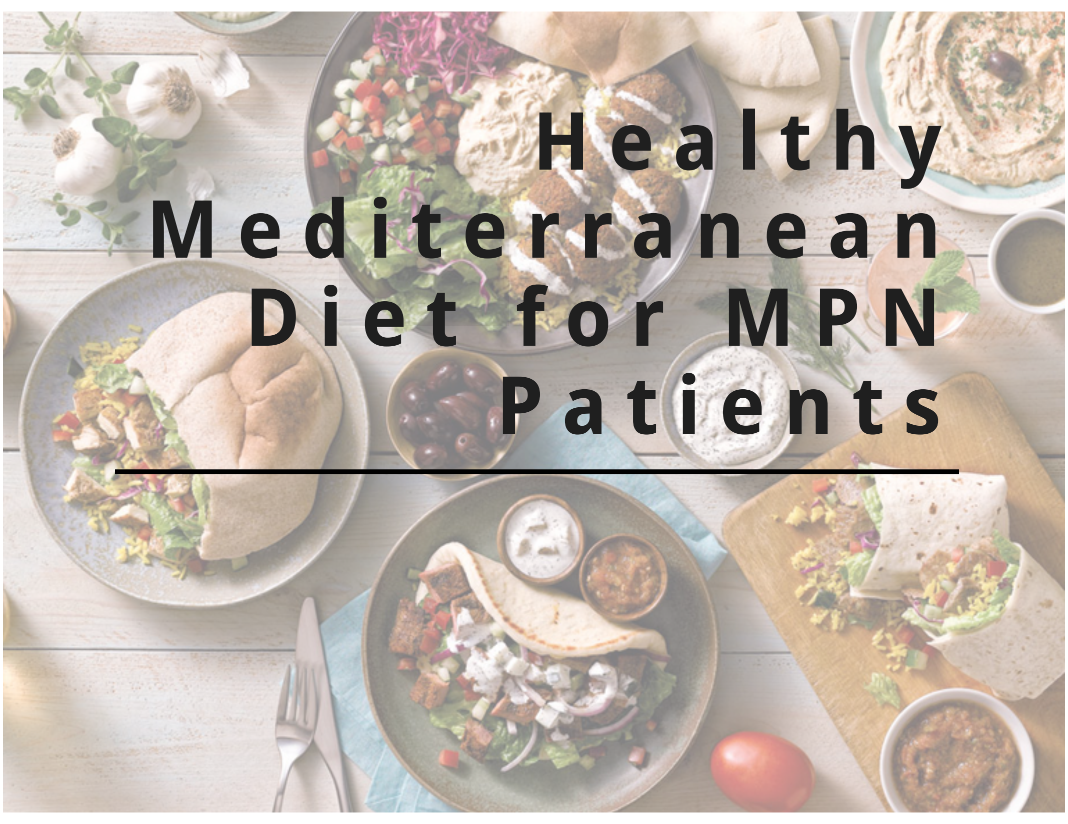

Healthy Mediterranean Diet for MPN Patients

Tip: Choose veggies over meat for lunch!

We are MPN

Week 3

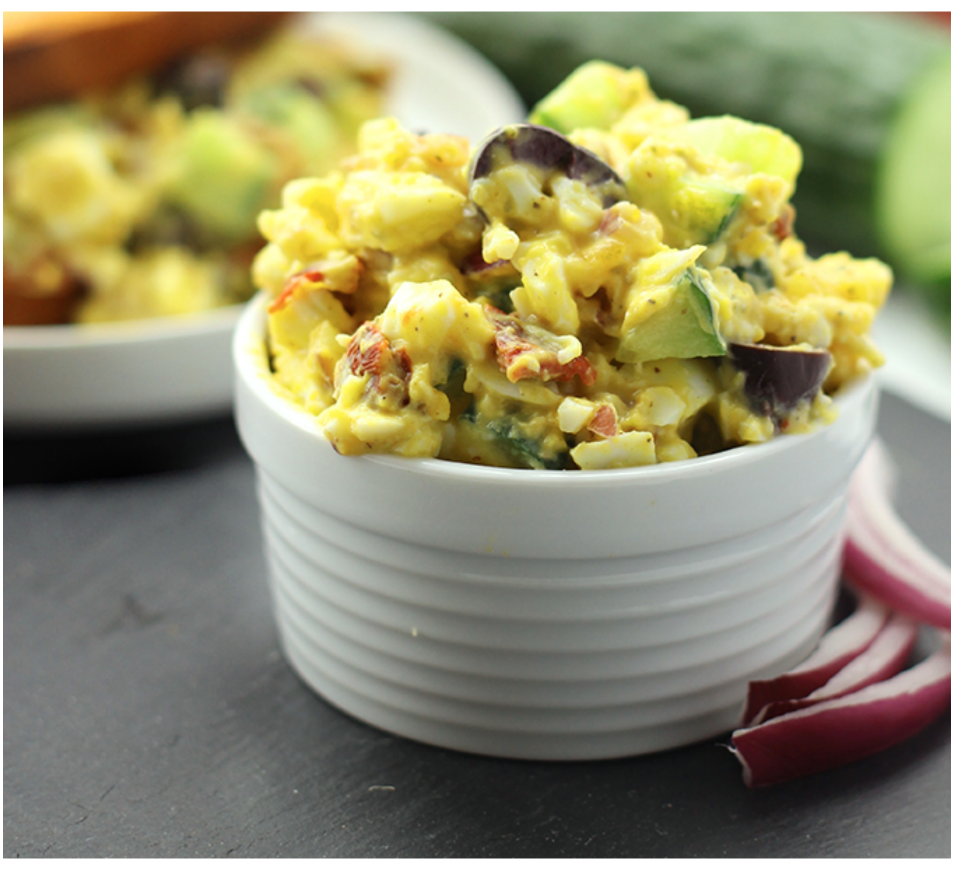

Egg Salad Recipe

Quick and easy lunch that lasts for up to a week!

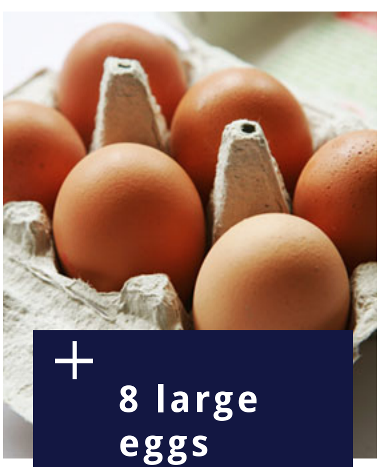

+ 8 large eggs

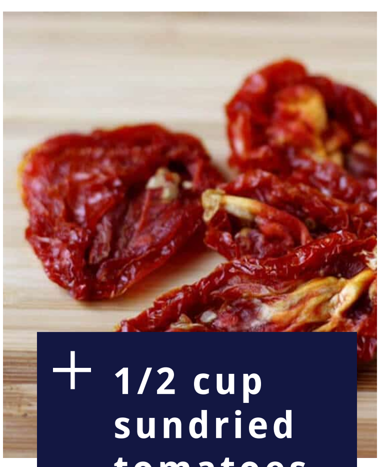

+ 1/2 cup sundried tomatoes

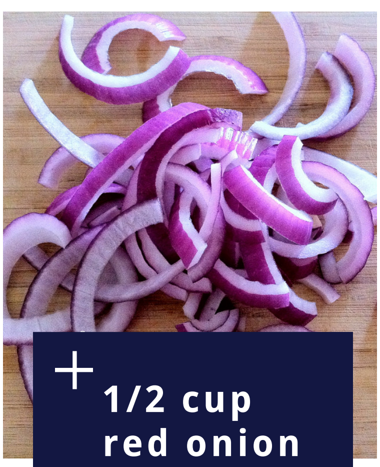

+ 1/2 cup red onion

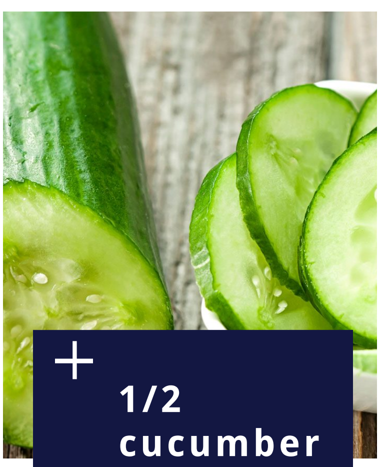

+ 1/2 cucumber

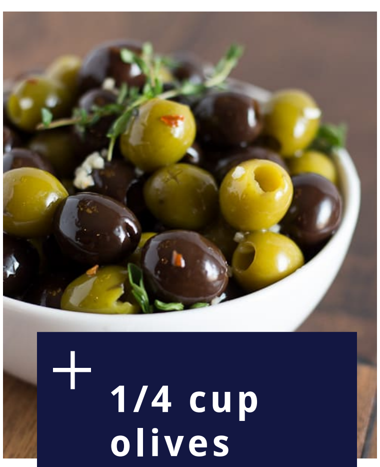

+ 1/4 cup olives

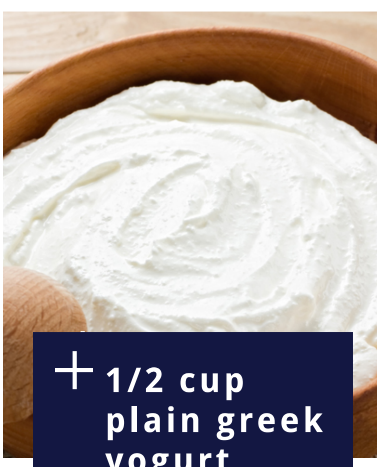

+ 1/2 cup plain greek yogurt

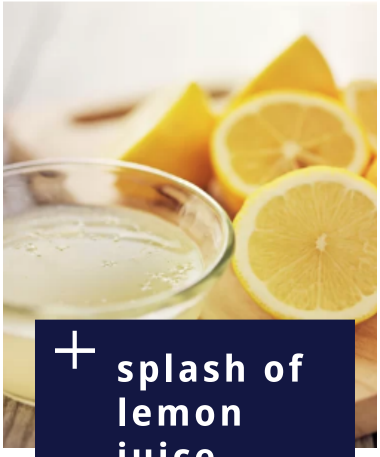

+ splash of lemon juice

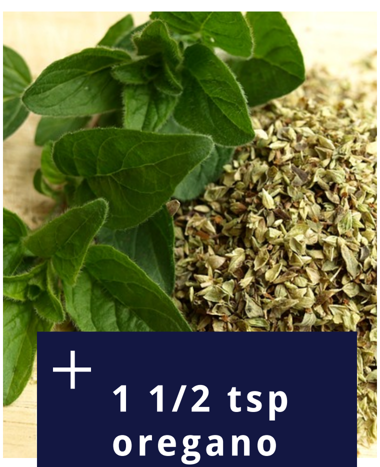

+ 1 1/2 tsp oregano

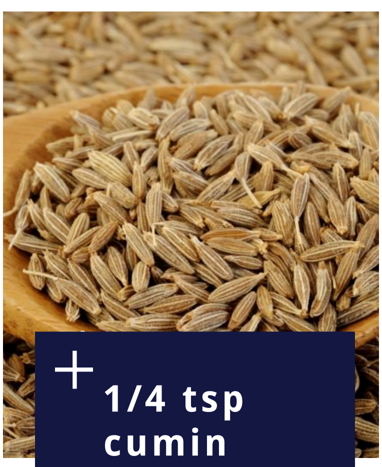

+ 1/4 tsp cumin

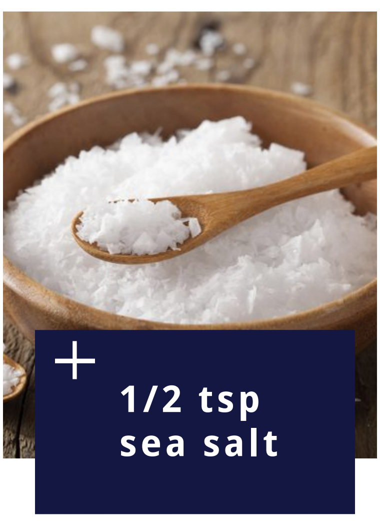

+ 1/2 tsp sea salt

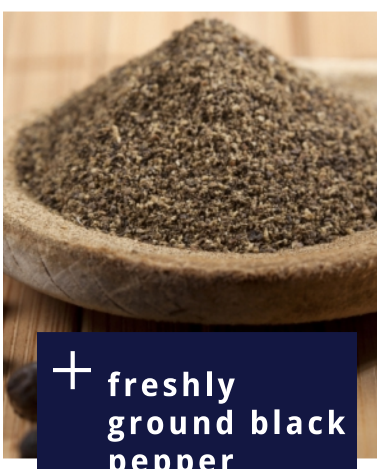

+ freshly ground black pepper

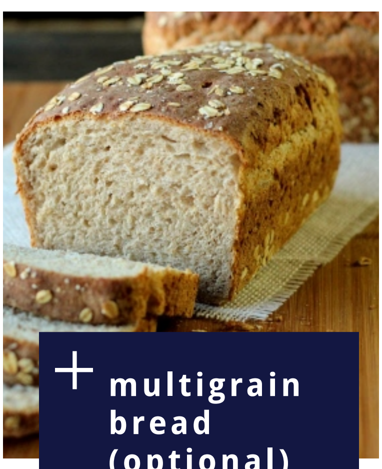

+ multigrain bread (optional)

Directions

1. Hard boil the eggs and chop them up.
2. Chop up the sundried tomatoes, red onion, cucumber, and olives.
3. Add sundried tomatoes, red onion, cucumber, and olives to chopped up eggs.
4. Stir in the Greek yogurt, lemon juice, and spices.
5. Store in fridge for up to a week.
6. Eat with multigrain bread (optional).

Recipe Credit: https://www.thehealthymaven.com/2015/03/mediterranean-egg-salad.html

Facts and Common Myths

Mediterranean Diet Basics

Q: Why is it called "Mediterranean?"

A: This way of eating is typical of the region surrounding the Mediterranean Sea, in countries like Spain, France, Italy, Egypt, Morocco, Syria, Malta, Tunisia, Turkey, Algeria, Albania, Greece, Israel, Croatia, Libya, and Lebanon.

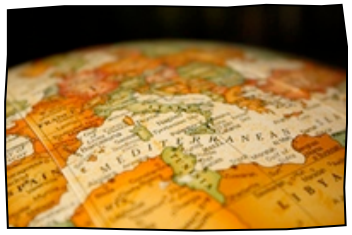

Q: Is it really a "diet" – will I be hungry all the time?

A: The Mediterranean diet (or Med Diet as it's often called) is more than a diet; it's a lifestyle approach to healthy eating. It features fruit, vegetables, fish, beans, nuts and whole grains as well as other ingredients such as olive oil and wine that have been shown to promote good health.

Q: How can I follow the Med Diet?

A: It's easy and filled with healthy foods that taste great. Just follow a few easy tips such as these:

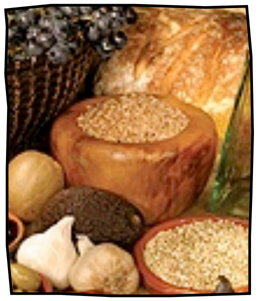

- Choose healthy fats like those found in olive oil, nuts, peanuts, avocados, and fish.
- Base every meal around fruits, vegetables, whole grains, beans, nuts, herbs and spices.
- Choose lean protein sources like fish, poultry, and beans more often than red meat.
- Enjoy yogurt and small portions of cheese daily.
- Drink wine in moderation (up to two glasses per day for men and one glass per day for women).

Q: Why should I follow the Mediterranean Diet?

A: Studies show that people who eat a Mediterranean Diet have lower rates of heart disease, certain cancers, diabetes, obesity, and Alzheimer's disease as well as lower blood pressure and cholesterol levels. Even better, following the Mediterranean Diet may help you live longer — so eat up!

Common Myths

"The Mediterranean Diet is just another fad diet."

Wrong! The Mediterranean Diet is a lifestyle; it's a sustainable way of eating; it's consistently found to promote good health AND decrease chronic disease risk.

"The Mediterranean Diet is a relatively new way of eating."

Leading nutrition scientists have been intensely studying the eating habits of Mediterranean people for more than 60 years. It all started when Ancel Keys, the famous researcher and father of the Mediterranean Diet, discovered, in the 1940s, that people who ate a Mediterranean-style diet had very low rates of heart disease and were living longer than people in Northern Europe.

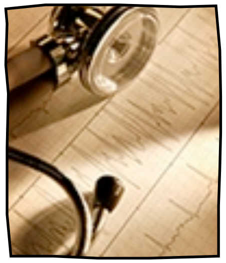

"The Med Diet consists of hard-to-get, foreign foods."

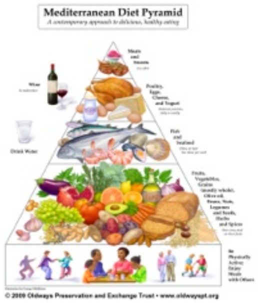

The Mediterranean Diet Pyramid is filled with foods most people eat every day, like produce, yogurt, milk, cheese, and seafood. The biggest difference between the Med Diet and the typical American diet is the frequency certain foods are eaten. Foods from the plant kingdom – fruits, vegetables, grains, beans, and nuts – are at the core, while foods like sweets and meats are eaten less often and in smaller amounts.

"Meats or sweets are not allowed in the Mediterranean Diet."

All foods fit in the healthy Mediterranean eating pattern. Moderation is key, but there's no reason to eliminate entire food groups or completely cut out your favorite foods.

"I can eat whatever I want on the Mediterranean Diet."

While it's true that all foods fit in the Mediterranean Diet, portion size and balance are still key in the Mediterranean Diet – and any other healthy, balanced eating pattern for that matter.

Copyright 2012 | Oldways | Mediterranean Foods Alliance | 266 Beacon St., Boston, MA 02116 | www.oldwayspt.org

Healthy Mediterranean Diet for

MPN Patients

Week 4: Mediterranean Diet Basics

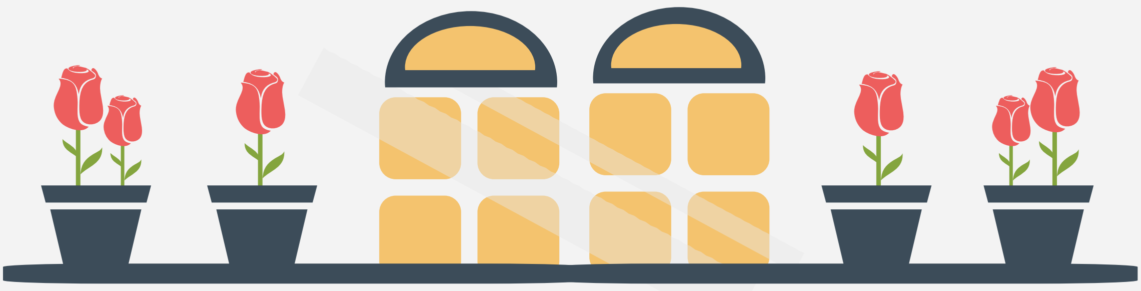

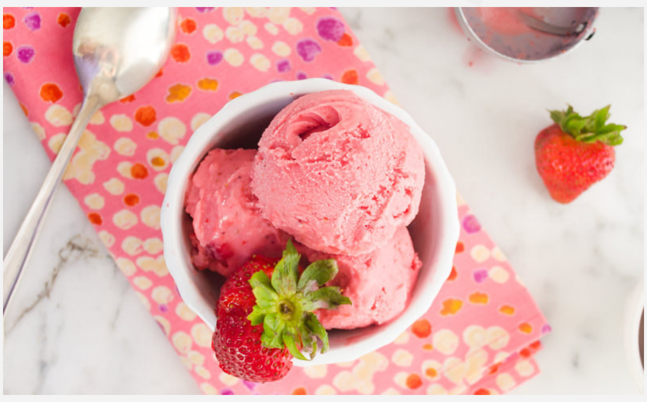

Recipe: Strawberry Greek Frozen Yogurt

You will need:

1 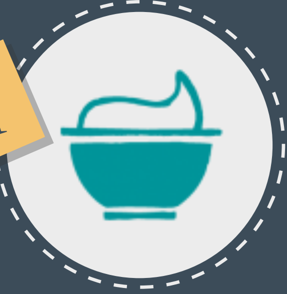

3 cups of plain Greek low-fat (2%) yogurt

2 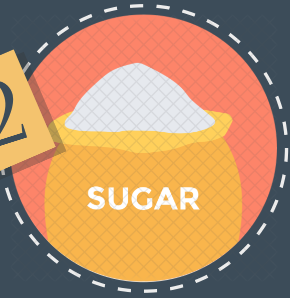

1 cup sugar

3 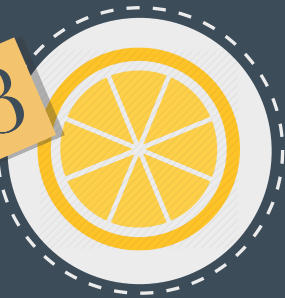

1/4 cup of freshly squeezed lemon juice

4 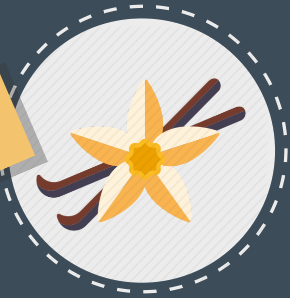

2 teaspoons of vanilla

5 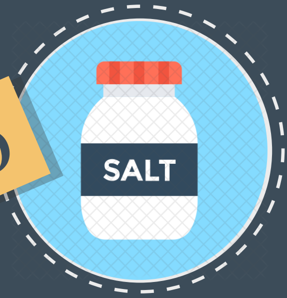

1/8 teaspoon of salt

6 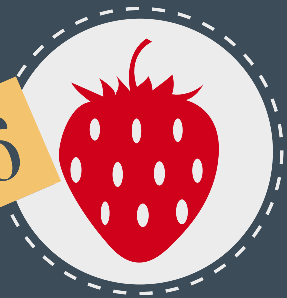

1 cup of sliced strawberries

Directions

1. Combine the yogurt, sugar, lemon juice, vanilla, and salt in a medium sized bowl. Whisk it together until it has a smooth consistency.
2. Freeze the yogurt mixture in a 1 1/2 - 2 quart ice cream maker (following the machine's instructions. Add in the sliced strawberries at the last minute and transfer the frozen yogurt to an airtight container.
3. Let it freeze for 2-4 hours before serving.

**makes 16 servings

Mediterranean Diet All-Stars

Meet just a few of the many nutrition powerhouses that form the foundation of the Mediterranean Diet.

Avocados

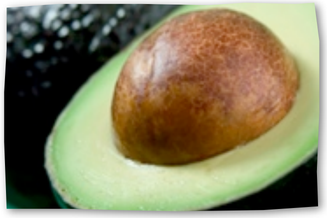

High in fiber, and filled with healthy monounsaturated fat and vitamin E, avocados are available all year. Add them to salads, use in dips, or simply eat out of the shell with a spoon.

Fish

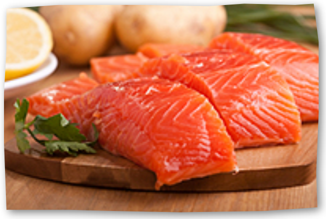

Eat fish, which contain healthy fats, twice a week. Salmon, sardines, and mackerel are great sources of heart healthy omega-3 fatty acids.

Tomatoes

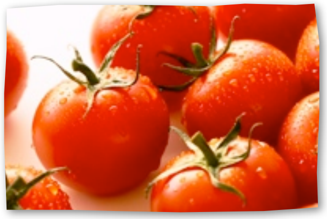

A source of vitamin C and lycopene, a potent antioxidant, tomatoes stimulate immune function and help fight chronic diseases.

Yogurt

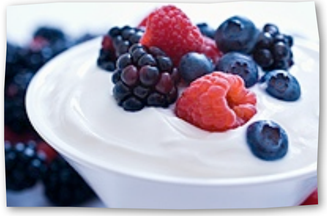

A protein powerhouse, yogurt contains calcium to protect and strengthen bones and also has beneficial bacteria that are important for digestive health. Look for Greek yogurt, which delivers twice the protein of regular yogurt, plus a rich, tangy taste.

Beans

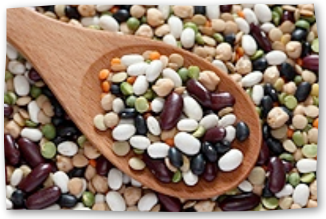

A great source of protein and fiber, swap beans for meat to make one or two meatless meals per week. If you use canned beans, rinse them well to remove some of the sodium.

Nuts, Peanuts, Seeds

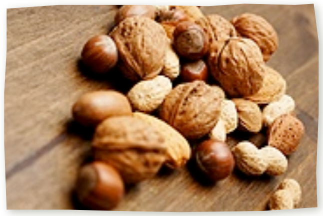

Packed with protein, fiber and heart-healthy fats, a handful of nuts makes a good snack. Or, add a small amount of sesame or sunflower seeds to salads or toss them with roasted vegetables.

Wine

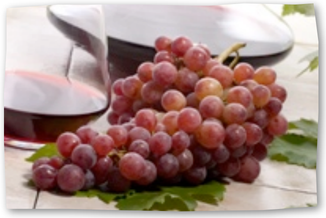

Wine contains powerful antioxidants that come from grape skins and seeds and has been shown to reduce the risk of most diseases of aging. Enjoy up to one glass a day for women and two for men to help prevent strokes. If you're not a wine drinker, have a glass of 100% grape juice.

Whole Grains

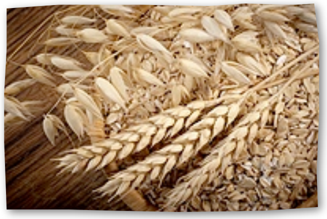

Packed with nutrients, fiber and protein, whole grains contain "good" carbs and are an important choice for healthy eating. Learn to cook popular Mediterranean whole grains such as barley, brown rice, bulgur, whole wheat couscous, and farro, for salads and side dishes.

Copyright 2012 | Oldways | Mediterranean Foods Alliance | 266 Beacon St., Boston, MA 02116 | www.oldwayspt.org

Avocados are naturally FULL of nutrition, so there's no need for special labels. They're full of flavor; think of all the meals you can healthify with avocado!

Week 5

AVOCADOS

HEALTH TIP!

Avocados are one of the only fruits that contain heart healthy monounsaturated fat (the good-for-you fat) that helps boost good (HDL) cholesterol and lowers bad (LDL) cholesterol.

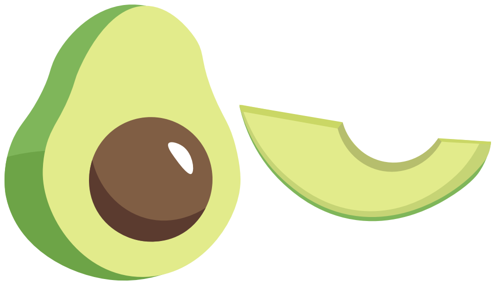

Anti-aging tool for your skin!

- mbgfood

Recipe:

Avocado Toast with Lemon and Kale

You will need:

- 1 cup shredded kale (no stems)
- 1/2 lemon
- 1 teaspoon olive oil
- 4 slices multigrain bread, 4 oz total
- kosher salt and black pepper
- 4 ounces avocado (1 small haas)
- 1/8 teaspoon cumin
- 4 thin slices radish
- 1 tea spoon chia seeds

Directions:

1. In a bowl combine the kale, olive oil, juice of 1/4 lemon and 1/8 teaspoon salt
2. Massage with your hands for about 1 minute, until the kale softens
3. Slice the avocado in half, reserving 1/2 of one avocado for thin slice. Scoop the rest into a small bowl and mash gently with a fork. Season with 1/8 tbs kosher salt, black pepper and juice from 1/4 of a lemon.
4. Toast the slices of whole wheat bread
5. Spread the avocado mash across the toasted bread , top with slices and sprinkle with cumin, salt and pepper (more lemon juice if desired)
6. Top each with the massaged kale, radish and chia, finish with pinch salt and black pepper to taste

All images and text @Gina Homolka for Skinnytaste

Fact #2

Tips for saving leftover avocado

- To store unused 1/2 of avocado, wrap tightly with plastic wrap and place in refrigerator.
- Leave the pit in and get the wrap as close to the surface of the fruit as possible.
- If browning occurs, gently scrape the exposed flesh with a butter knife.

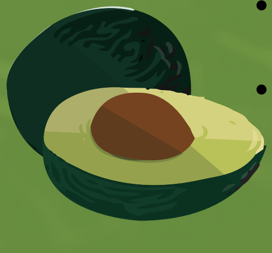

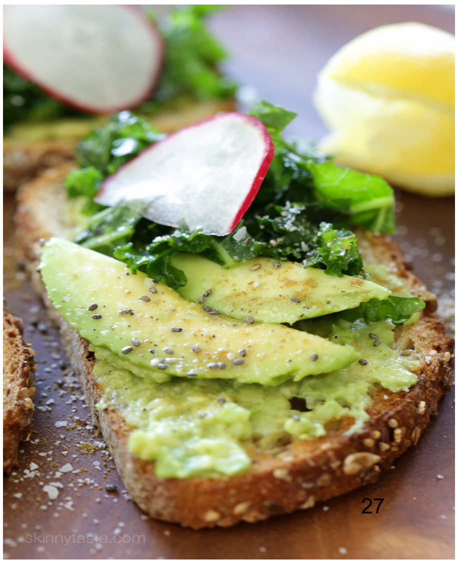

Make It YOUR Diet

To help you bring the gold-standard Mediterranean Diet into your daily life, try these tips to get started.

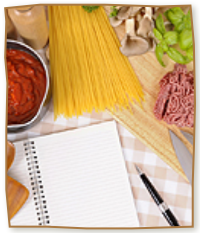

- Stock your pantry with versatile Med ingredients so you always have foods like olive oil, canned tomatoes, tuna, rice, pasta, and other whole grains on hand. It's amazing how many easy Mediterranean meals you can make from a well-stocked pantry, when there's no time to shop.
- Use a blender to make Med-style smoothies for breakfast or as fuel for afternoon snacks, by combining yogurt with your favorite fruit. Frozen fruits (including berries) are especially good – they eliminate the need for any ice.

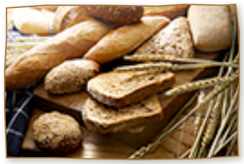

- When it's sandwich time, match better breads with better spreads. Start with crusty whole grain breads and rolls or pita pockets – tastier and healthier than standard white bread – then spread with hummus, mustard, pesto or another flavorful Med spread. Add foods such as tuna, sliced turkey or chicken, lettuce, sprouts, shredded raw carrots, thin slices of cheese, and sliced apples.
- Keep pre-cooked frozen shrimp in your home freezer. Shrimp cooks quickly, making it an easy addition to one-pot sautés and pasta dishes. Canned salmon is a great choice, too.

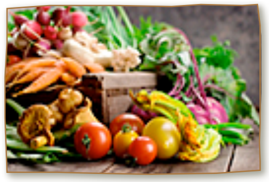

- Use meat as a flavoring instead of a main component in a meal. Add small strips of sirloin to a sauté that features lots of vegetables, or add a small amount of diced prosciutto to a dish of pasta.
- Eat a vegetarian meal one night each week. When that feels comfortable, try two nights per week.

Keep Snacks Simple

- Top pita bread with a slice of tomato and a few tablespoons of grated cheese and broil for a minute to create a healthy mini-pizza.
- Marinate olives in olive oil, lemon zest, coriander seeds and cumin seeds and enjoy as a tasty snack.
- Enjoy popcorn air-popped and tossed with a bit of olive oil and Parmesan cheese.
- Focus on fruit. Eat an apple or an orange, or have a peach with ricotta or cottage cheese, or spread a few apple slices with peanut butter.
- Fill celery stalks with hummus or different nut butters. Or, keep string cheese on hand and enjoy a piece between meals.

Brown Bag it To Work

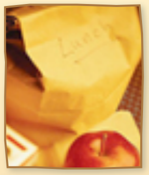

Fill a whole-grain pita pouch with Greek salad and put the dressing in a separate container; add the dressing just before eating to keep the sandwich from getting soggy.

Take a thermos of soup or vegetable stew to work. Toss in some leftover whole grains before you screw on the lid, to make your soup even healthier.

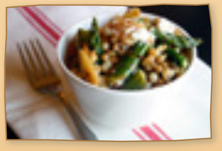

Transform leftover brown rice, quinoa, and other whole grains into lunch by mixing them with chopped raw vegetables or beans and adding a little salad dressing.

Keep whole grain bread in the freezer and make a sandwich using frozen bread and hummus, sprouts, leafy greens, sliced peppers, turkey, chicken, or smoked salmon. By lunchtime the thawed bread will taste fresh.

Pack a container of Greek yogurt, which has twice the protein of regular yogurt, and combine it with chopped fruit and a sprinkling of chopped nuts.

Copyright 2012 | Oldways | Mediterranean Foods Alliance | 266 Beacon St., Boston, MA 02116 | www.oldwayspt.org

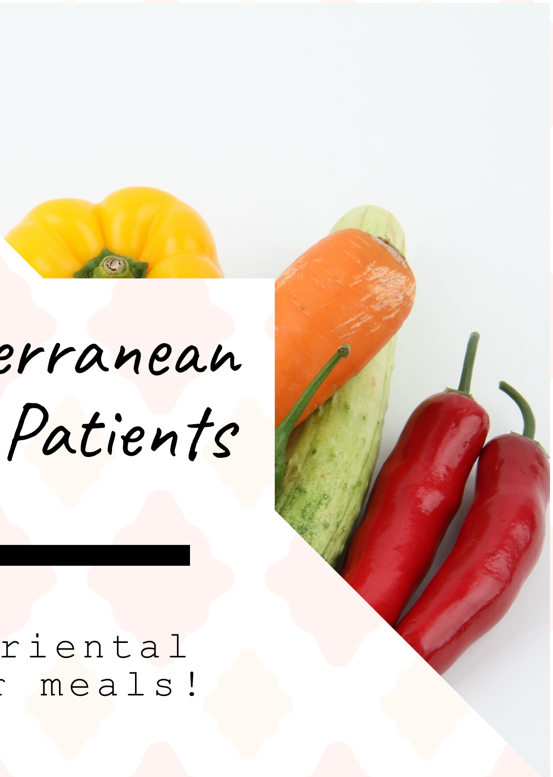

Healthy Mediterranean Diet for MPN Patients

Week 6: Add oriental spices to your meals!

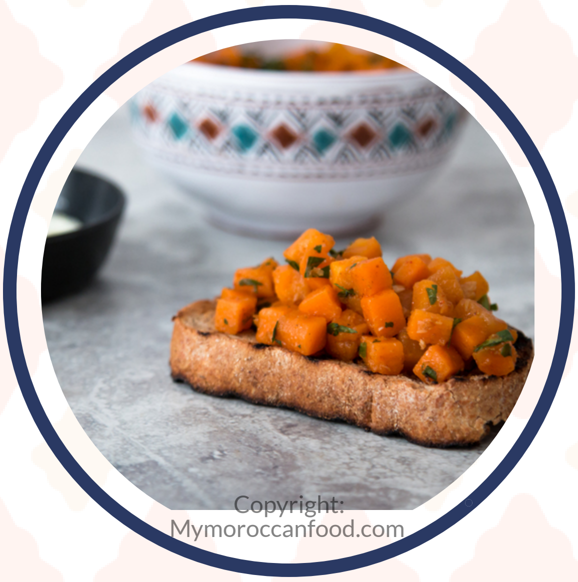

Copyright: Mymoroccanfood.com

Moroccan carrot salad recipe

Ingredients (serves 4):

- 500 gr (4 cups)of carrots, peeled and sliced or chopped
- 2 garlic cloves, unpeeled
- 3 tablespoons finely chopped coriander
- 3 tablespoons finely chopped parlsey
- 1 teaspoon ground paprika
- 1 teaspoon ground cumin
- 2 tablespoons white wine vinegar
- 2 tablespoons olive oil
- Salt to season

• In a medium sized pan, bring water to the boil, add the carrots and the garlic cloves. Reduce the heat to medium and gently cook the carrots until lightly crisp and tender, about 10 to 20 minutes depending on the size of your sliced/chopped carrots.

• Drain, rinse under cold water and drain again. Set aside.

• Peel and finely chop the garlic cloves.

• In a small bowl, transfer the garlic, chopped herbs, paprika, cumin, vinegar, olive oil, and salt. Stir until smooth to make the dressing.

• Gently combine the cooked carrots with the dressing. Serve warm or cold.

Did you know?

In Morocco, a lot of different herbs and spices are frequently used in the kitchen: cilantro, parsley, cumin, ginger, turmeric, cinnamon and saffron. Saffron threads come from a purple flower, *Crocus sativus*, and are the most expensive spice in the world!

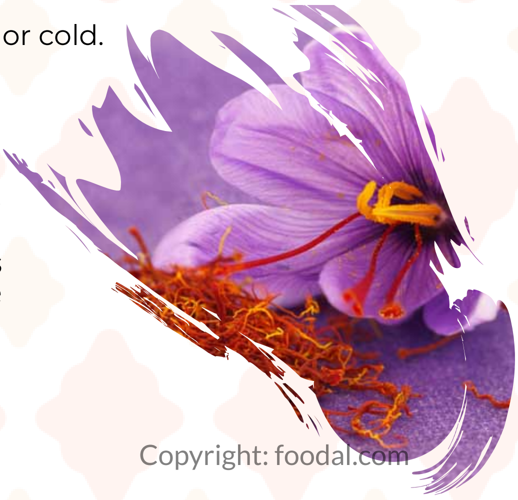

Copyright: foodal.com

We are MPN

The Med Diet and Your Family

The Mediterranean Diet is all about delicious flavors, textures and colors, with something for everyone—even finicky eaters! Try these tips for introducing the Med Diet to your family.

❖ **Exploring the Med diet** gives the whole family an opportunity to discover new tastes together. Introduce a small taste of a new food each week to encourage variety.

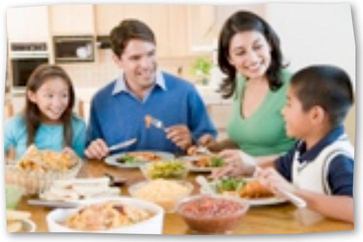

Be patient and keep in mind that kids' palates change over time. It can take up to 20 tries over weeks and months for them to accept a different flavor or texture. And be a role model by eating new foods yourself.

❖ **Encourage your kids to play with their food!** When they need an after-school snack or before supper when everyone is starving, get into the habit of setting out small bowls of Mediterranean Diet favorites: tzatziki, baba ghannouj, spicy muhammara, and different flavors of hummus.

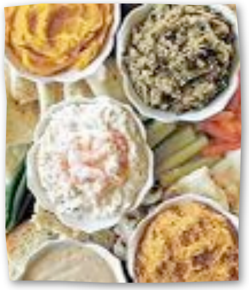

Provide an ever changing variety of fresh, raw vegetables cut into pieces small enough for dipping: baby carrots, celery sticks, sliced cucumbers, sliced red, green, yellow, and orange peppers, snow peas, sliced fennel, and zucchini strips and let them dip away. Try whole grain pita for dipping too!

❖ **Add vegetables to the kinds of foods your kids already like.** If pancakes are popular, add some grated carrots, or shredded zucchini to the batter. Toss frozen peas with hot pasta, camouflage extra veggies in a zesty spaghetti sauce, or add diced sautéed onions or peppers to scrambled eggs.

❖ **Make your own family-favorite trail mix.** In a large bowl, combine peanuts, chopped walnuts, raisins or other dried fruit, some whole grain cereal and a few small pieces of chocolate if you wish. Package it in "snack-size" zip-lock bags to have on hand for car trips and lunch boxes.

❖ **Luscious fruits at every meal** are a key part of the Mediterranean Diet, so keep apples, clementines, oranges, grapes, pears, melon, peaches, dates, strawberries and other fruits in plain sight.

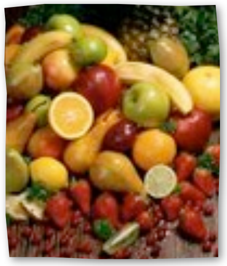

Encourage the whole family to eat fresh fruit rather than drink fruit juice for a better source of fiber, often lacking in our diets. Make healthy parfaits for dessert by layering Greek yogurt and sliced fresh fruit in tall glasses. Use frozen berries to make great smoothies.

❖ **Almost everything tastes better with olive oil.** Kids who won't eat a steamed carrot may love them roasted (or raw!) And you may succeed in getting everyone to eat sweet potatoes if you serve them as oven-baked fries rather than mashed. Or, try making kale chips instead of steamed kale, for a whole different take on greens.

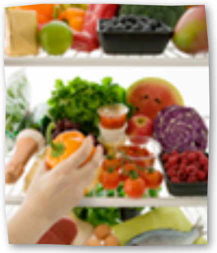

❖ **Create Med-style "variety plates"** to help your kids enjoy a wide range of flavors and to serve up a well-balanced meal. Put small helpings of six or eight different foods on a colorful plate or small platter, relying on leftovers and items you have on hand.

For example: a few slices of cold turkey, a few baby carrots, a spoonful of hummus, several cubes of cheese, a helping of pasta or a whole grain roll, a small serving of lettuce with a favorite dressing, apple slices, a few olives, and several pickles. Kids usually enjoy such a selection.

❖ **Get the family involved in preparing meals.** Let little kids wash fruits and vegetables at the sink; ask older kids to chop the vegetables, toss the pasta, dress the salad and set the table. Children who learn basic kitchen skills appreciate it later in life and have a legacy to pass along to their children.

Mediterranean Foods Alliance

Copyright 2012 | Oldways | Mediterranean Foods Alliance | 266 Beacon St., Boston, MA 02116 | www.oldwayspt.org

Oldways Health Through Heritage

Healthy Mediterranean Diet for MPN Patients

Week 7

NUTS

Health Tip!

Previous studies have suggested that eating nuts may have antioxidant and anti-inflammatory events and can improve blood lipids and endothelial function and prevent weight gain.

DARK CHOCOLATE NUT CLUSTERS WITH SEA SALT

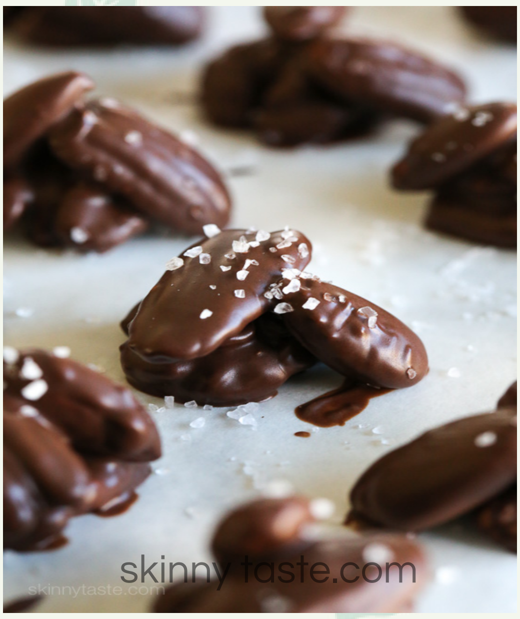

Recipe

Ingredients:

- 20 almonds (total weight 20 grams)
- 20 pecan halves (total weight 20 grams)
- 20 walnut halves (total weight 60 grams)
- 1 package Ghirardelli Dark Chocolate Melting wafers*
- Sea salt

Recipe

Directions:

1. Place chocolate wafers in a medium microwave safe bowl. Melt in 30 second increments, stirring until melted until 11/2 minutes total.

2. Working quickly, dip a walnut into the melted chocolate with a fork and shake the excess off. Transfer to a piece of wax paper. Do the same with the pecan, then lay the pecan on top of the walnut. Repeat with the almond, finishing the cluster. Repeat with the remaining nuts.

3. Finish each with a pinch of sea salt, to do this you will want to sprinkle it on top once they are almost dry so it doesn't absorb, but not to dry or it won't stick. Enjoy!

Tip

Eating nuts several times a week may play a role in reducing cardiovascular risk.

*Calculated with 80 grams Ghirardelli Dark Chocolate Melting Wafers for 20.

All images and text @Gina Homolka for Skinnytaste

Set Up Your Kitchen

Organize your kitchen and pantry shelves to have the important staples of the Mediterranean Diet within easy reach. Keep a variety of choices from the following key ingredients on hand so you can always make a healthy meal:

In The Pantry

- ✓ **Beans:** Chickpeas, cannellini, fava, and kidney beans; lentils.
- ✓ **Breads:** Bread crumbs, foccaccia, lavash, pita, and other breads (mostly whole grain).
- ✓ **Canned Seafood**: Anchovies, clams, salmon, sardines, tuna.
- ✓ **Capers**: Once opened, they will keep in the refrigerator for up to six months.
- ✓ **Cereals**: Oatmeal, plus other hot or cold cereals. The best choices list the first ingredient as whole grain, provide at least 3 grams of fiber and no more than 8 grams of sugar per serving.
- ✓ **Crackers:** Look for labels that list a whole grain ingredient first, and that provide 2–3 grams of fiber per serving.
- ✓ **Dried Fruit:** Apricots, blueberries, cherries, cranberries, figs, raisins, prunes.
- ✓ **Garlic:** Keep a head or two within easy reach. Or, buy peeled garlic cloves and store in the refrigerator.
- ✓ **Grains:** Bulgur, couscous, farro, millet, oats, polenta, rice, quinoa.
- ✓ **Herbs and Spices:** Basil, bay leaves, black pepper, cinnamon, cloves, coriander, crushed red pepper, cumin, curry powder, dill, garlic powder, ginger, oregano, paprika, rosemary, saffron, sage, thyme, turmeric, or blends like Italian seasoning.
- ✓ **Honey**
- ✓ **Nuts:** Almonds, hazelnuts, peanuts, pine nuts, pistachios, walnuts
- ✓ **Oil:** Extra-virgin olive oil, grapeseed oil, peanut oil
- ✓ **Olives:** (all types)
- ✓ **Pastas:** (all types, including whole grain)
- ✓ **Potatoes**
- ✓ **Red Wine**
- ✓ **Salt:** Kosher, sea and iodized
- ✓ **Seeds:** Fennel, flax, sesame, sunflower
- ✓ **Tomatoes:** Canned, paste, sauce, sun-dried
- ✓ **Vinegars:** Balsamic, champagne, cider, fig, red wine, white wine

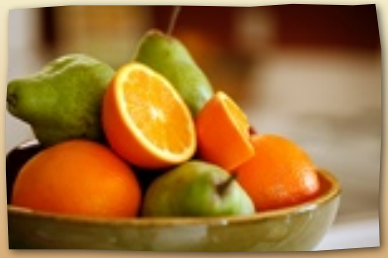

On The Counter

- ✓ **Fresh Fruit** ~ Avocados, apricots, cherries, clementines, figs, grapefruit, lemons, limes, oranges, melons, nectarines, dates, bananas, plums, apples, peaches, pears, pomegranates, and/or tangerines. These fruits keep best at room temperature.
- ✓ **Tomatoes** ~ Store fresh tomatoes at room temperature.
- ✓ **Olive oil** ~ Keep an olive oil dispenser near your stovetop, and store the rest in a cool, dark place.

In The Refrigerator

Cheese
(soft and hard varieties)
Eggs
Fruit
(berries, grapes)
Poultry
Hummus and other Med dips
Milk
Mustard
Pesto
Pickles
Tapenade
Vegetables
(a variety, plus salad greens)
White and Sparkling Wine
Yogurt

In The Freezer

Frozen Fruit
Frozen Poultry and Meat
Frozen Seafood
Frozen Vegetables
Frozen Chicken Stock
Sorbet and Gelato

Mediterranean Foods Alliance
Copyright 2012 | Oldways | Mediterranean Foods Alliance | 266 Beacon St., Boston, MA 02116 | www.oldwayspt.org
Oldways Health Through Heritage

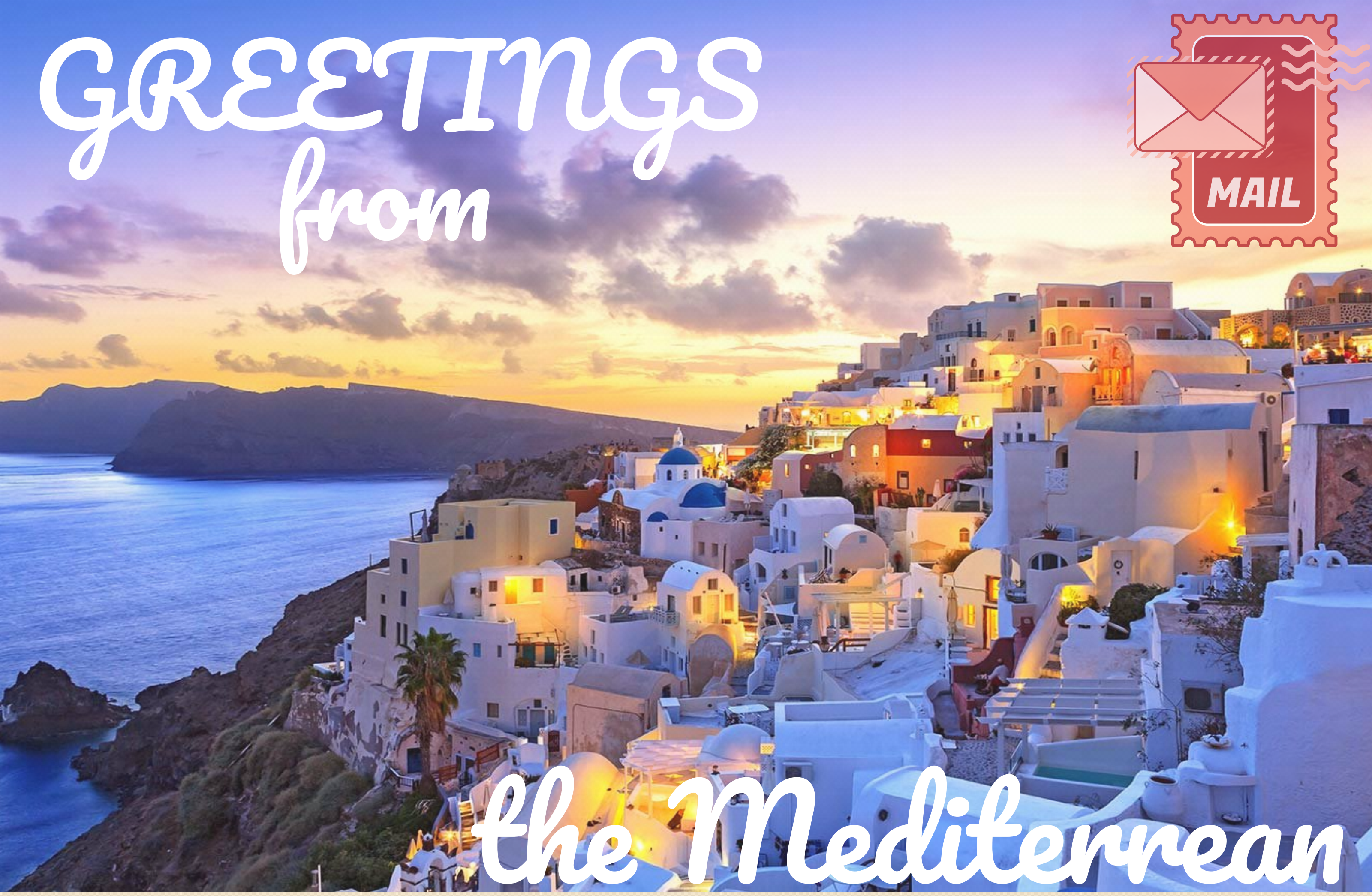

GREETINGS from the Mediterranean

Recipe/Images of @thekitchn.com and Getty Images

Postcard

Ingredients:

MINT TZATZIKI:

-1/2 cup grated cucumber
-1 tsbp diced mint
-3/4 plain nonfat yogurt
-2 teaspoons lemon juice
-1/4 teaspoon salt
-2 minced garlic cloves

LAMB PITAS:

-4 wheat pita bread rounds
-2 cups chopped/shredded lettuce
-1.5 cups of roasted leg of lamb
-3/4 cup chopped tomato
-1/2 cup diced red onion

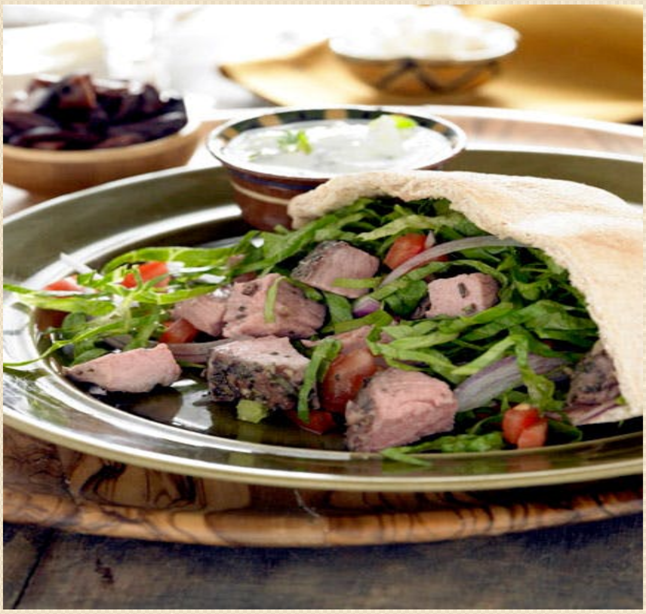

Stir together the ingredients listed under "MINT TZATZIKI" into a small bowl. Grab another bowl and mix all ingredients listed under "LAMB PITAS. " Cut open your pita bread rounds in half and fill with mixed ingredients from your two bowls.

Healthy New Habits

As you adopt the Mediterranean Diet, you'll open your tastebuds to a whole new world of flavors, while improving your health.

Avocados

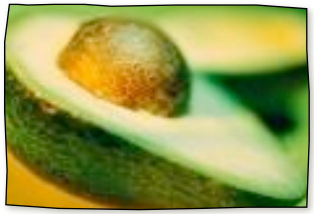

- Try mashed avocado instead of butter, jelly or cream cheese on bagels or toast.
- Enjoy guacamole instead of sour-cream dip.

Beans

- Add beans to chilies and casseroles, or use half ground turkey and half beans instead of ground beef.
- Puree cooked beans and use them as the base of healthy dips.
- Combine hummus with herbs and use as a sandwich spread instead of butter or mayo.

Fruit

- Eat a bowl of fresh berries and yogurt, instead of ice cream, or reach for grapes, oranges, or melon chunks instead of cookies.
- Reduce the oil in muffins and quick breads by half and substitute unsweetened applesauce or mashed banana to make up the difference.
- Serve a sandwich with sliced apples or pears on the side, instead of chips.
- End a meal with sweet, fresh fruit.

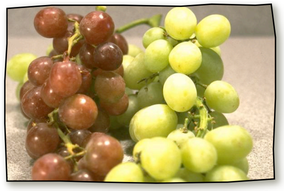

Herbs and Spices

- Use fresh or dried herbs and spices to add flavor to grain dishes, soups, dressings and sauces. You'll use less salt.
- Reduce sugar by about half in baked goods and add cinnamon, cloves, ginger and nutmeg to enhance the taste.

Olive Oil

- Dip bread into olive oil rather than spreading it with butter, or use olive oil instead of butter on cooked veggies.
- Toss popcorn with olive oil and a sprinkling of Parmesan cheese or herbs instead of butter and salt.
- Substitute olive oil for butter in baking for lighter-tasting breads and cakes. The chart at right shows how to make the switch.

Whole Grains

- Stuff peppers with cooked whole grains instead of meat.
- Cook brown rice, quinoa, whole wheat couscous, or other whole grains instead of white rice.
- Try whole wheat flour in place of half the white flour in recipes for cookies, muffins, quick breads, and pancakes.
- Use whole grain pasta instead of enriched pasta to triple the amount of fiber and reduce the number of calories.

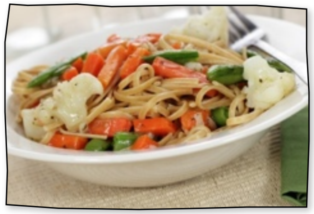

Yogurt

- Use Greek yogurt instead of mayonnaise in potato and pasta salads, or use 2/3 yogurt to 1/3 mayonnaise.
- Use Greek yogurt in place of sour cream in baking, or instead of cream in a dressing.
- Fruit-flavored yogurt can contain up to 5 teaspoons of sugar per serving. Buy plain yogurt instead, and flavor it with a teaspoon of jam or maple syrup, or your favorite fresh fruit.

Baking Substitutions

Butter	Olive Oil
1 tsp	3/4 tsp
2 tsp	1 1/2 tsp
1 Tbsp	2 1/4 tsp
2 Tbsp	1 1/2 Tbsp
1/4 cup	3 Tbsp
1/3 cup	1/4 cup
1/2 cup	1/4 cup +1 Tbsp
2/3 cup	1/2 cup
3/4 cup	1/2 cup + 1 Tbsp
1 cup	3/4 cup

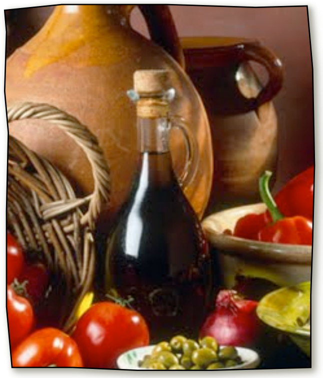

Mediterranean Foods Alliance

Copyright 2012 | Oldways | Mediterranean Foods Alliance | 266 Beacon St., Boston, MA 02116 | www.oldwayspt.org

Oldways Health Through Heritage

Sample Menu

Breakfast

1 cup Greek yogurt with ¾ cup berries or fresh fruit and ¼ cup walnuts
1 slice whole wheat toast with ¼ cup mashed avocado or 2 teaspoons natural nut butter
Coffee or tea

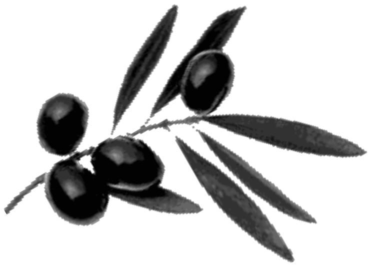

Lunch

1 cup lentil or minestrone soup
1 whole wheat pita
2 Tablespoon hummus
1/2 cup tomatoes, 1/2 cup cucumber with 2 Tablespoons olive oil, balsamic vinegar, and basil
Water with lemon wedge
1 apple, peach, or orange

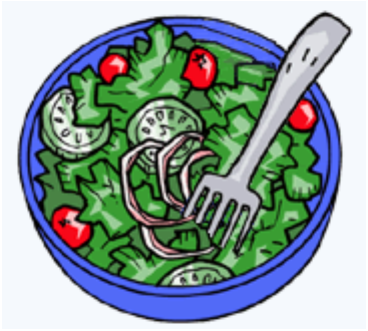

Snack

1 ounce low-fat mozzarella cheese and 15 grapes

Dinner

3-4 ounces broiled fish brushed with olive oil, seasoned with lemon and dill
1 cup brown rice, cooked
1 cup steamed carrots
1-2 cups baby spinach and arugula salad with 2 Tablespoons olive oil vinaigrette dressing
Decaf green tea or 5 ounce wine (if cleared by medical team)

Snack

1 ounce dark chocolate

Nutrition Information: 2200 calories (8% saturated fat, 11% polyunsaturated fat, 19% Monounsaturated fat), 43 grams fiber. 4 Tablespoons olive oil each day provides 480 of 2200 calories.

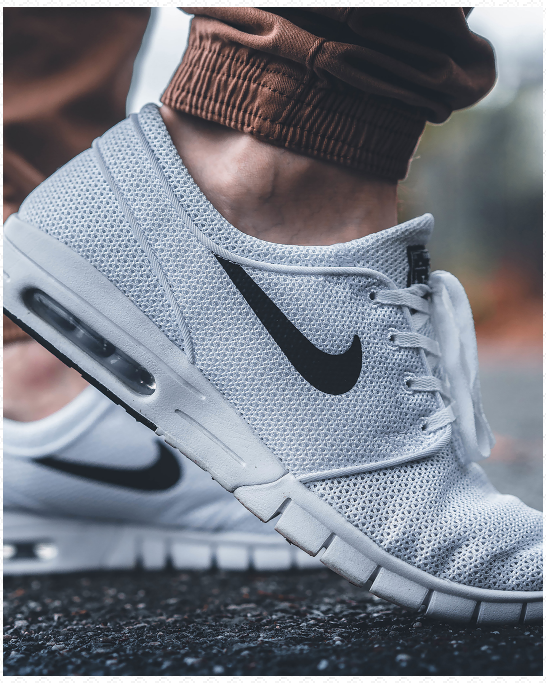

2015-2020

Physical Activity Guidelines for Americans

In addition to consuming a healthy eating pattern, regular physical activity is one of the most important things Americans can do to improve their health.

18 to 64 years

Adults should do at least 150 minutes a week of moderate-intensity, or 75 minutes a week of vigorous-intensity aerobic physical activity.

Aerobic activity should be performed in episodes of at least 10 minutes, and preferably, it should be spread throughout the week.

Adults should also include muscle-strengthening activities that involve all major muscle groups on 2 or more days a week.

For additional and more extensive health benefits...

adults should increase their aerobic physical activity to 300 minutes a week of moderate-intensity, or 150 minutes a week of vigorous-intensity aerobic physical activity.

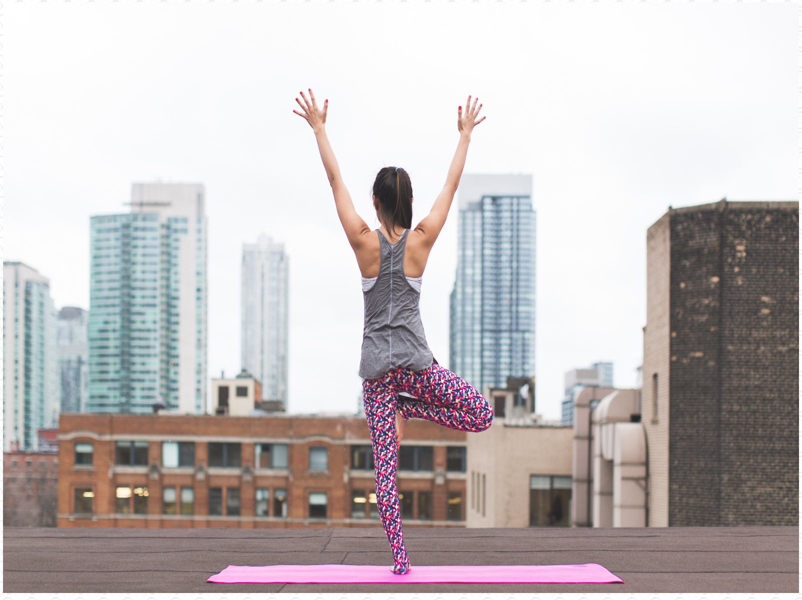

Health Tip!

All adults should avoid inactivity. Some physical activity is better than none. Any amount of physical activity helps you gain some health benefits.

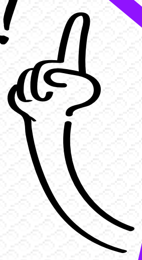

65 years and older

Older adults should follow the adult guidelines. When older adults cannot meet the adult guidelines, they should be as physically active as their abilities and conditions will allow.

Older adults with chronic conditions should understand whether and how their conditions affect their ability to do regular physical activity safe.

SAFETY FIRST!

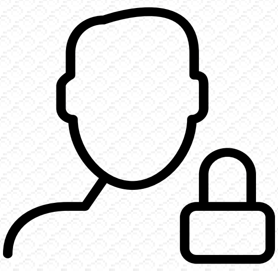

We are MPN

For a description of moderate-intensity and vigorous-intensity physical activity, as well as muscle-strengthening activity
visit: https://health.gov/dietaryguidelines/2015/guidelines/appendix-1/
