# Supplementary material for: The NUTRIENT Trial (NUTRitional Intervention among myEloproliferative Neoplasms): Results from a Randomized Phase I Pilot Study for Feasibility and Adherence
Source: Cancer Res Commun. 2024 Mar 5;4(3):660–70. doi: 10.1158/2767-9764.CRC-23-0380 (PMC10913729; doi:10.1158/2767-9764.CRC-23-0380)
Supplement: Supplementary Figure 1 — Complete blood count (CBC) data from participants. [file crc-23-0380-s02.pdf]

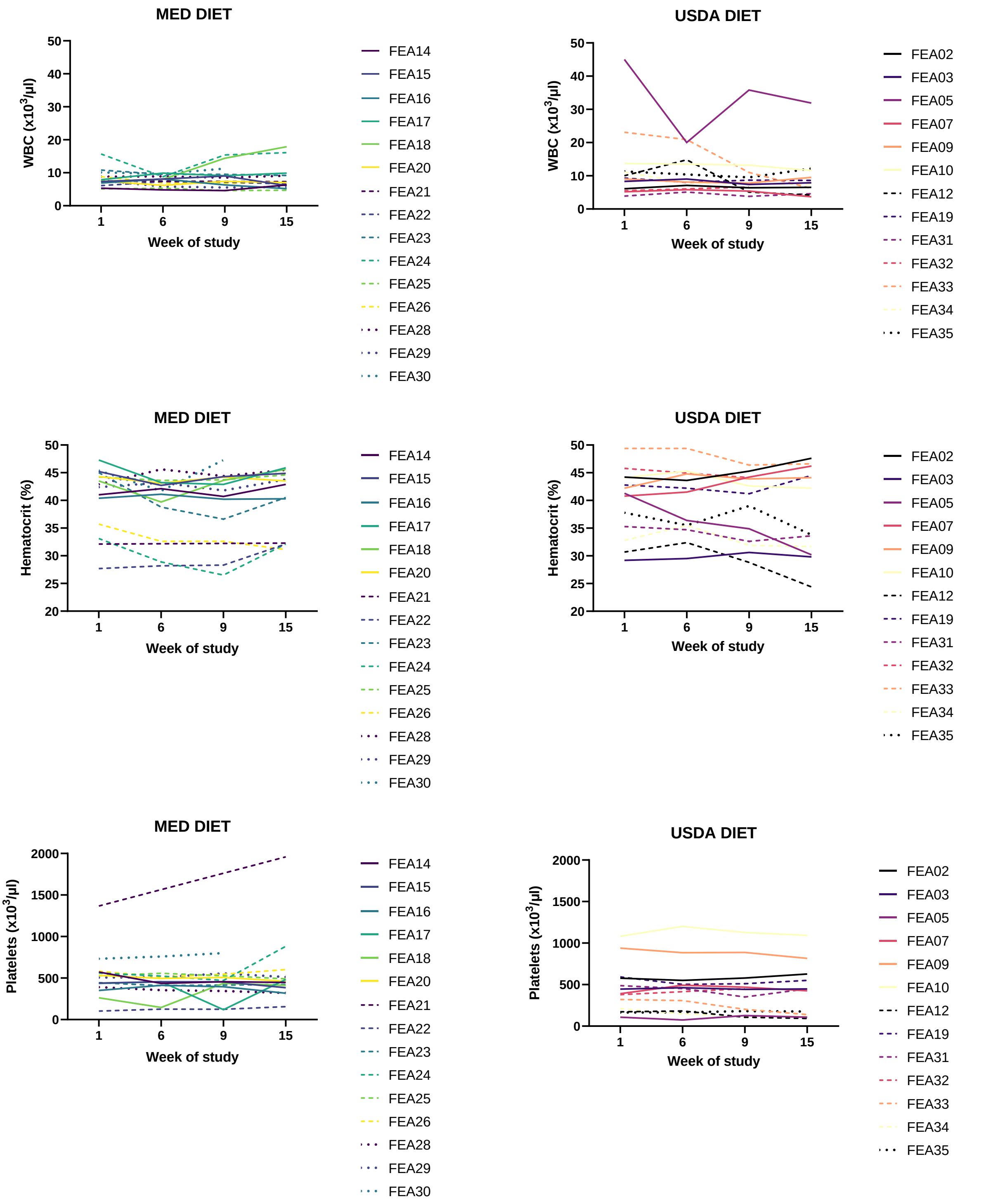

Supplemental Figure 1. Complete blood count (CBC) data from participants. CBC's were collected at weeks 1, 6, 9, and 15 from participants to monitor for changes in blood counts.
