# Supplementary material for: The NUTRIENT Trial (NUTRitional Intervention among myEloproliferative Neoplasms): Results from a Randomized Phase I Pilot Study for Feasibility and Adherence
Source: Cancer Res Commun. 2024 Mar 5;4(3):660–70. doi: 10.1158/2767-9764.CRC-23-0380 (PMC10913729; doi:10.1158/2767-9764.CRC-23-0380)
Supplement: Supplementary Figure 2 — Changes in hsCRP during the study [file crc-23-0380-s03.pdf]

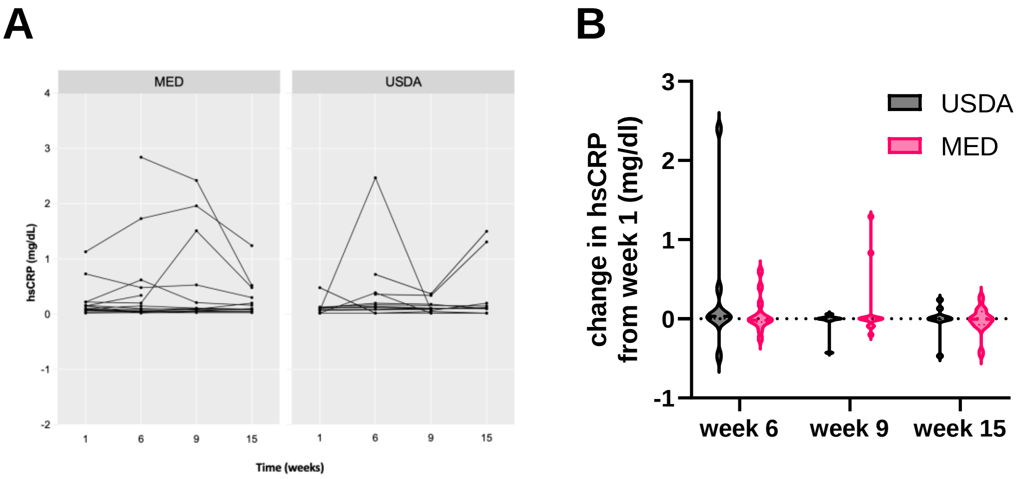

Supplemental Figure 2. Changes in hsCRP during the study. A) Spaghetti plot demonstrating raw hsCRP values for each participant during the course of the study, **B)** Violin plot depicting change in hsCRP at weeks 6, 9, and 15 using week 1 as baseline.
