# Supplementary material for: The NUTRIENT Trial (NUTRitional Intervention among myEloproliferative Neoplasms): Results from a Randomized Phase I Pilot Study for Feasibility and Adherence
Source: Cancer Res Commun. 2024 Mar 5;4(3):660–70. doi: 10.1158/2767-9764.CRC-23-0380 (PMC10913729; doi:10.1158/2767-9764.CRC-23-0380)
Supplement: Supplementary Figure 3 — Changes in JAK2V617F allele burden over time. [file crc-23-0380-s04.pdf]

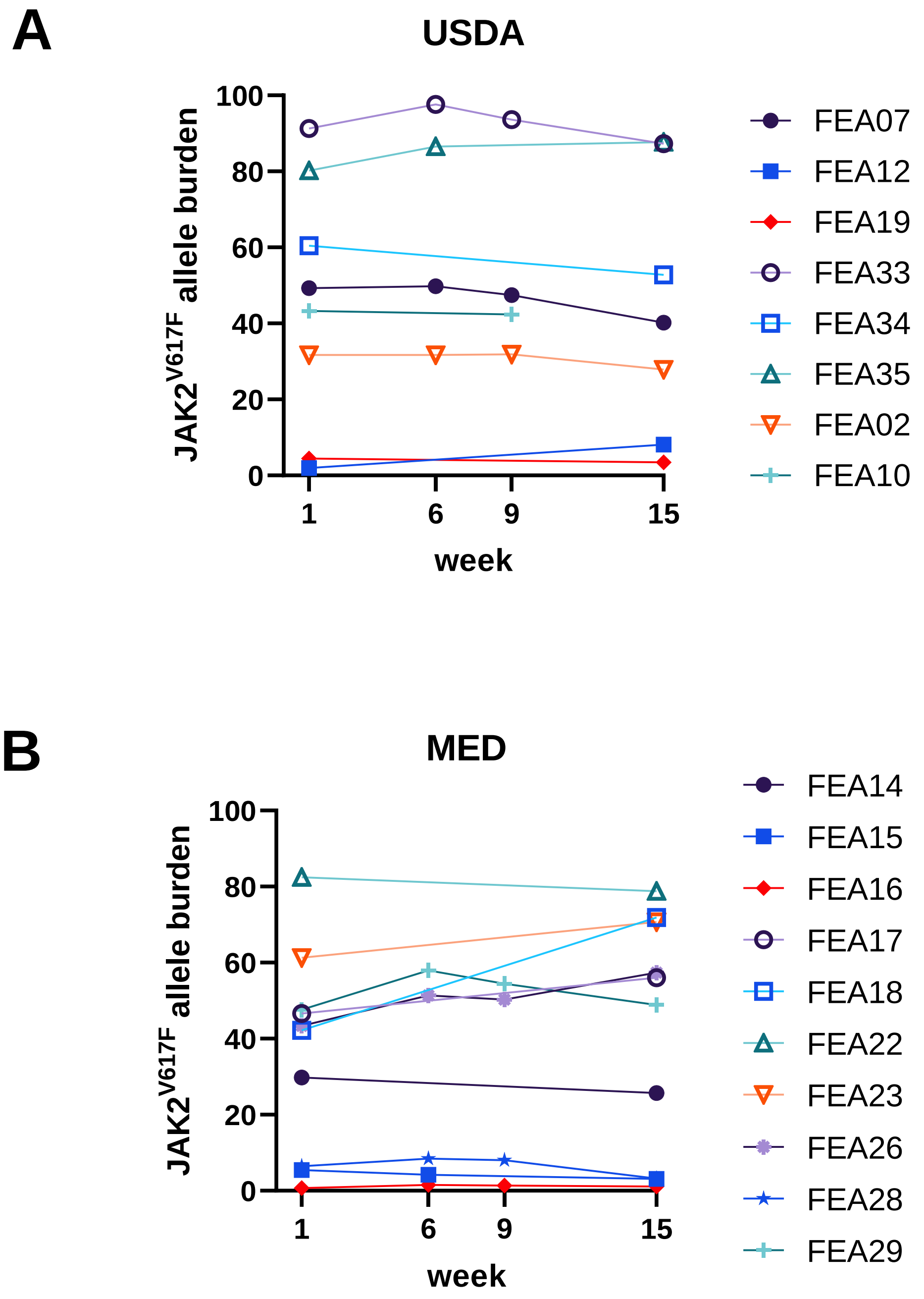

Supplemental Figure 3. Changes in *JAK2^{V617F}* allele burden over time. Whole blood from (A) USDA and (B) MED diet groups was subjected to digital PCR to quantify *JAK2^{V617F}* allele burden.
