# Supplementary material for: The NUTRIENT Trial (NUTRitional Intervention among myEloproliferative Neoplasms): Results from a Randomized Phase I Pilot Study for Feasibility and Adherence
Source: Cancer Res Commun. 2024 Mar 5;4(3):660–70. doi: 10.1158/2767-9764.CRC-23-0380 (PMC10913729; doi:10.1158/2767-9764.CRC-23-0380)
Supplement: Supplementary Table 1 — Inclusion and Exclusion Criteria [file crc-23-0380-s05.pdf]

Supplemental Table 1 – Inclusion and Exclusion Criteria

Inclusion Criteria

- Age > 18 with a diagnosis of a Philadelphia negative Myeloproliferative Neoplasm including Essential Thrombocythemia (ET), Polycythemia Vera (PV), or myelofibrosis (MF)
- Any type of MPN directed therapy is allowed
- EGOG performance status of <2
- Life expectancy > 20 weeks
- Has an email address and can access the internet
- Able to read and understand English

Exclusion Criteria

- Pregnant or planning to become pregnant over the course of the study
- Weight loss of more than 10 pounds or 10% of the total body weight over the last 6 months
- History of allergic reactions attributed to nuts or olive oil
