# Supplementary material for: The NUTRIENT Trial (NUTRitional Intervention among myEloproliferative Neoplasms): Results from a Randomized Phase I Pilot Study for Feasibility and Adherence
Source: Cancer Res Commun. 2024 Mar 5;4(3):660–70. doi: 10.1158/2767-9764.CRC-23-0380 (PMC10913729; doi:10.1158/2767-9764.CRC-23-0380)
Supplement: Supplementary Table 2 — Representativeness of Study Participants. [file crc-23-0380-s06.pdf]

**Supplemental Table 2.** Representativeness of Study Participants	
Cancer type(s)/subtype(s)/stage(s)/condition	Myeloproliferative Neoplasm
Considerations related to:	
Sex	Myeloproliferative Neoplasm affects males and females relatively equally. The incidence of polycythemia vera in males is slightly higher than that of females, however on explanation for this is that polycythemia may be underdiagnosed in females, with some female patients diagnosed with essential thrombocythemia really having masked polycythemia vera
Age	The median age of diagnosis for polycythemia vera is 60. The median age of diagnosis for essential thrombocythemia is 56. The median age of diagnosis of primary myelofibrosis is 65-70.
Race/ethnicity	MPN is more prevalent among Jews of Eastern European descent than other Europeans or Asians.
Geography	According to the Surveillance, Epidemiology, and End Results (SEER) program of the National Cancer Institute, the high range estimate is that ~20,000 people get an MPN each year and there are ~295,000 people living with an MPN in the United States.
Overall representativeness of this study	The average age in our study (58) is similar to the average age of diagnosis<br><br>We have a higher representation of females than males.<br><br>We have a larger percentage of participants from a Jewish background (18%) than expected in our catchment area, however this likely reflects the increase incidence of MPN in those with Jewish heritage.
