# Supplementary material for: The NUTRIENT Trial (NUTRitional Intervention among myEloproliferative Neoplasms): Results from a Randomized Phase I Pilot Study for Feasibility and Adherence
Source: Cancer Res Commun. 2024 Mar 5;4(3):660–70. doi: 10.1158/2767-9764.CRC-23-0380 (PMC10913729; doi:10.1158/2767-9764.CRC-23-0380)
Supplement: Supplementary Table 3 — Longitudinal complete metabolic panels of participants. [file crc-23-0380-s07.pdf]

Supplemental Table 3. Longitudinal Complete Metabolic Panel Results for study cohort

	Diet Group	Week	Na	K	Cl	CO2	Glu	BUN	Cr	GFR	Ca	Pro	Alb	AP	AST	ALT	Bili
FEA02	USDA	1	137	4	102	27	95	11	1	>60	9.5	7.7	4.7	48	21	14	1.6
		6	137	3.9	104	24	102	16	1.1	>60	9.5	7.7	4.6	54	23	18	1.3
		9	139	4	104	27	91	12	1	>60	9.3	7.6	4.5	56	22	16	1.1
		15	137	3.6	103	27	93	15	1.2	60	9.5	7.6	4.8	55	30	24	1.5
FEA03	USDA	1	139	4.2	104	26	97	17	0.8	>60	9.6	6.9	4.4	52	19	11	0.3
		6	139	4.5	103	27	97	20	0.8	>60	9.4	7	4.3	60	18	13	0.3
		9	139	4.4	104	27	99	21	0.7	>60	9.6	6.8	4.4	52	17	14	0.3
		15	139	4.3	105	27	91	24	0.7	>60	9.8	6.8	4.5	55	17	12	0.3
FEA05	USDA	1	137	4.2	104	25	101	19	1	56	9.9	6.9	4	54	57	26	2.6
		6	135	4	103	25	106	12	0.8	>60	9.1	7.3	3.3	48	41	19	2.1
		9	137	3.8	104	27	101	10	0.8	>60	9.3	7.4	3.4	51	51	23	2.3
		15	133	4.2	102	25	88	8	0.6	>60	8.9	6.9	3.4	50	47	24	2.2
FEA07	USDA	1	136	3.8	102	26	94	13	0.6	>60	9	7.5	3.9	140	37	50	0.6
		6	138	4.1	105	27	81	14	0.7	>60	9.4	7.4	4.1	98	28	37	0.5
		9	135	4	101	29	70	19	0.7	>60	9.8	7.5	4.2	100	36	54	0.6
		15	136	4.1	102	31	74	13	0.7	>60	10.1	8.1	4.5	106	30	33	0.6
FEA09	USDA	1	138	3.9	104	28	123	14	0.8	>60	9.5	7.4	4.4	75	28	29	0.5
		6	137	4.2	100	30	146	20	0.9	>60	9.8	7.6	4.6	79	36	30	0.6
		9	138	4.2	104	26	145	24	0.9	>60	9.9	7.5	4.5	72	30	24	0.6
		15	139	3.9	102	29	139	16	0.9	>60	9.7	7.4	4.6	71	25	17	0.7
FEA10	USDA	1	137	4.3	102	30	86	14	1	>60	9.7	7	4.3	54	17	12	1
		6	140	4.6	106	28	88	17	0.9	>60	9.8	7	4.7	64	18	14	0.6
		9	138	4.8	104	27	72	17	0.9	>60	10	7.2	4.5	58	24	16	1
		15	135	4.1	102	28	64	14	0.8	>60	9.5	6.7	4.6	46	23	21	0.5
FEA12	USDA	1	136	4.6	105	25	95	23	1.2	59	9.2	7.8	4.6	31	19	17	0.7
		6	137	4.5	104	22	104	29	1.4	49	9.6	8.2	4.4	59	23	34	0.8
		9	138	4.7	107	23	110	19	1.3	54	9.1	7.6	4.2	35	21	17	0.8
		15	136	4.7	107	23	97	27	1.2	59	9.4	7.8	4.4	41	22	19	0.8

FEA19	USDA	1	138	4.1	104	25	82	16	0.8	>60	9.8	7.3	4.4	61	21	20	0.4
		6	140	4	106	28	96	9	0.8	>60	9.4	7.1	4.3	62	20	18	0.5
		9	139	3.6	107	27	87	15	0.8	>60	9.4	7.2	4.4	62	22	19	0.6
		15															
FEA31	USDA	1	126	3.5	95	27	74	12	0.5	>60	9.1	6.9	4.4	56	21	16	0.4
		6	128	4.8	96	27	88	13	0.54	98	9	6.5	4.1	66	16	12	0.4
		9	128	4.6	97	27	110	15	0.62	93	8.8	6	4	59	18	14	0.4
		15	128	4.8	95	25	113	16	0.57	96	9.3	6.6	4.3	62	15	13	0.3
FEA32	USDA	1	137	4	102	27	80	11	0.7	>60	9.9	7.1	4.5	71	19	11	0.7
		6															
		9															
		15															
FEA33	USDA	1	138	4	102	28	74	11	0.7	>60	9.7	7.9	4.8	109	21	14	0.7
		6	137	4.2	102	29	74	8	0.6	>60	9.6	7.6	4.7	103	22	17	0.7
		9	138	4	103	28	99	10	0.6	>60	9.3	7.3	4.4	89	19	16	0.6
		15	137	4.3	102	30	86	12	0.7	>60	9.3	7.5	4.4	77	21	15	0.7
FEA34	USDA	1	135	3.7	103	25	73	12	0.8	>60	9.2	6.6	4.3	58	29	15	1.1
		6	139	3.9	106	26	99	13	0.8	>60	9.1	6.9	4.3	75	57	78	0.9
		9	137	4	107	25	80	12	0.8	>60	8.9	6.3	4.2	73	69	86	1.1
		15	136	4.4	105	26	107	16	0.9	>60	8.9	6.5	4.3	72	86	106	0.9
FEA35	USDA	1	140	4	102	33	63	12	0.4	>60	9.9	7.6	4.7	141	27	23	0.9
		6	139	4.5	101	31	103	11	0.5	>60	9.9	7.8	4.7	147	31	28	0.9
		9	140	4.2	103	30	93	11	0.4	>60	9.4	7	4.3	125	28	25	0.7
		15	136	4.2	100	30	112	14	0.4	>60	9.4	7.1	4.4	132	28	26	0.8
FEA14	MED	1	137	4.3	102	26	9	74	10	0.5	>60	10	7.9	4.2	56	20	15
		6	136	4	101	28	7	82	11	0.5	>60	9.9	8	4.5	51	19	14
		9	137	3.9	104	28	5	93	11	0.5	>60	9.8	7.9	4.4	54	20	14
		15	141	4	105	30	6	74	12	0.5	>60	9.8	8.1	4.4	61	19	15
FEA15	MED	1	139	3.9	103	28	8	87	15	0.9	>60	9.9	7.2	4.3	91	19	13
		6	137	3.9	101	29	7	78	17	0.9	>60	9.3	7.1	4.2	97	20	12

		9	137	3.7	101	27	9	56	17	0.9	>60	9.6	7.2	4.2	102	22	15
		15	140	3.7	102	28	10	69	21	0.9	>60	9.4	7.4	4.2	94	20	12
FEA16	MED	1	140	3.9	103	31	6	86	15	0.6	>60	9.9	6.7	4	74	16	14
		6	139	3.8	103	31	5	92	20	0.6	>60	9.8	6.9	4.3	65	16	13
		9	140	4.1	103	30	7	87	21	0.6	>60	9.6	6.6	4	80	16	15
		15	141	4	104	30	7	75	14	0.5	>60	9.1	6.3	4	90	24	33
FEA17	MED	1	140	4.1	105	28	7	81	14	1	57	9.8	7.2	4.2	75	22	21
		6	140	4.3	102	29	9	93	19	0.9	>60	10	7.3	4.3	83	21	18
		9	142	3.9	103	30	9	61	14	1	57	9.7	7	4.3	84	22	19
		15	139	4.1	101	29	9	68	18	1	57	10.1	7.6	4.5	96	27	27
FEA18	MED	1	136	4.5	104	26	6	100	14	1	>60	9.3	7.1	4.3	108	18	14
		6	137	4.3	105	24	8	86	14	0.9	>60	9.4	7.1	4	106	15	10
		9	138	5	103	26	9	104	15	1	>60	9	7.1	4.1	122	20	12
		15	139	3.9	100	27	12	37	19	1	>60	9.6	7.3	4.4	119	16	10
FEA20	MED	1	140	5	104	32	4	99	14	1	>60	10.1	7	4.7	75	22	25
		6	139	4.9	104	31	4	90	15	1.1	>60	9.5	6.5	4.2	61	24	21
		9	140	4.8	105	30	5	109	16	1	>60	9.8	6.6	4.4	60	24	23
		15	134	4.1	101	27	6	95	12	1	>60	9.6	6.7	4.3	71	25	25
FEA21	MED	1	139	4.6	105	27	7	91	8	0.6	>60	9.3	7.1	4.3	39	20	16
		6															
		9															
		15	136	3.7	101	28	7	83	15	0.7	>60	10.7	7.8	4.7	38	43	44
FEA22	MED	1	141	4.8	111	25	5	106	21	0.7	>60	9.3	6.4	4.3	61	13	9
		6	144	5.4	109	25	10	86	17	0.8	>60	9.6	6.9	4.6	65	14	9
		9	143	5.1	110	26	7	91	26	0.8	>60	9.9	6.9	4.7	61	12	6
		15	140	5.3	110	26	4	97	22	0.9	>60	9.9	6.8	4.6	60	15	10
FEA23	MED	1	140	3.5	102	32	6	77	19	0.8	>60	9.8	7.4	4.7	90	37	34
		6	141	3.2	103	30	8	76	23	0.8	>60	9.6	7.2	4.9	84	36	32
		9	140	3.7	104	31	5	85	15	0.7	>60	9.5	6.8	4.7	90	55	59
		15	140	4.1	101	32	7	76	20	0.8	>60	10.2	7.3	4.9	81	38	32

FEA24	MED	1	139	4.2	106	27	6	86	20	0.7	>60	9.3	6.3	4.2	154	34	38
		6	134	4.4	101	27	6	105	20	0.7	>60	9.6	6.4	4.4	164	48	51
		9	135	4.6	103	27	5	93	22	0.6	>60	9.5	6.7	4.3	218	59	64
		15	139	4.4	106	27	6	102	17	0.7	>60	9.4	6.5	4.3	188	44	47
FEA25	MED	1	141	4.8	103	30	8	88	18	0.8	>60	10.4	7.4	4.6	52	17	14
		6	139	4.6	101	30	8	69	16	0.7	>60	10	7.3	4.6	51	19	13
		9	137	4.2	100	30	7	43	18	0.7	>60	9.6	7.1	4.3	49	16	12
		15	137	4.6	101	31	5	64	17	0.8	>60	9.8	7.3	4.5	52	19	16
FEA26	MED	1	141	4.4	107	26	8	67	24	1	>60	8.9	6.7	4.3	65	23	23
		6	139	4.4	105	31	3	88	21	1	>60	9.4	6.4	4.4	53	17	15
		9	141	0.5	106	28	7	82	20	1	>60	9.2	6.6	4.4	56	20	14
		15	138	4.4	106	27	5	96	20	1.1	>60	9.2	6.3	4.4	56	18	14
FEA28	MED	1	136	4.3	101	28	7	83	21	0.8	>60	9.5	8.4	4.5	78	39	43
		6	138	4.4	101	28	9	68	16	0.8	>60	10.1	8.4	4.8	71	29	23
		9	135	4.6	101	28	6	81	22	0.8	>60	10	8.2	4.6	66	27	29
		15	134	4.2	99	27	8	81	18	0.8	>60	9.9	8.6	4.7	68	41	41
FEA29	MED	1	139	4.2	103	28	8	80	21	1	>60	9.5	7.3	4.7	45	20	21
		6	138	3.9	103	29	6	67	19	0.9	>60	9.7	6.9	4.5	46	26	32
		9	139	4.3	104	29	6	70	20	1	55	9.5	7.1	4.8	41	22	23
		15	142	4.3	104	28	10	69	17	1	55	9.8	7.4	4.7	39	26	25
FEA30	MED	1	137	4	100	26	11	80	14	0.9	>60	10.2	8.3	5.1	83	30	36
		6	137	4.1	101	28	8	82	13	0.9	>60	10.6	7.8	4.8	74	33	38
		9															
		15															
