# Supplementary material for: The NUTRIENT Trial (NUTRitional Intervention among myEloproliferative Neoplasms): Results from a Randomized Phase I Pilot Study for Feasibility and Adherence
Source: Cancer Res Commun. 2024 Mar 5;4(3):660–70. doi: 10.1158/2767-9764.CRC-23-0380 (PMC10913729; doi:10.1158/2767-9764.CRC-23-0380)
Supplement: Supplementary Table 4 — Longitudinal lipid values. [file crc-23-0380-s08.pdf]

Supplemental Table 4. Longitudinal Lipid Panels for Study Cohort

	Week	Diet Group	Tot Chol	TG	HDL	LDL	VLDL	Non-HDL
FEA02	1	USDA	227	159	53	142	32	174
	6		227	120	57	146	24	170
	9		227	156	51	145	31	176
	15		258	122	57	177	24	201
FEA03	1	USDA	186	200	41	105	40	145
	6		222	159	47	143	32	175
	9		208	169	48	126	34	160
	15		209	126	49	135	25	160
FEA05	1	USDA	168	109	47	108	10	
	6		123	131	21	76	26	102
	9		113	120	20	69	23	93
	15		119	126	18	76	25	101
FEA07	1	USDA	164	135	42	95	27	122
	6		161	94	48	94	19	113
	9		176	95	55	102	19	121
	15		177	104	49	107	21	128
FEA09	1	USDA						
	6		161	84	57	87	17	104
	9		172	120	48	100	24	124
	15		135	97	44	72	19	91
FEA10	1	USDA	92	58	38	42	12	54
	6		88	51	45	33	10	43
	9		97	36	47	43	7	50
	15		84	54	44	29	11	40
FEA12	1	USDA						
	6		76	60	23	41	12	53
	9		83	78	35	32	16	48
	15		69	62	27	30	12	42
FEA19	1	USDA	314	183	62	215	37	252
	6		245	155	48	166	31	197
	9		272	112	56	194	22	216
	15							
FEA31	1	USDA	185	59	58	115	12	127
	6							
	9							
	15							
FEA32	1	USDA	281	112	69	190	22	212
	6							
	9							

	15							
FEA33	1	USDA	218	353	59	88	71	159
	6		200	125	56	119	25	144
	9		204	220	58	102	44	146
	15		196	169	66	96	34	130
FEA34	1	USDA	117	157	51	35	31	66
	6		105	81	44	45	16	61
	9		110	88	50	42	18	60
	15		105	89	48	39	18	57
FEA35	1	USDA	138	381	26	36	76	112
	6		145	160	33	80	32	112
	9							
	15		130	167	31	66	33	99
FEA14	1	MED	158	85	48	93	17	110
	6		188	92	57	113	18	131
	9		158	139	50	80	28	108
	15		163	125	53	85	25	110
FEA15	1	MED	233	80	64	153	16	169
	6		196	87	52	127	17	144
	9		214	174	57	122	35	157
	15		240	83	61	162	17	179
FEA16	1	MED	172	84	55	100	17	117
	6		173	130	48	99	26	25
	9		150	148	42	78	30	108
	15		139	191	39	62	38	100
FEA17	1	MED	216	82	44	156	16	172
	6		235	139	45	162	28	190
	9		200	116	46	131	23	154
	15		224	120	46	154	24	178
FEA18	1	MED	96	88	33	45	18	63
	6		102	96	31	52	19	71
	9		105	156	24	50	31	81
	15		123	175	32	56	35	91
FEA20	1	MED	168	150	39	99	30	129
	6		150	178	40	74	36	110
	9		153	172	41	78	34	112
	15		149	126	39	85	25	110
FEA21	1	MED	120	25	68	47	5	52
	6							
	9							
	15		141	23	75	61	5	66
FEA22	1	MED	125	141	27	70	28	98

	6		125	111	28	75	22	97
	9		124	80	26	82	16	98
	15		130	111	28	80	22	102
FEA23	1	MED	251	74	94	142	15	157
	6		214	79	84	114	16	130
	9		206	71	83	109	14	123
	15		223	96	97	107	19	126
FEA24	1	MED	217	107	77	119	21	40
	6		207	98	66	121	20	141
	9		209	71	65	130	14	144
	15		205	110	60	123	22	145
FEA25	1	MED	211	141	69	114	28	142
	6		199	120	66	109	24	133
	9		202	101	63	119	20	139
	15		203	97	62	122	19	141
FEA26	1	MED	142	78	43	83	16	99
	6		139	132	37	76	26	102
	9		148	129	42	80	26	106
	15		129	73	43	71	15	86
FEA28	1	MED	204	104	49	134	21	155
	6		194	96	47	128	19	147
	9		201	205	45	115	41	156
	15		204	127	50	129	25	154
FEA29	1	MED	178	207	43	94	41	135
	6		163	233	42	74	47	121
	9		189	200	42	107	40	147
	15		170	242	41	81	48	129
FEA30	1	MED	150	154	49	70	31	101
	6		137	177	54	48	35	83
	9							
	15							
