# Supplementary material for: The NUTRIENT Trial (NUTRitional Intervention among myEloproliferative Neoplasms): Results from a Randomized Phase I Pilot Study for Feasibility and Adherence
Source: Cancer Res Commun. 2024 Mar 5;4(3):660–70. doi: 10.1158/2767-9764.CRC-23-0380 (PMC10913729; doi:10.1158/2767-9764.CRC-23-0380)
Supplement: Surveys — used in the study [file crc-23-0380-s09.pdf]

Personal Information

Thank you for your interest helping us understand how diet can affect MPN. Please fill out this brief survey on what you've been eating.

We are going to ask about what you are eating

We are going to ask you about your eating habits. Please answer honestly.

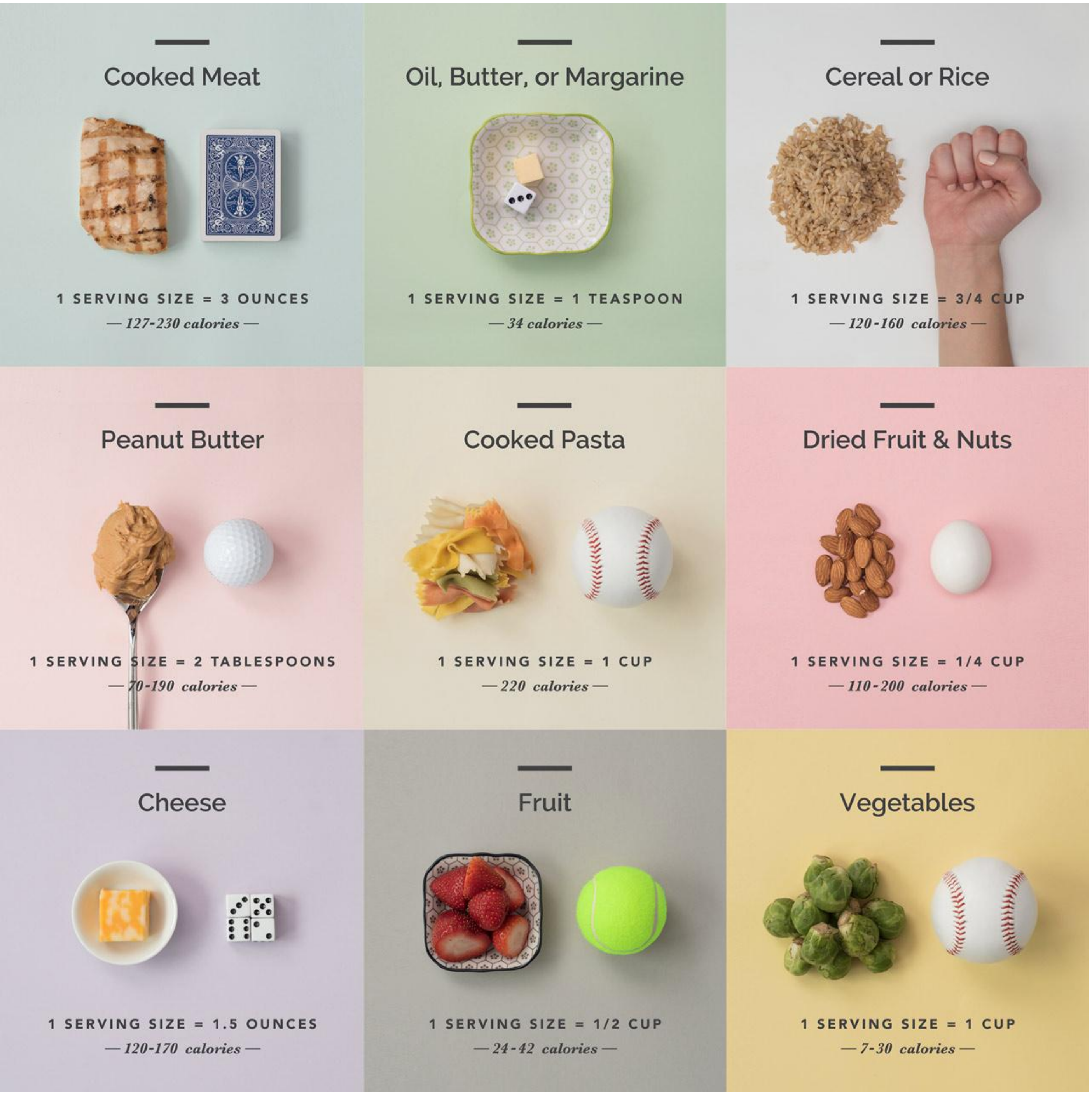

What is the primary source of fat you consume DAILY?

- ○ Olive Oil
- ○ Canola Oil

- ○ Vegetable or Corn Oil
- ○ Other Type of Oil

How much of each type of oil do you use ON AVERAGE PER DAY? (including oil used for frying, salads, out-of-house meals, etc.)

	Less than 1 Tbsp	1 - 2 Tbsp	2 - 3 Tbsp	3 - 4 Tbsp	4 Tbsp or more
Olive Oil	○	○	○	○	○
Canola Oil	○	○	○	○	○
Vegetable or Corn Oil	○	○	○	○	○
Other Type of Oil	○	○	○	○	○

How many servings of vegetables do you consume ON AVERAGE PER DAY? (1 Serving = 1 Cup of raw leafy vegetable OR 1/2 Cup of fresh, frozen, canned or cooked vegetables)

How many servings of fruit do you consume ON AVERAGE PER DAY? (1 serving = 1 medium sized fruit OR ½ cup fresh, frozen or canned fruit)

How many servings of red meat, hamburger, or meat products (ham, sausage, etc.) do you consume ON AVERAGE PER DAY? (1 serving = 1 deck of cards OR size of your palm OR 3 ounces)

How many sugar sweetened beverages do you drink ON AVERAGE PER WEEK? (such as regular soda, sweetened iced teas, lemonade, NOT diet drinks).

How many servings of butter, margarine, or cream do you consume ON AVERAGE PER DAY? (1 serving = 1 tablespoon OR 1 inch cube)

How much alcohol do you drink ON AVERAGE PER WEEK?

	Less than 1 glass	1 - 3 glasses	4 - 6 glasses	7 glasses or more
glasses of wine (any color)	○	○	○	○
bottle/cans of beer	○	○	○	○
spirits (mixed drinks or hard alcohol)	○	○	○	○
other	○	○	○	○

How many servings of legumes do you consume ON AVERAGE PER WEEK? (1 serving = 1 fist size OR 3/4 cup). Legumes include beans, soybeans, chickpeas, lentils, peas but DO NOT include peanuts.

How many servings of fish or shellfish do you consume ON AVERAGE PER WEEK? (1 serving = 1 deck of cards OR 3 ounces OR 1 cup of shellfish)

How many servings of sweets or pastries (e.g. cakes, cookies, biscuits, custard, candy, pies etc) did you consume ON AVERAGE PER WEEK? (1 serving = 1 slice cake/pie, 1 medium-sized cookie, 1 medium-sized biscuit, 1/2 Cup custard, 4-5 candies)

How many servings of nuts and seeds(including peanuts) do you consume ON AVERAGE PER WEEK? (1 serving: egg size or 1/2 cup or 30 g)

Which of the two following animal products did you eat more of?

- ○ poultry/white meat
- ○ beef/hamburger, sausage, pork, veal

How many times did you eat dishes seasoned by a vegetable based sauce (e.g. a sauce containing mixtures of onion, garlic, or additional seasoning vegetables simmered with olive oil) ON AVERAGE PER WEEK?

Powered by Qualtrics

SYMPTOM SURVEY

Items labelled 1-10 are the MPN SAF TSS (AKA MPN-10), these were used to calculate the symptom score. Questions 11-13 and the initial overall quality of life question were not used to calculate score.

	As good as it can be										As bad as it can be
What is your overall quality of life?	0	1	2	3	4	5	6	7	8	9	10

Please indicate the number that describes how much difficulty you have had with each of the following symptoms <u>DURING THE PAST WEEK</u>:

	Absent										Worst Imaginable
1. Filling up quickly when you eat (early satiety)…………………………	0	1	2	3	4	5	6	7	8	9	10
2. Abdominal discomfort………….	0	1	2	3	4	5	6	7	8	9	10
3. Inactivity………………………..	0	1	2	3	4	5	6	7	8	9	10
4. Problems with concentration – Compared to before my MPD………..	0	1	2	3	4	5	6	7	8	9	10
5. Numbness/Tingling (in my hands and feet)…………….	0	1	2	3	4	5	6	7	8	9	10
6. Night sweats……………........	0	1	2	3	4	5	6	7	8	9	10
7. Itching (pruritus)………………	0	1	2	3	4	5	6	7	8	9	10
8. Bone pain (diffuse not joint pain or arthritis)…………………..	0	1	2	3	4	5	6	7	8	9	10
9. Fever (> 100 °F)……………….	0	1	2	3	4	5	6	7	8	9	10
10. Unintentional weight loss in the last 6 months…………………	0	1	2	3	4	5	6	7	8	9	10
11. Constipation…………………………	0	1	2	3	4	5	6	7	8	9	10
12. Diarrhea……………………	0	1	2	3	4	5	6	7	8	9	10
13. Nausea……………………..	0	1	2	3	4	5	6	7	8	9	10
