# Supplementary material for: The NUTRIENT Trial (NUTRitional Intervention among myEloproliferative Neoplasms): Results from a Randomized Phase I Pilot Study for Feasibility and Adherence
Source: Cancer Res Commun. 2024 Mar 5;4(3):660–70. doi: 10.1158/2767-9764.CRC-23-0380 (PMC10913729; doi:10.1158/2767-9764.CRC-23-0380)
Supplement: USDA diet educational materials — Education materials given to the USDA group [file crc-23-0380-s10.pdf]

What's MyPlate All About?

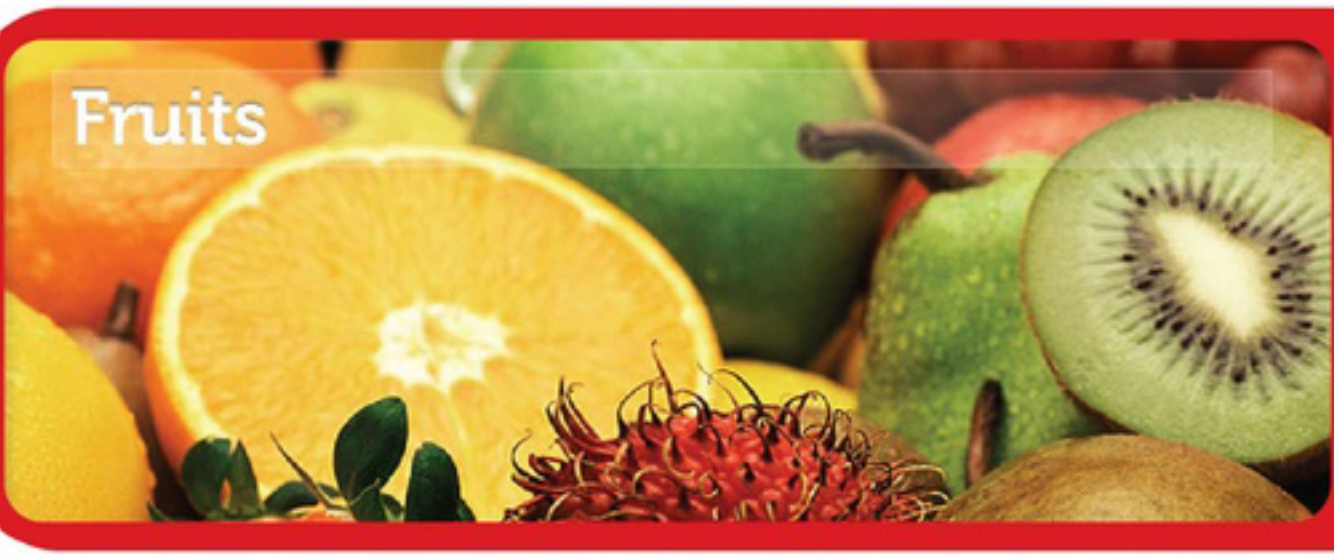

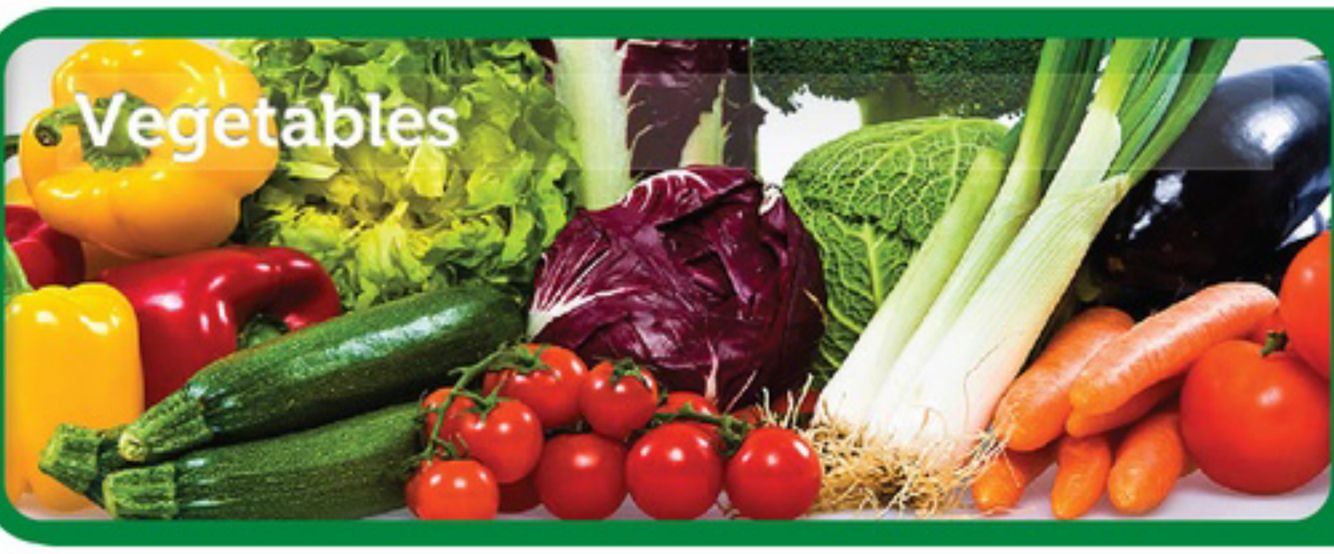

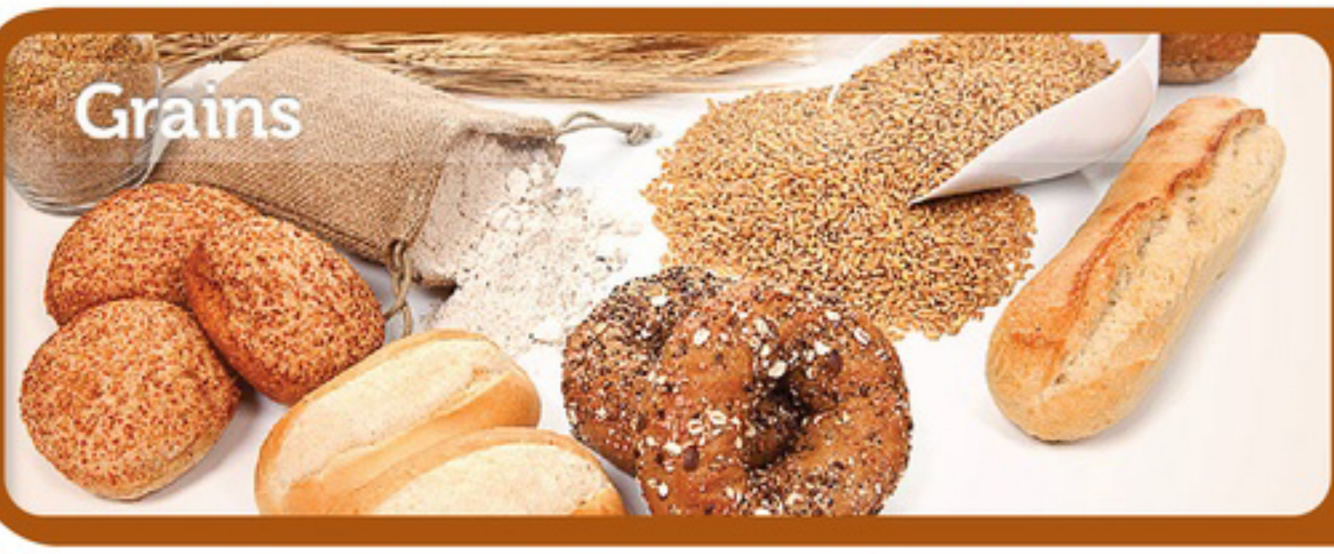

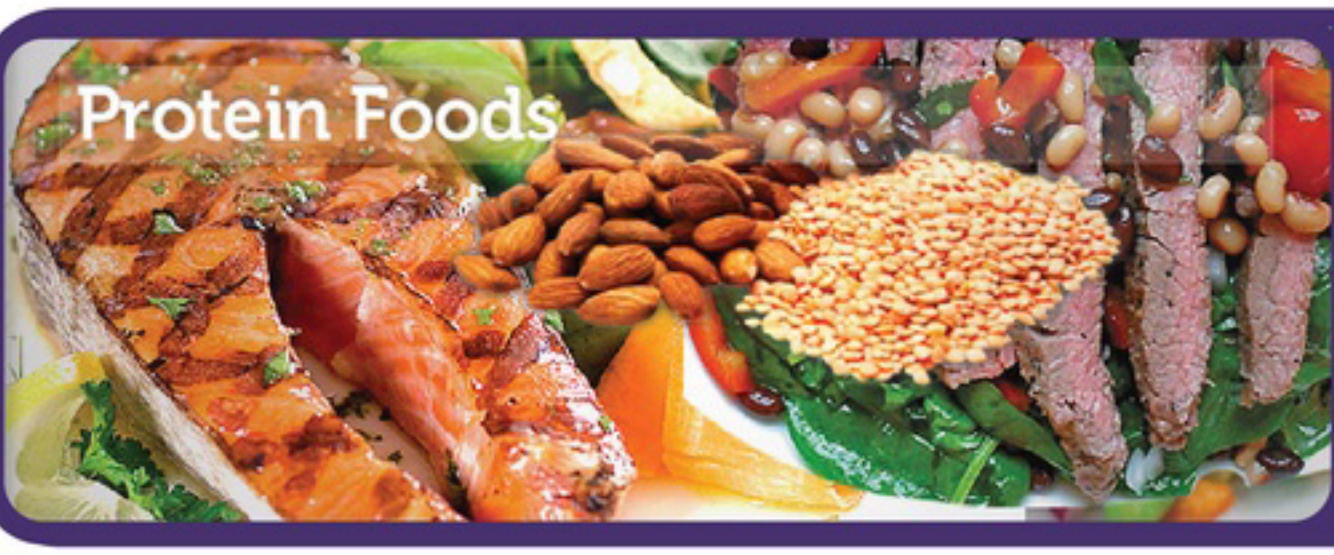

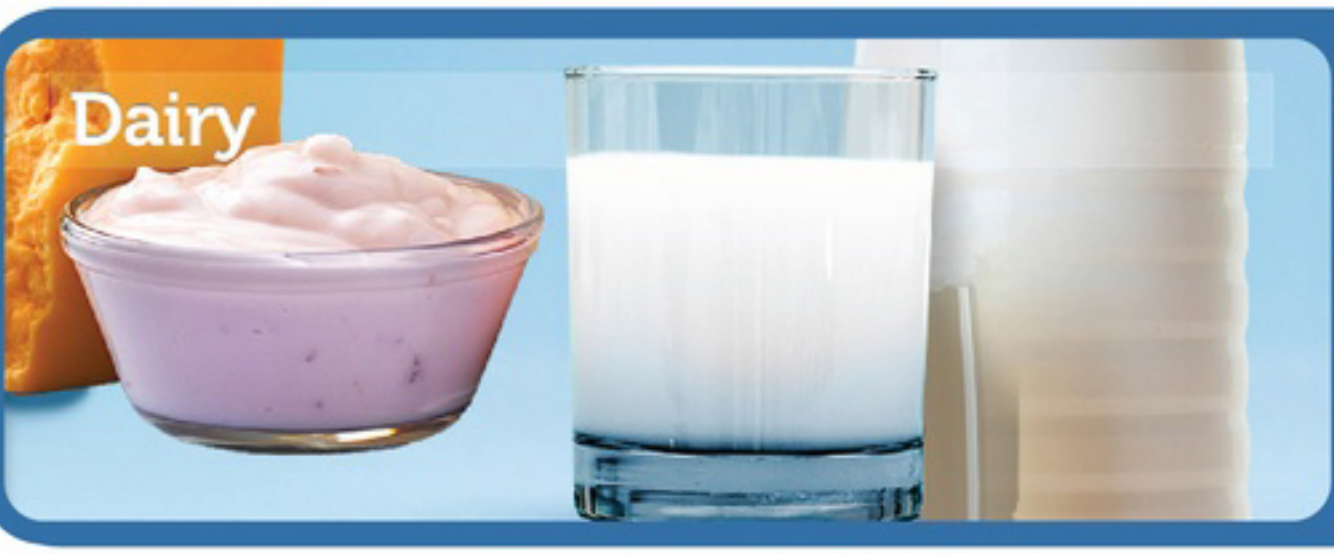

USDA United States Department of Agriculture
Center for Nutrition Policy and Promotion

USDA
United States Department of Agriculture

Find Your Healthy Eating Style & Maintain It for a Lifetime

Start with small changes to make healthier choices you can enjoy.

Follow the MyPlate building blocks below to create your own healthy eating solutions—“MyWins.” Choose foods and beverages from each food group—*making sure that your choices are limited in sodium, saturated fat, and added sugars.*

Make half your plate fruits and vegetables: Focus on whole fruits

- Choose whole fruits—fresh, frozen, dried, or canned in 100% juice.
- Enjoy fruit with meals, as snacks, or as a dessert.

Make half your grains whole grains

- Look for whole grains listed first or second on the ingredients list—try oatmeal, popcorn, whole-grain bread, and brown rice.
- Limit grain desserts and snacks, such as cakes, cookies, and pastries.

Move to low-fat or fat-free milk or yogurt

- Choose fat-free milk, yogurt, and soy beverages (soy milk) to cut back on saturated fat.
- Replace sour cream, cream, and regular cheese with low-fat yogurt, milk, and cheese.

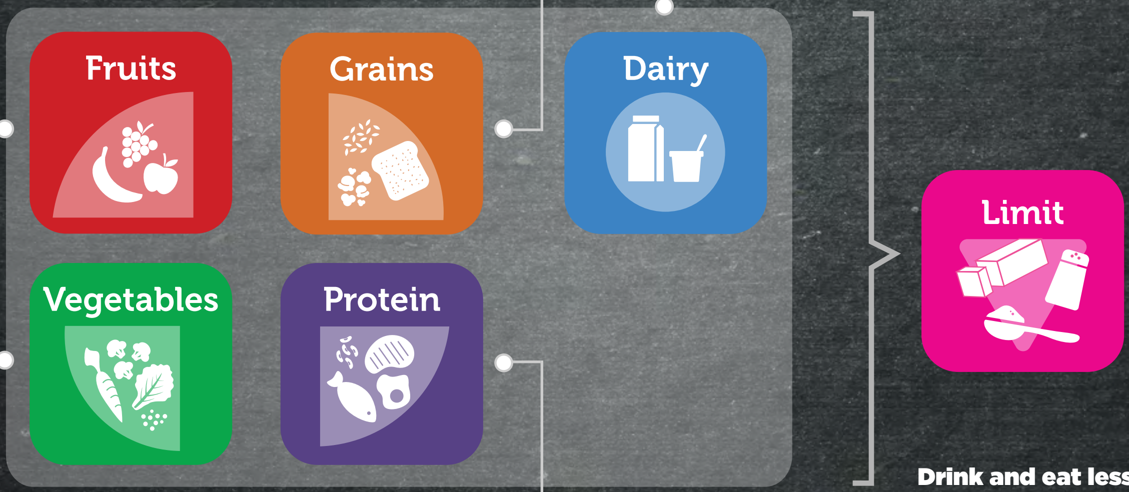

Make half your plate fruits and vegetables: Vary your veggies

- Try adding fresh, frozen, or canned vegetables to salads, sides, and main dishes.
- Choose a variety of colorful veggies prepared in healthful ways: steamed, sautéed, roasted, or raw.

Vary your protein routine

- Mix up your protein foods to include seafood, beans and peas, unsalted nuts and seeds, soy products, eggs, and lean meats and poultry.
- Try main dishes made with beans and seafood, like tuna salad or bean chili.

Drink and eat less sodium, saturated fat, and added sugars

- Use the Nutrition Facts label and ingredients list to limit items high in sodium, saturated fat, and added sugars.
- Choose vegetable oils instead of butter, and oil-based sauces and dips instead of ones with butter, cream, or cheese.
- Drink water instead of sugary drinks.

Everything you eat and drink matters.
The right mix can help you be healthier now and into the future. Find your MyWins!

Visit Choose**MyPlate**.gov *to learn more.*

Center for Nutrition Policy and Promotion
January 2016

USDA is an equal opportunity provider and employer.

10 tips
Nutrition
Education Series

MyPlate
MyWins

Based on the
Dietary Guidelines for Americans

Choose MyPlate

Use MyPlate to build your healthy eating style and maintain it for a lifetime. Choose foods and beverages from each MyPlate food group. Make sure your choices are limited in sodium, saturated fat, and added sugars. Start with small changes to make healthier choices you can enjoy.

1 Find your healthy eating style

Creating a healthy style means regularly eating a variety of foods to get the nutrients and calories you need. MyPlate's tips help you create your own healthy eating solutions—"MyWins."

2 Make half your plate fruits and vegetables

Eating colorful fruits and vegetables is important because they provide vitamins and minerals and most are low in calories.

3 Focus on whole fruits

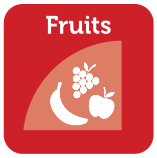

Choose whole fruits—fresh, frozen, dried, or canned in 100% juice. Enjoy fruit with meals, as snacks, or as a dessert.

4 Vary your veggies

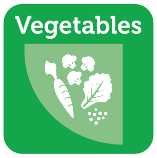

Try adding fresh, frozen, or canned vegetables to salads, sides, and main dishes. Choose a variety of colorful vegetables prepared in healthful ways: steamed, sauteed, roasted, or raw.

5 Make half your grains whole grains

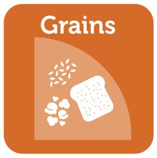

Look for whole grains listed first or second on the ingredients list—try oatmeal, popcorn, whole-grain bread, and brown rice. Limit grain-based desserts and snacks, such as cakes, cookies, and pastries.

6 Move to low-fat or fat-free milk or yogurt

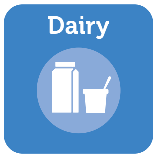

Choose low-fat or fat-free milk, yogurt, and soy beverages (soymilk) to cut back on saturated fat. Replace sour cream, cream, and regular cheese with low-fat yogurt, milk, and cheese.

7 Vary your protein routine

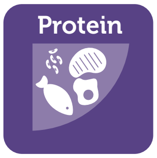

Mix up your protein foods to include seafood, beans and peas, unsalted nuts and seeds, soy products, eggs, and lean meats and poultry. Try main dishes made with beans or seafood like tuna salad or bean chili.

8 Drink and eat beverages and food with less sodium, saturated fat, and added sugars

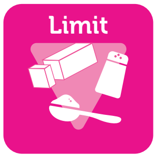

Use the Nutrition Facts label and ingredients list to limit items high in sodium, saturated fat, and added sugars. Choose vegetable oils instead of butter, and oil-based sauces and dips instead of ones with butter, cream, or cheese.

9 Drink water instead of sugary drinks

Water is calorie-free. Non-diet soda, energy or sports drinks, and other sugar-sweetened drinks contain a lot of calories from added sugars and have few nutrients.

10 Everything you eat and drink matters

The right mix of foods can help you be healthier now and into the future. Turn small changes into your "MyPlate, MyWins."

USDA
United States Department of Agriculture

10 tips Nutrition Education Series

MyPlate MyWins

Based on the Dietary Guidelines for Americans

Build a healthy meal

Each meal is a building block in your healthy eating style. Make sure to include all the food groups throughout the day. Make fruits, vegetables, grains, dairy, and protein foods part of your daily meals and snacks. Also, limit added sugars, saturated fat, and sodium. Use the MyPlate Daily Checklist and the tips below to meet your needs throughout the day.

1 Make half your plate veggies and fruits

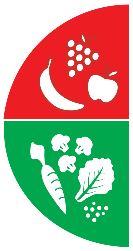

Vegetables and fruits are full of nutrients that support good health. Choose fruits and red, orange, and dark-green vegetables such as tomatoes, sweet potatoes, and broccoli.

2 Include whole grains

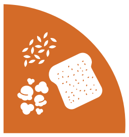

Aim to make at least half your grains whole grains. Look for the words "100% whole grain" or "100% whole wheat" on the food label. Whole grains provide more nutrients, like fiber, than refined grains.

3 Don't forget the dairy

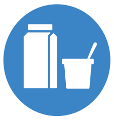

Complete your meal with a cup of fat-free or low-fat milk. You will get the same amount of calcium and other essential nutrients as whole milk but fewer calories. Don't drink milk? Try a soy beverage (soymilk) as your drink or include low-fat yogurt in your meal or snack.

4 Add lean protein

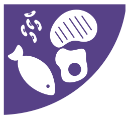

Choose protein foods such as lean beef, pork, chicken, or turkey, and eggs, nuts, beans, or tofu. Twice a week, make seafood the protein on your plate.

5 Avoid extra fat

Using heavy gravies or sauces will add fat and calories to otherwise healthy choices. Try steamed broccoli with a sprinkling of low-fat parmesan cheese or a squeeze of lemon.

6 Get creative in the kitchen

Whether you are making a sandwich, a stir-fry, or a casserole, find ways to make them healthier. Try using less meat and cheese, which can be higher in saturated fat and sodium, and adding in more veggies that add new flavors and textures to your meals.

7 Take control of your food

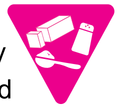

Eat at home more often so you know exactly what you are eating. If you eat out, check and compare the nutrition information. Choose options that are lower in calories, saturated fat, and sodium.

8 Try new foods

Keep it interesting by picking out new foods you've never tried before, like mango, lentils, quinoa, kale, or sardines. You may find a new favorite! Trade fun and tasty recipes with friends or find them online.

9 Satisfy your sweet tooth in a healthy way

Indulge in a naturally sweet dessert dish—fruit! Serve a fresh fruit salad or a fruit parfait made with yogurt. For a hot dessert, bake apples and top with cinnamon.

10 Everything you eat and drink matters

The right mix of foods in your meals and snacks can help you be healthier now and into the future. Turn small changes in how you eat into your MyPlate, MyWins.

10 tips Nutrition Education Series

MyPlate MyWins

Based on the **Dietary Guidelines for Americans**

Enjoy your food, but eat less

You can enjoy your meals while making small shifts to the amounts and types of food on your plate. Healthy meals start with a variety and balance of foods from each food group. Aim to consume less sodium, saturated fat, and added sugars.

1 Get to know the foods you eat

Keep track of the food and beverages you consume by using SuperTracker. This tool gives you tips on how to make healthier food choices.

2 Take your time

Be mindful to eat slowly, enjoy the taste and textures, and pay attention to how you feel. Use hunger and fullness cues to recognize when to eat and when you've had enough.

3 Use a smaller plate

Use a smaller plate at meals to help with portion control. That way you can finish your entire plate and feel satisfied without overeating.

4 If you eat out, choose healthier options

Many restaurants have nutrition information posted online or on menus. Choose entrees that are baked or broiled instead of fried. Ask for dressings or sauces on the side.

5 Satisfy your sweet tooth in a healthy way

Indulge in a naturally sweet dessert dish—fruit! Mix berries with fat-free yogurt or enjoy fruit on its own, such as melon or pineapple.

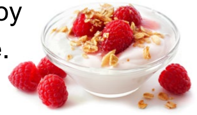

6 Choose to eat some foods more or less often

Eat more vegetables, fruits, whole grains, and low-fat dairy products. Cut back on foods such as pizza, ice cream, cookies, and cake.

7 Create your own meal plan

Plan out your meals in advance. Use Sample Meal Plans on SuperTracker for ideas to plan healthy meals.

8 Sip smarter

Drink water or other calorie-free beverages, such as unsweetened tea or club soda, or other low-calorie beverages when you are thirsty. Sugar-sweetened beverages contain added sugar and are high in calories.

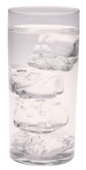

9 Compare foods

Check out the Food-A-Pedia to look up and compare nutrition information for more than 8,000 foods.

10 Make sweets a once-in-a-while treat

Treat yourself on special occasions. When you eat foods like pie, cake, brownies, cookies, and candy, choose the smallest size or consider sharing it.

10 tips Nutrition Education Series

choosing healthy meals as you get older

10 healthy eating tips for people age 65+

Making healthy food choices is a smart thing to do—no matter how old you are! Your body changes through your 60s, 70s, 80s, and beyond. Food provides nutrients you need as you age. Use these tips to choose foods for better health at each stage of life.

1 drink plenty of liquids

With age, you may lose some of your sense of thirst. Drink water often. Low-fat or fat-free milk or 100% juice also helps you stay hydrated. Limit beverages that have lots of added sugars or salt. Learn which liquids are better choices.

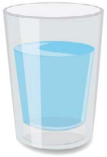

2 make eating a social event

Meals are more enjoyable when you eat with others. Invite a friend to join you or take part in a potluck at least twice a week. A senior center or place of worship may offer meals that are shared with others. There are many ways to make mealtimes pleasing.

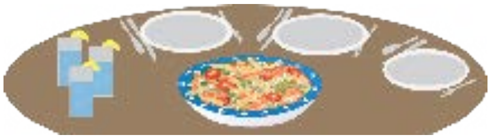

3 plan healthy meals

Find trusted nutrition information from ChooseMyPlate.gov and the National Institute on Aging. Get advice on what to eat, how much to eat, and which foods to choose, all based on the Dietary Guidelines for Americans. Find sensible, flexible ways to choose and prepare tasty meals so you can eat foods you need.

4 know how much to eat

Learn to recognize how much to eat so you can control portion size. MyPlate's SuperTracker shows amounts of food you need. When eating out, pack part of your meal to eat later. One restaurant dish might be enough for two meals or more.

5 vary your vegetables

Include a variety of different colored vegetables to brighten your plate. Most vegetables are a low-calorie source of nutrients. Vegetables are also a good source of fiber.

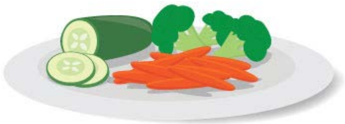

(over)

6 eat for your teeth and gums

Many people find that their teeth and gums change as they age. People with dental problems sometimes find it hard to chew fruits, vegetables, or meats. Don't miss out on needed nutrients! Eating softer foods can help. Try cooked or canned foods like unsweetened fruit, low-sodium soups, or canned tuna.

7 use herbs and spices

Foods may seem to lose their flavor as you age. If favorite dishes taste different, it may not be the cook! Maybe your sense of smell, sense of taste, or both have changed. Medicines may also change how foods taste. Add flavor to your meals with herbs and spices.

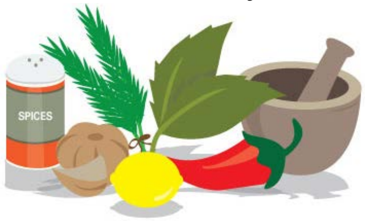

8 keep food safe

Don't take a chance with your health. A food-related illness can be life threatening for an older person. Throw out food that might not be safe. Avoid certain foods that are always risky for an older person, such as unpasteurized dairy foods. Other foods can be harmful to you when they are raw or undercooked, such as eggs, sprouts, fish, shellfish, meat, or poultry.

9 read the Nutrition Facts label

Make the right choices when buying food. Pay attention to important nutrients to know as well as calories, fats, sodium, and the rest of the Nutrition Facts label. Ask your doctor if there are ingredients and nutrients you might need to limit or to increase.

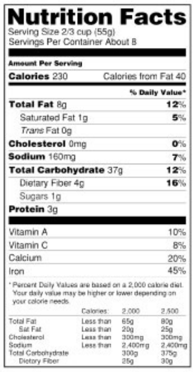

Nutrition Facts
Serving Size 2/3 cup (55g)
Servings Per Container About 8

Amount Per Serving	
**Calories** 230	Calories from Fat 40
	**% Daily Value***
**Total Fat** 8g	**12**%
Saturated Fat 1g	**5**%
Trans Fat 0g	
**Cholesterol** 0mg	**0**%
**Sodium** 160mg	**7**%
**Total Carbohydrate** 37g	**12**%
Dietary Fiber 4g	**16**%
Sugars 1g	
**Protein** 3g	
Vitamin A	10%
Vitamin C	8%
Calcium	20%
Iron	45%

* Percent Daily Values are based on a 2,000 calorie diet. Your daily value may be higher or lower depending on your calorie needs.

	Calories:	2,000	2,500
Total Fat	Less than	65g	80g
Sat Fat	Less than	20g	25g
Cholesterol	Less than	300mg	300mg
Sodium	Less than	2,400mg	2,400mg
Total Carbohydrate		300g	375g
Dietary Fiber		25g	30g

10 ask your doctor about vitamins or supplements

Food is the best way to get nutrients you need. Should you take vitamins or other pills or powders with herbs and minerals? These are called dietary supplements. Your doctor will know if you need them. More may not be better. Some can interfere with your medicines or affect your medical conditions.

Go to www.ChooseMyPlate.gov and www.nia.nih.gov/health/topics/nutrition for more information.

USDA is an equal opportunity provider and employer.

American Heart Association® life is why™ | Healthy For Good™

THE EASY WAY TO EAT HEALTHY

A healthy eating pattern is about smart choices.
The American Heart Association suggests these daily amounts.*

VEGETABLES

CANNED, DRIED, FRESH & FROZEN

5 SERVINGS or 2.5 CUPS

FRUITS

CANNED, DRIED, FRESH & FROZEN

4 SERVINGS or 2 CUPS

WHOLE GRAINS

BARLEY, BROWN RICE, MILLET, OATMEAL, POPCORN AND WHOLE WHEAT BREAD, CRACKERS & PASTA

3-6 SERVINGS or 3-6 OUNCES

DAIRY

LOW FAT (1%) AND FAT-FREE

3 SERVINGS or 3 CUPS

PROTEINS

EGGS, NON-FRIED FISH, LEAN MEAT, LEGUMES, NUTS, SKINLESS POULTRY & SEEDS

1-2 SERVINGS or 5.5 OUNCES

OILS

POLYUNSATURATED AND MONOUNSATURATED CANOLA, OLIVE, PEANUT, SAFFLOWER & SESAME OIL

3 TBSP

Food should give you energy – not weigh you down!
With a few simple changes, you can make eating healthy your easiest habit.

LIMIT

SUGARY DRINKS, SWEETS, FATTY MEATS, AND SALTY OR HIGHLY PROCESSED FOODS

AVOID

PARTIALLY HYDROGENATED OILS, TROPICAL OILS, AND EXCESSIVE CALORIES

REPLACE

HIGHLY PROCESSED FOODS WITH HOMEMADE OR LESS-PROCESSED OPTIONS

ENJOY

A VARIETY OF NUTRITIOUS FOODS FROM ALL OF THE FOOD GROUPS, ESPECIALLY FRUITS & VEGGIES

KEEP

HEALTHY HABITS EVEN WHEN YOU EAT AWAY FROM HOME

*Servings are based on AHA's Healthy US-Style Eating Pattern for 2,000 calories/day. Your calorie needs may be different. Servings equivalent may depend on form of food. More info on serving sizes is at **heart.org/servings.**

EAT SMART ***ADD COLOR*** ***MOVE MORE*** ***BE WELL***

LEARN MORE AT **HEART.ORG/HEALTHYFORGOOD**

Recommended Dietary Pattern to Achieve Adherence to the American Heart Association/American College of Cardiology (AHA/ACC) Guidelines, 2016
http://circ.ahajournals.org/content/134/22/e505, Table 2

10 tips
Nutrition Education Series

save more at the grocery store

ChooseMyPlate.gov

10 MyPlate tips to stretch your food dollar

Using coupons and looking for the best price are great ways to save money at the grocery store. Knowing how to find them is the first step to cutting costs on food. Use the MyPlate coupon tips to stretch your budget.

1 find deals right under your nose
Look for coupons with your receipt, as peel-offs on items, and on signs along aisle shelves.

2 search for coupons
Many stores still send ads and coupons for promotion, so don't overlook that so-called "junk mail." You can also do a Web search for "coupons." Go through your coupons at least once a month and toss out any expired ones.

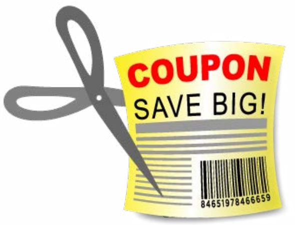

3 look for savings in newspaper
Brand name coupons are found as inserts in the paper every Sunday—except on holiday weekends. Some stores will double the value of brand name coupons on certain days.

4 join your store's loyalty program
Signup is usually free and you can receive savings and electronic coupons when you provide your email address.

5 buy when foods are on sale
Maximize your savings by using coupons on sale items. You may find huge deals such as "buy one get one free."

6 find out if the store will match competitors' coupons
Many stores will accept coupons, as long as they are for the same item. Check with the customer service desk for further details.

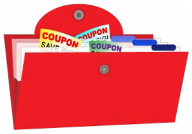

7 stay organized so coupons are easy to find
Sort your coupons either by item or in alphabetical order. Develop a system that's easiest for you and make finding coupons quick and hassle-free. Ideas for coupon storage include 3-ring binders, accordion-style organizers, or plain envelopes.

8 find a coupon buddy
Swap coupons you won't use with a friend. You can get rid of clutter and discover additional discounts.

9 compare brands
Store brands can be less expensive than some of the name brand foods. Compare the items to find better prices.

10 stick to the list
Make a shopping list for all the items you need. Keep a running list on your phone, on the refrigerator, or in a wallet. When you're in the store, do your best to buy only the items on your list.

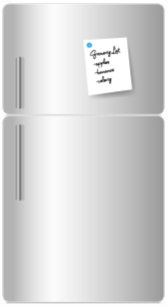

10 tips
Nutrition Education Series

smart shopping for veggies and fruits

10 tips for affordable vegetables and fruits

It is possible to fit vegetables and fruits into any budget. Making nutritious choices does not have to hurt your wallet. Getting enough of these foods promotes health and can reduce your risk of certain diseases. There are many low-cost ways to meet your fruit and vegetable needs.

1 celebrate the season

Use fresh vegetables and fruits that are in season. They are easy to get, have more flavor, and are usually less expensive. Your local farmer's market is a great source of seasonal produce.

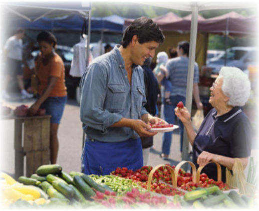

2 why pay full price?

Check the local newspaper, online, and at the store for sales, coupons, and specials that will cut food costs. Often, you can get more for less by visiting larger grocery stores (discount grocers if available).

3 stick to your list

Plan out your meals ahead of time and make a grocery list. You will save money by buying only what you need. Don't shop when you're hungry. Shopping after eating will make it easier to pass on the tempting snack foods. You'll have more of your food budget for vegetables and fruits.

4 try canned or frozen

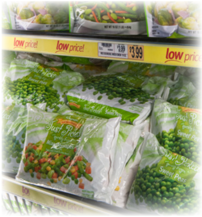

Compare the price and the number of servings from fresh, canned, and frozen forms of the same veggie or fruit. Canned and frozen items may be less expensive than fresh. For canned items, choose fruit canned in 100% fruit juice and vegetables with "low sodium" or "no salt added" on the label.

5 buy small amounts frequently

Some fresh vegetables and fruits don't last long. Buy small amounts more often to ensure you can eat the foods without throwing any away.

6 buy in bulk when items are on sale

For fresh vegetables or fruits you use often, a large size bag is the better buy. Canned or frozen fruits or vegetables can be bought in large quantities when they are on sale, since they last much longer.

7 store brands = savings

Opt for store brands when possible. You will get the same or similar product for a cheaper price. If your grocery store has a membership card, sign up for even more savings.

8 keep it simple

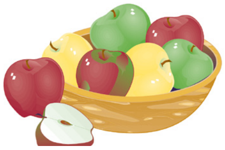

Buy vegetables and fruits in their simplest form. Pre-cut, pre-washed, ready-to-eat, and processed foods are convenient, but often cost much more than when purchased in their basic forms.

9 plant your own

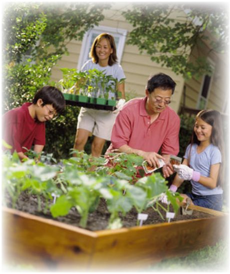

Start a garden—in the yard or a pot on the deck—for fresh, inexpensive, flavorful additions to meals. Herbs, cucumbers, peppers, or tomatoes are good options for beginners. Browse through a local library or online for more information on starting a garden.

10 plan and cook smart

Prepare and freeze vegetable soups, stews, or other dishes in advance. This saves time and money. Add leftover vegetables to casseroles or blend them to make soup. Overripe fruit is great for smoothies or baking.

USDA United States Department of Agriculture
Center for Nutrition Policy and Promotion

Go to www.ChooseMyPlate.gov for more information.

DG TipSheet No. 9
September 2011
USDA is an equal opportunity provider and employer.

So you want to eat clean?

American Heart Association | American Stroke Association
life is why

With so much conflicting information out there, how do you know what healthy eating really looks like? Let's clear up some of the myths about eating clean.

What does healthy eating look like?[1]

EAT PLENTY OF[2]

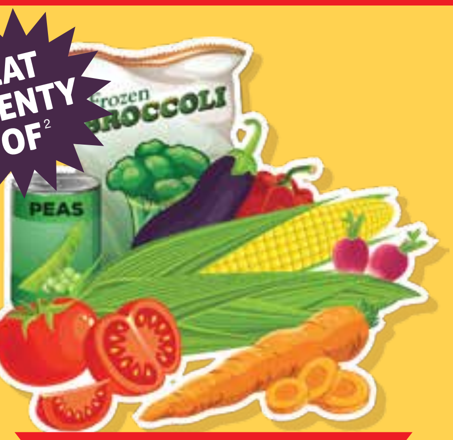

VEGETABLES

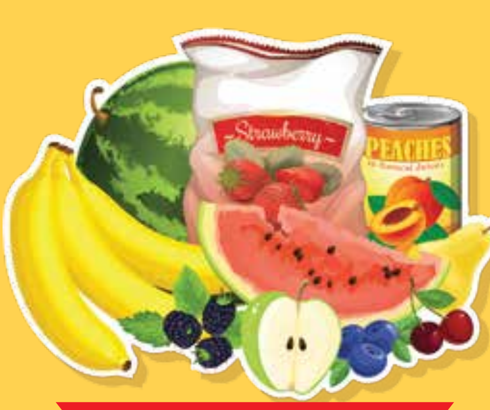

FRUITS

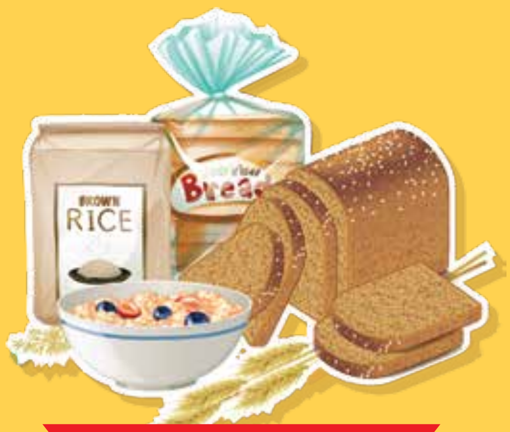

WHOLE GRAINS

Myth Only fresh fruits and vegetables are healthy.

Fact A healthy diet can include fresh, frozen, canned and dried produce.

Myth All processed foods are bad and full of chemicals.

Fact Most foods you see at the grocery store have been processed in some way. Processed foods that don't have a lot of added sugar or sodium can be a part of a healthy diet. Think: baby carrots, whole grain bread, plain yogurt, or chopped nuts!

Healthy Eating TIPS

- Select canned and frozen fruit and vegetables without salty sauces or sugary syrups.
- Drain and rinse canned products to get rid of some of the additives like salt and sugar.

Include these foods[3]:

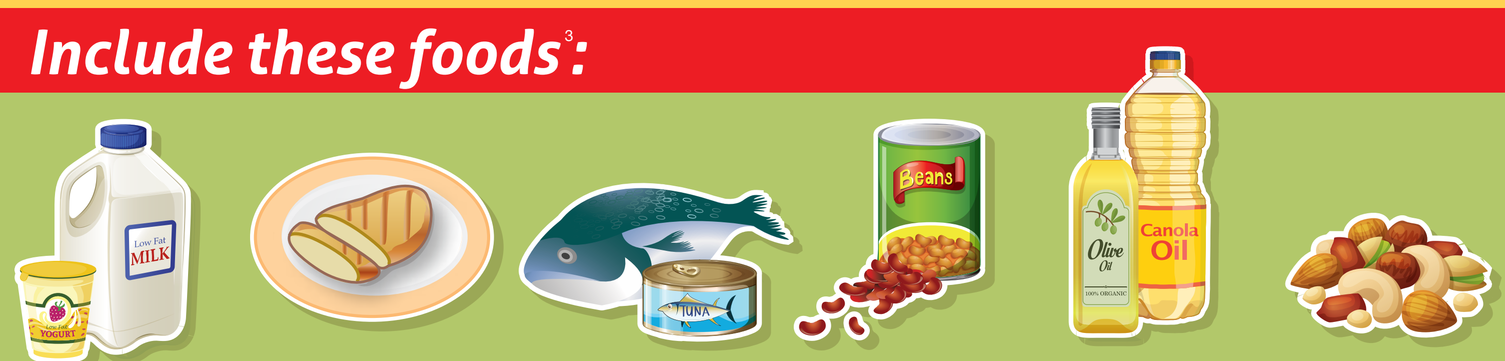

Low-Fat & Non-Fat Dairy

Skinless Poultry

Fish

Beans & Legumes

Nontropical Vegetable Oils

Nuts & Seeds

Healthy Eating TIPS

- Prepare food at home to control what is added.
- Bring out the natural flavors in foods by using healthier cooking methods like grilling, braising, roasting, searing, and sautéing.
- Add flavor with delicious herbs, spices, black pepper and citrus juices instead of sugar, salt and unhealthy fats.

Myth All foods labeled "natural" are good for you.

Fact When it comes to food packaging, there is no official definition for the term "natural."[4]

Myth If I recognize the ingredients on the label, I have nothing to worry about.

Fact Even if you recognize the ingredients, the food may still have too much sodium, added sugars, and unhealthy fats.

Watch for and limit[5]:

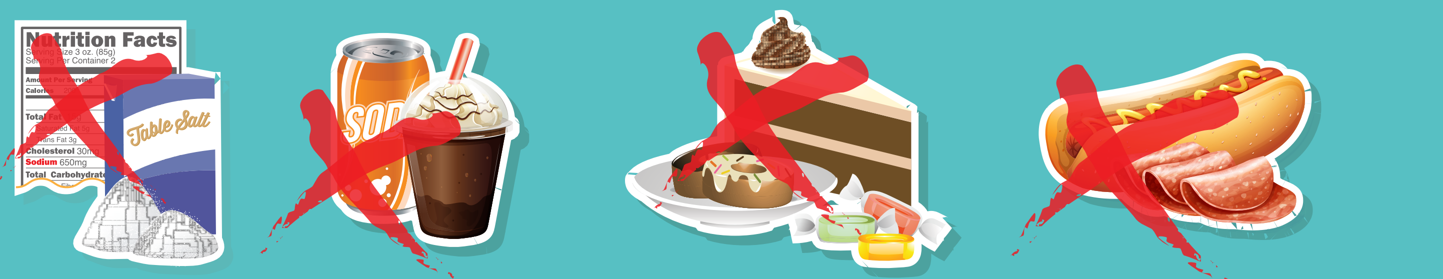

Salt/Sodium

Sugary Drinks

Sweets

Fatty or Processed Meats – choose lean or extra-lean meats instead

Healthy Eating TIPS

- Compare food labels and choose nutritious foods with the lowest amounts of sodium.
- Be aware of portion and serving sizes and total calories eaten.

Myth I should avoid the middle aisles of the grocery store.

Fact There are many foods throughout the grocery store that can be part of a healthy eating pattern.

Want more control over the foods you buy at the grocery store and eat at restaurants? Join our movement to tell the food industry you want healthier ingredients. Your voice matters!

Take action at **heart.org/sodium.**

[1,2,3,5] Van Horn, Linda, Jo Ann S. Carson, Lawrence J. Appel, Lora E. Burke, Christina Economos, Wahida Karmally, Kristie Lancaster, Alice H. Lichtenstein, Rachel K. Johnson, Randal J. Thomas, Miriam Vos, Judith Wylie-Rosett and Penny Kris-Etherton. Recommended Dietary Pattern to Achieve Adherence to the American Heart Association/American College of Cardiology (AHA/ACC) Guidelines: A Scientific Statement From the American Heart Association. Circulation. 2016; CIR.0000000000000462, originally published October 27, 2016.

[4] Two sources: http://www.fda.gov/AboutFDA/Transparency/Basics/ucm214868.htm and http://www.fda.gov/Food/GuidanceRegulation/GuidanceDocumentsRegulatoryInformation/LabelingNutrition/ucm456090.htm

©2017, American Heart Association 3/17DS12099

Can Processed Food Be Healthy?

American Heart Association | American Stroke Association
life is why®

There are a lot of conflicting messages about what processed food is.

Most of the food we eat today has been processed in some way, from salad mix to frozen dinners. Some processed foods have ingredients added, some are fortified to add nutrients, some are prepared for convenience, and some are packaged to last longer or for food safety. Even foods labeled "natural" or "organic" can be processed.

More people are paying attention to processed food.

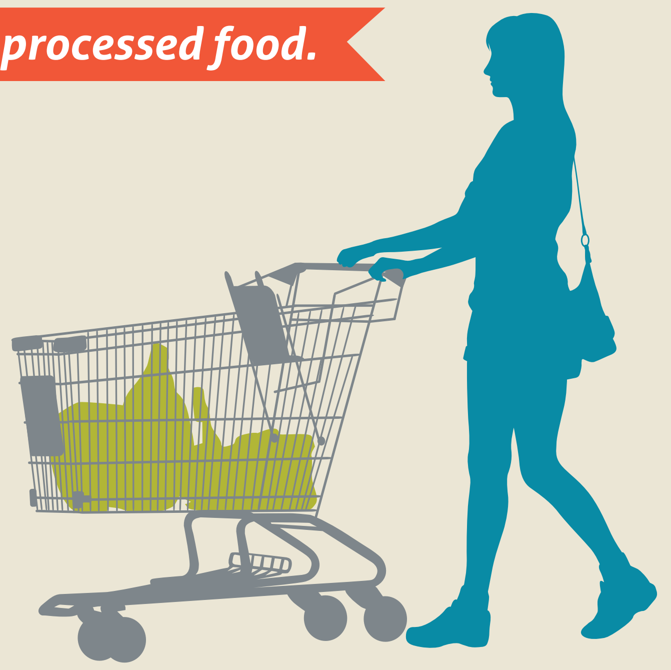

74% of consumers prefer less sodium in processed foods.

Almost **50%** of consumers have tried to eat fewer processed foods.

What do you need to know?

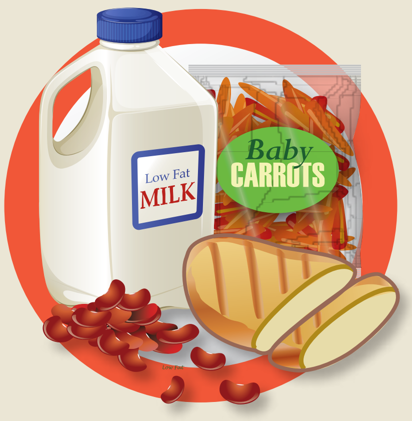

Minimally processed foods have been manipulated (cut, cooked, packaged) in some way.

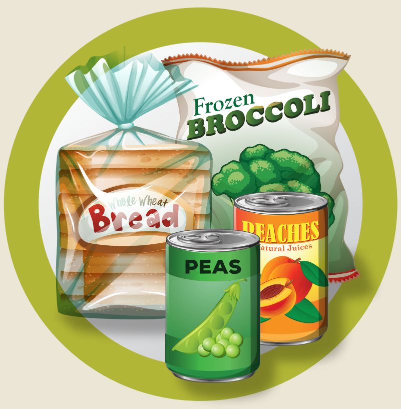

Some foods are processed with ingredients typically used in cooking, such as salt or sugar.

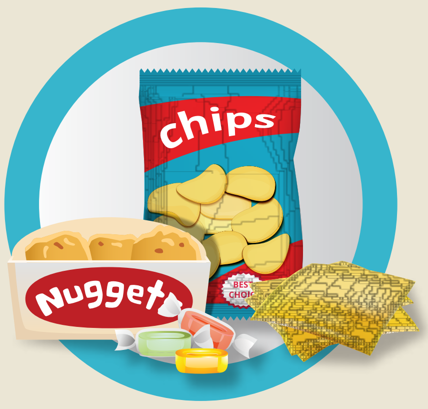

Highly processed foods are manufactured with ingredients that are not typically used in cooking.

1 *Choose healthier processed foods.*

By one recent estimate *highly processed foods* contribute **50%** of the calories & **90%** of added sugars *in the American diet*

It's important to:

- Read food labels.
- Look for the *Heart-Check mark* on packaged foods.
- Make healthier choices when eating out.

American Heart Association CERTIFIED Meets Criteria For Heart-Healthy Food

2 *Seek healthier alternatives to highly processed foods.*

Cook more meals at home.

Swap highly processed foods with less processed options.

Try fruits and vegetables from the produce aisle, the farmer's market, or your own garden.

3 *Watch out for sneaky sodium.*

Extensive research has shown that too much sodium is related to high blood pressure, a primary risk factor for heart disease and stroke.

Shake your sodium habit.

Most of the sodium we eat comes from **PROCESSED, PREPACKAGED, AND RESTAURANT FOODS,** *NOT THE SALT SHAKER.*

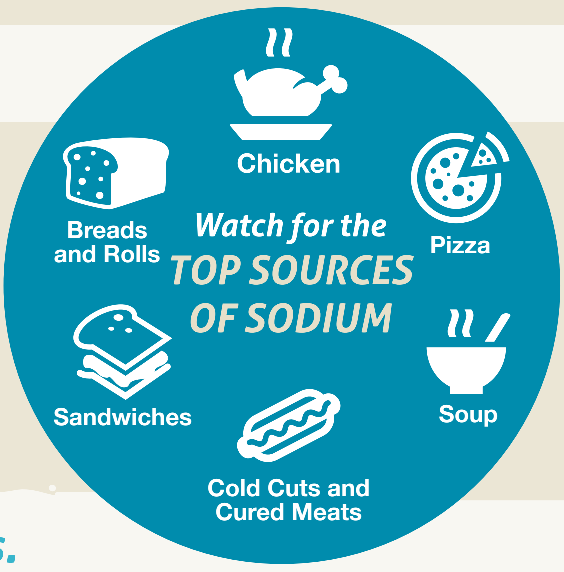

4 *Take your food into your own hands.*

American Heart Association advocates have written **more than 29,000 letters to the food companies and restaurants** that provide processed foods, asking that healthier options be made available.

You can too! Join our growing community, take action, get helpful tips and #BreakUpWithSalt today by visiting **heart.org/sodium**.

© 2017 American Heart Association 4/17DS12133

EAT SMART

WITH FOOD NUTRITION LABELS

The Nutrition Facts label can help you make healthier choices. ***Use it!*** *Here's what to look for:*

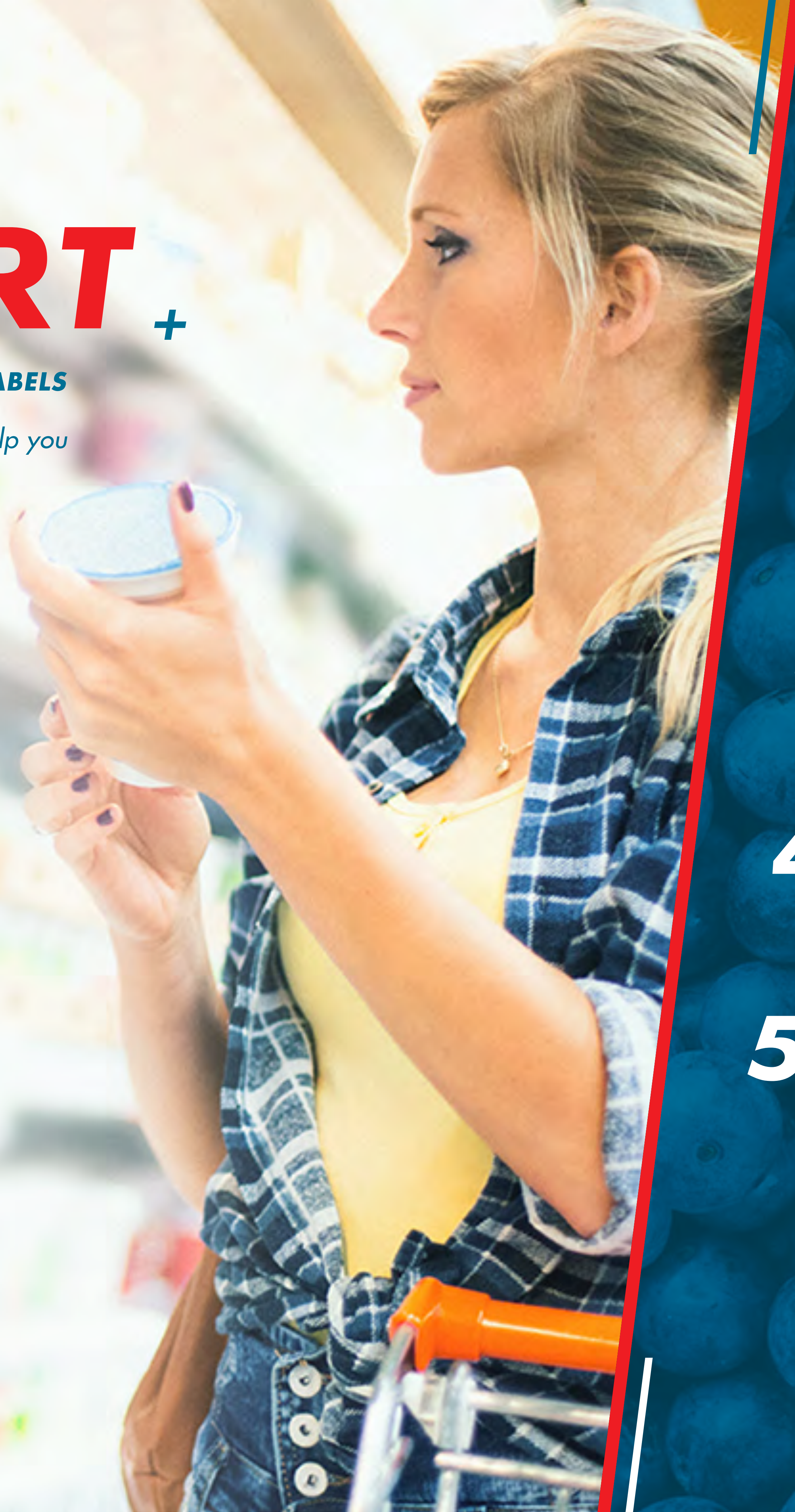

Nutrition Facts

8 servings per container	
**Serving size**	**2/3 cup (55g)**
**Amount per serving**	
**Calories**	**230**
	**% Daily Value***
**Total Fat** 8g	**10%**
Saturated Fat 1g	**5%**
Trans Fat 0g	
**Cholesterol** 0mg	**10%**
**Sodium** 160mg	**7%**
**Total Carbohydrate** 37g	**13%**
Dietary Fiber 4g	**14%**
Total Sugars 12g	
Includes 10g Added Sugars	**20%**
**Protein** 3g	
Vitamin D 2mcg	10%
Calcium 260mg	20%
Iron 8mg	45%
Potassium 235mg	6%

*The % Daily Value (DV) tells you how much a nutrient in a serving of food contributes to a daily diet. 2,000 calories a day is used for general nutrition advice.

(Label callouts: 1 – servings/serving size; 2 – calories; 3 – total fat through added sugars; 4 – protein through potassium; 5 – footnote)

For more tips and tricks on eating smart, visit
HEART.ORG/EATSMART

American Heart Association® | Healthy For Good™

1 Start with serving information.
This will tell you the size of a single serving and how many servings are in the package.

2 Check total calories per serving.
Do the math to know how many calories you're really getting if you eat the whole package.

3 Limit certain nutrients.
Try to keep saturated fat, trans fat and sodium as low as possible.

4 Get enough of beneficial nutrients.
Eat foods with dietary fiber, protein, calcium, iron, vitamins and other nutrients your body needs.

5 Quick guide to % Daily Value.
- The % Daily Value (DV) tells you the percentage of each nutrient in a single serving in terms of the daily recommended amount.
- To consume less of a nutrient (such as saturated fat or sodium), choose foods with a lower % DV — 5% or less.
- To consume more of a nutrient (such as fiber), choose foods with a higher % DV — 20% or more.

American Heart Association
life is why™
Healthy For Good™

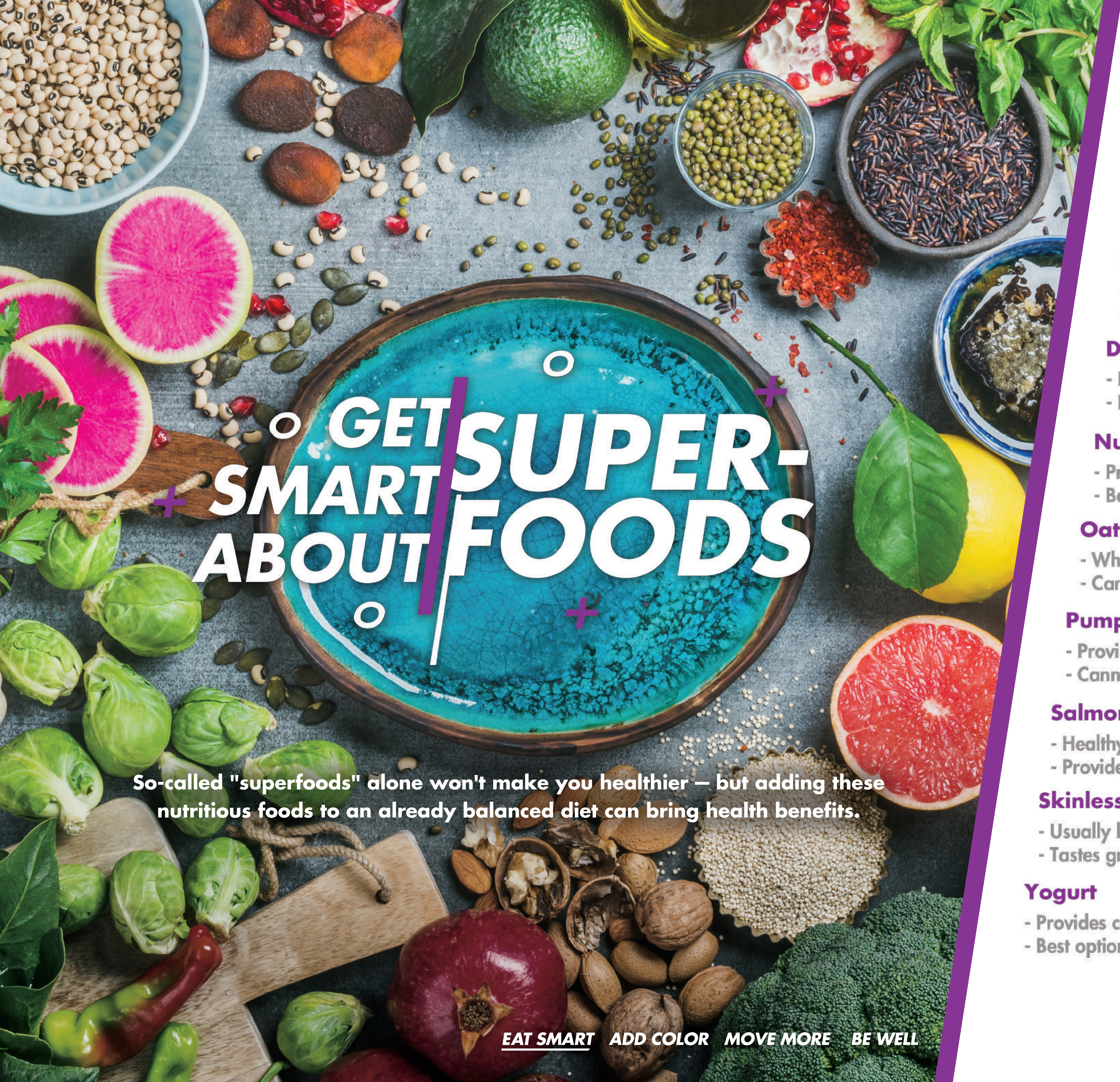

GET SMART ABOUT SUPER-FOODS

So-called "superfoods" alone won't make you healthier — but adding these nutritious foods to an already balanced diet can bring health benefits.

EAT SMART ADD COLOR MOVE MORE BE WELL

Beans & Legumes
- Economical, plant-based source of protein
- Provide fiber, magnesium and phytonutrients

Berries
- High level of flavonoids
- Can lower risk of heart attack in women

Dark Leafy Greens
- Packed with nutrients, fiber and antioxidants
- Low in calories and carbohydrates

Nuts & Seeds
- Provide protein, fiber and unsaturated fats
- Best options are unsalted

Oats
- Whole-grain source of dietary fiber
- Can lower risk of heart disease, stroke and diabetes

Pumpkin
- Provides fiber, potassium and vitamin A
- Canned is a convenient, nutrient-loaded choice

Salmon
- Healthy protein
- Provides Omega-3 Fatty Acids

Skinless Poultry
- Usually leaner than beef
- Tastes great grilled, roasted or baked

Yogurt
- Provides calcium, protein & vitamin D
- Best options are low-fat or fat-free

LEARN MORE AT
HEART.ORG/HEALTHYFORGOOD

10 tips
Nutrition
Education Series

MyPlate MyWins

Based on the Dietary Guidelines for Americans

Liven up your meals with vegetables and fruits

Discover the many benefits of adding vegetables and fruits to your meals. Vegetables and fruits don't just add fiber and key nutrients to meals. They also add color, flavor, and texture. Explore these creative ways to bring healthy foods to your table.

1 Fire up the grill

Use the grill to cook vegetables and fruits. Try grilling mushrooms, onions, peppers, or zucchini on a kabob skewer. Brush with oil to keep them from drying out. Grilled fruits like peaches, pineapple, or mangos add variety to a cookout.

2 Take your casserole to the next level

Mix vegetables such as sauteed onions, peas, pinto beans, or tomatoes into your favorite dish for that extra flavor.

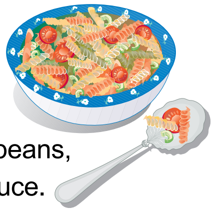

3 Planning something Italian?

Add extra vegetables to your pasta dish. Slip some herbs, peppers, spinach, red beans, onions, or cherry tomatoes into your tomato sauce. Vegetables provide texture that satisfies.

4 Get creative with your salad

Toss in shredded carrots, peas, orange segments, strawberries, or other seasonal items for a flavorful, fun salad.

5 Salad bars aren't just for vegetables

In addition to vegetables, add fruit, egg, cottage cheese, beans, or seeds from the salad bar for a variety of toppings from all the food groups.

6 Get in on the stir-frying fun

Try something new! Stir-fry fresh or frozen veggies—like broccoli, carrots, cauliflower, or green beans—for a quick-and-easy addition to any meal.

7 Add them to your sandwiches

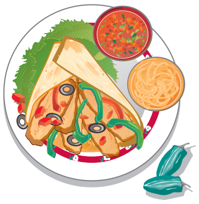

Whether it is a sandwich or wrap, vegetables make great additions to both. Try hummus, cucumber, or avocado on your usual sandwich or wrap for extra flavor.

8 Be creative with your breakfast

Add apples, bananas, blueberries, or pears to your oatmeal, yogurt, or pancakes for a special start to your day.

9 Make a tasty fruit smoothie

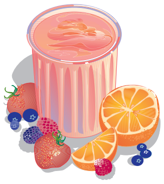

Blend fresh or frozen berries and bananas with 100% fruit juice for a delicious frozen fruit smoothie.

10 Liven up an omelet

Boost the color and texture of your morning omelet with vegetables. Simply chop, saute, and add them to the egg as it cooks. Try combining different vegetables, such as mushrooms, spinach, green onions, or bell peppers.

10 tips
Nutrition Education Series

MyPlate MyWins

Based on the **Dietary Guidelines for Americans**

Add more vegetables to your day

It's easy to eat more vegetables! Eating vegetables is important because they provide vitamins and minerals and most are low in calories. To fit more vegetables in your day, try them as snacks and add them to your meals.

1 Discover fast ways to cook

Cook fresh or frozen vegetables in the microwave for a quick-and-easy dish to add to any meal. Steam green beans, carrots, or bok choy in a bowl with a small amount of water in the microwave for a quick side dish.

2 Be ahead of the game

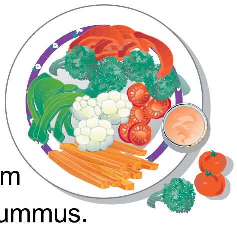

Cut up a batch of bell peppers, cauliflower, or broccoli. Pre-package them to use when time is limited. Enjoy them in a casserole, stir-fry, or as a snack with hummus.

3 Choose vegetables rich in color

Brighten your plate with vegetables that are red, orange, or dark green. They are full of vitamins and minerals. Try acorn squash, cherry tomatoes, sweet potatoes, or collard greens. They not only taste great but are good for you, too.

4 Check the freezer aisle

Frozen vegetables are quick and easy to use and are just as nutritious as fresh veggies. Try adding frozen vegetables, such as corn, peas, edamame, or spinach, to your favorite dish. Look for frozen vegetables without added sauces, gravies, butter, or cream.

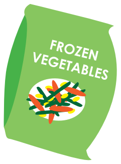

5 Stock up on veggies

Canned vegetables are a great addition to any meal, so keep on hand canned tomatoes, kidney beans, garbanzo beans, mushrooms, and beets. Select those labeled as "reduced sodium," "low sodium," or "no salt added."

6 Make your garden salad glow with color

Brighten your salad by using colorful vegetables such as black beans or avocados, sliced red bell peppers or onions, shredded radishes or carrots, and chopped red cabbage or watercress. Your salad will not only look good but taste good, too.

7 Sip on some vegetable soup

Heat it and eat it. Try tomato, butternut squash, or garden vegetable soup. Look for reduced- or low-sodium soups. Make your own soups with a low-sodium broth and your favorite vegetables.

8 While you're out

If dinner is away from home, no need to worry. When ordering, ask for an extra side of vegetables or a side salad instead of the typical fried side dish. Ask for toppings and dressings on the side.

9 Savor the flavor of seasonal vegetables

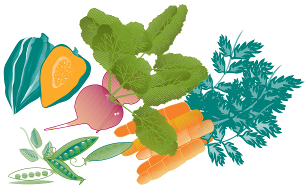

Buy vegetables that are in season for maximum flavor at a lower cost. Check your local supermarket specials for the best in-season buys. Or visit your local farmers market.

10 Vary your veggies

Choose a new vegetable that you've never tried before. Find recipes online at WhatsCooking.fns.usda.gov.

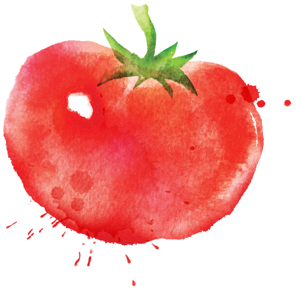

TOMATO

WHAT IS IT?

One of America's most popular garden veggies, the tomato comes in hundreds of varieties. Enjoy fresh in the summer and canned year-round.

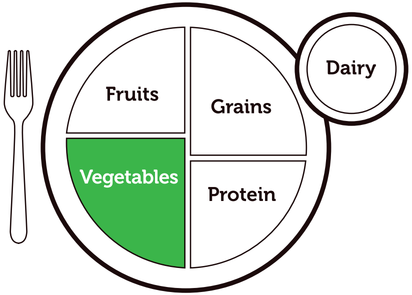

VARY YOUR VEGGIES

Aim to make half your plate fruits and vegetables. Tomatoes are a nutritious addition to help you get there.

HOW IT FITS INTO MYPLATE

A 2,000 calorie diet has a daily Vegetable Group target of 2½ cups. By eating 1 large tomato, you're almost halfway there!

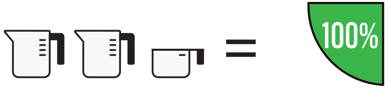

2½ CUPS = VEGGIE GROUP TARGET

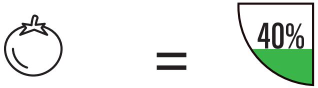

1 LARGE TOMATO (1 CUP) = VEGGIE GROUP TARGET

To find your food group targets, go to **ChooseMyPlate.gov/Checklist**

FUN FACTS & TIPS

Though tomatoes are botanically a fruit, the Supreme Court ruled them a vegetable in 1893.

Add slices of tomato to your sandwich as an easy way to work toward your Vegetable Group target.

Tomatoes are versatile! Mix them with melon for a fresh summer salad.

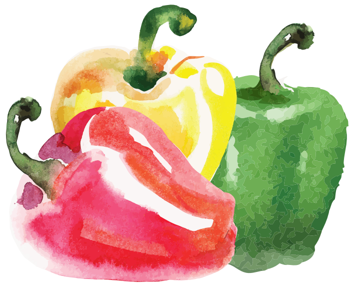

BELL PEPPER

WHAT IS IT?

Peppers grow in many shapes, sizes, colors, and flavors from sweet bell peppers to spicy chili peppers.

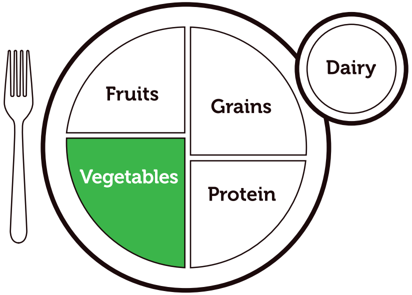

VARY YOUR VEGGIES

Different color peppers have different benefits. Eating a variety of colors throughout the week can help you vary your veggies.

HOW IT FITS INTO MYPLATE

A 2,000 calorie diet has a daily Vegetable Group target of 2½ cups. Add different types of peppers to meals and snacks to reach your goal!

2½ CUPS = 100% VEGGIE GROUP TARGET

1 LARGE BELL PEPPER (1 CUP) = 40% VEGGIE GROUP TARGET

To find your food group targets, go to **ChooseMyPlate.gov/Checklist**

FUN FACTS & TIPS

- Thomas Jefferson grew a variety of peppers at his house in Monticello, VA.
- Peppers are high in Vitamin C—add them to omelets, stir fries, or chili for added flavor.
- Red bell peppers are simply ripened green bell peppers.
- For a crunchy and filling snack, try pairing pepper slices with hummus dip.

MyPlate MyWins

For more information go to **ChooseMyPlate.gov**
USDA is an equal opportunity provider, employer, and lender.

August 2017

USDA

Frozen Broccoli Five Ways

Cream of Broccoli Soup

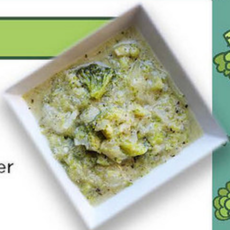

- Frozen broccoli
- Chicken broth
- Milk (non-fat)
- Onion
- Thyme
- Bay leaves
- Margarine
- Flour
- Salt
- Pepper
- Garlic powder

Brag About it Bread Bake

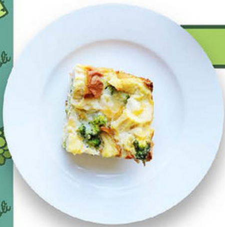

- Frozen broccoli
- Eggs
- Egg whites
- Milk (non-fat)
- Cheddar cheese (low-fat)
- Onion
- Bread
- Chicken

Magic Crust Quiche

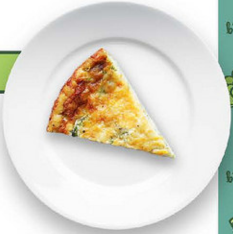

- Frozen broccoli
- Onion
- Cauliflower
- Cheddar cheese (low-fat)
- Eggs
- Milk (low-fat)
- Vegetable oil

Broccoli and Corn Bake

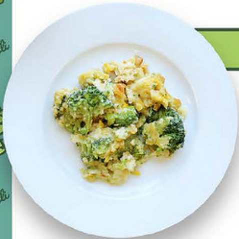

- Frozen broccoli
- Cream-style corn
- Egg
- Margarine
- Saltine crackers

Macaroni and Cheese with Broccoli

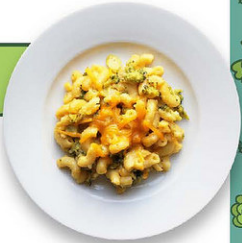

- Frozen broccoli
- Elbow macaroni
- Flour
- Milk (low-fat)
- Cheddar cheese (low-fat)
- Pepper

Find these broccoli recipes here:
https://choosemyplate.gov/5-ways-series

What's Cooking? USDA Mixing Bowl

American Heart Association. life is why™ | Healthy For Good™

When you hear "salad," you may think of a boring bowl of lettuce and tomatoes. But salad can be so much more! With a few simple additions, it can be a delicious, healthy and inexpensive meal.

BUILD A HEALTHIER SALAD

FRUITS

Slice up fresh fruit that's in season or on sale – choose a rainbow of colors! Pairing sweet fruits like pear, apple or pomegranate with savory vinaigrettes will bring complexity and flavor to any salad. Dried fruits without added sugars are another super salad ingredient.

GRAINS

Warm or cold cooked whole grains add bulk and satisfaction. Try whole wheat couscous, barley, quinoa, bulgur or wild rice. To save money, look for whole grains in the bulk aisle of your grocery store. Whole wheat pasta is another inexpensive way to bulk up any basic salad.

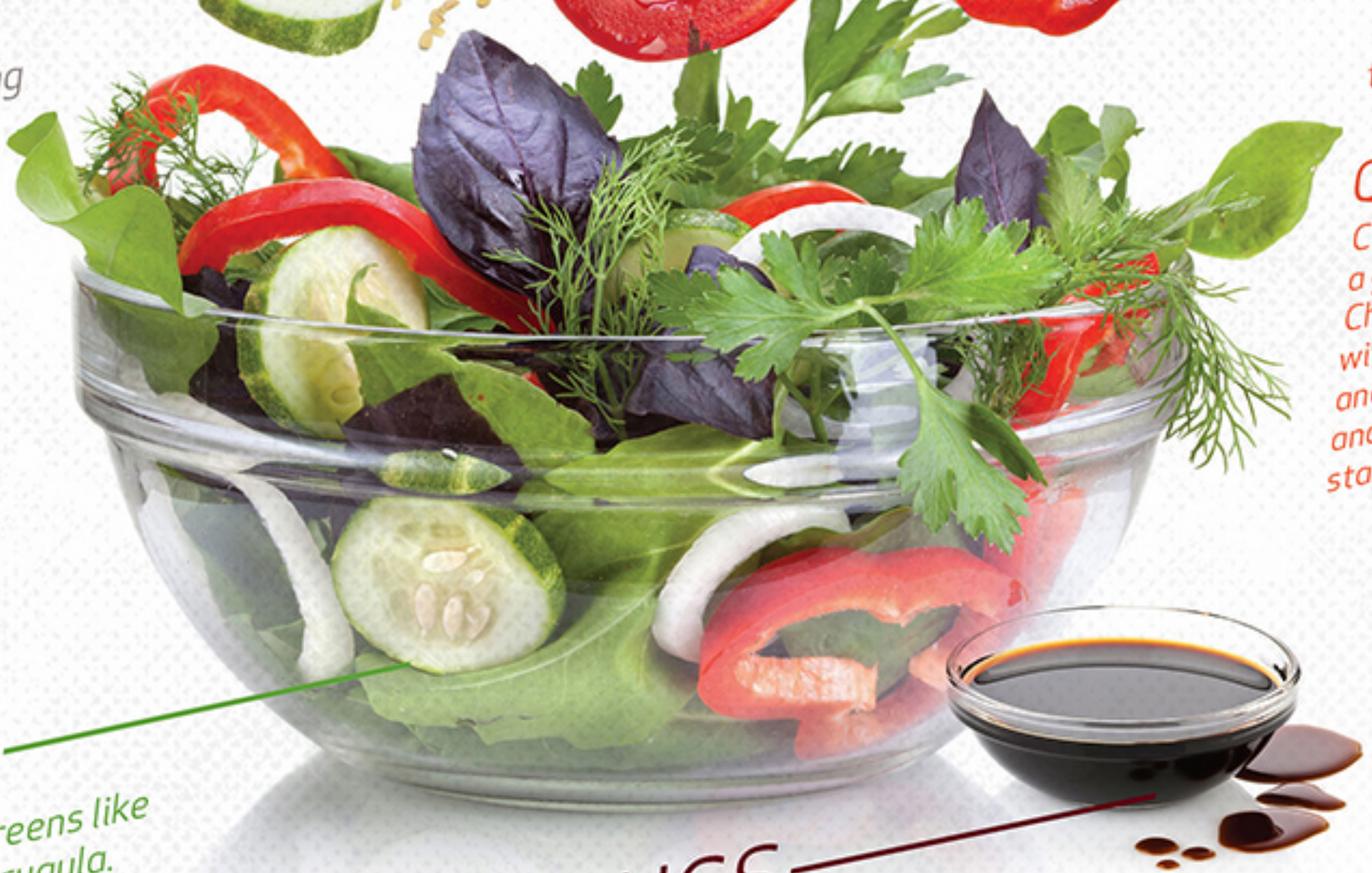

PROTEINS

Add more satisfaction to your salad with skinless poultry like grilled chicken breast or fish like salmon and tuna, which provide omega-3 fatty acids. Mix in a chopped hard-boiled egg or a small amount of cheese. Toss in some chickpeas, kidney, navy or black beans. Unsalted nuts, like peanuts, almonds and walnuts, give your salad extra crunch and a dose of healthy fat. All these protein foods will keep you feeling fuller longer.

QUICK TIP

Choose lower-fat, lower-sodium cheeses such as mozzarella or Swiss.

VEGGIES

Raw vegetables like carrots, cucumbers, broccoli and cauliflower add great crunch and color. Roasted veggies like beets, potatoes and squash add terrific flavor anda little bit of sweetness to any main meal salad.

QUICK TIP

Canned and frozen produce are a great addition to any salad. Check labels and choose the options with the lowest amounts of salt and added sugars. Drain, rinse and pat dry to help your greens stay crisp.

GREENS

Choose dark, leafy greens like romaine, spinach or arugula. Add color with radicchio, red leaf lettuce or red cabbage. If you have fresh herbs, like basil, thyme, oregano or mint, they add zest and extra nutrients to your salad bowl.

DRESSINGS

With oil, vinegar and spices in your pantry, you are minutes away from a simple, healthy homemade vinaigrette dressing. Jazz it up with tasty ingredients like chopped fresh herbs, a squeeze of citrus juice and diced veggies with lots of flavor, like onions, garlic and scallions. Experiment by adding small amounts of those add-ins to this vinaigrette recipe, taste-testing as you go.

VINAIGRETTE RECIPE

Whisk together 1/4 cup olive oil, 1/4 cup balsamic vinegar and 1/4 teaspoon ground mustard. Add a dash or two of black pepper and toss into salad (serves four).

heart.org/addcolor
#ADDCOLOR

+color™

USDA
United States Department of Agriculture

10 tips
Nutrition
Education Series

MyPlate MyWins

Based on the
Dietary
Guidelines
for Americans

Focus on fruits

Eating fruit provides health benefits. People who eat more vegetables and fruits as part of an overall healthy eating style are likely to have a reduced risk of some chronic diseases. Fruits provide nutrients vital for health, such as potassium, dietary fiber, vitamin C, and folate. Focus on whole fruits—fresh, canned, frozen, or dried—instead of juice. The sugar naturally found in fruit does not count as added sugar.

1 Keep visible reminders

Keep a bowl of whole fruit on the table, counter, or in the refrigerator.

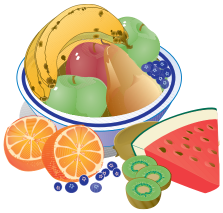

2 Experiment with flavor

Buy fresh fruits in season when they may be less expensive and at their peak flavor. Use fruits to sweeten a recipe instead of adding sugar.

3 Think about variety

Buy fruits that are dried, frozen, and canned (in water or 100% juice) as well as fresh, so that you always have a supply on hand.

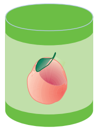

4 Don't forget the fiber

Make most of your choices whole or cut-up fruit, rather than juice, for the benefits that dietary fiber provides.

5 Include fruit at breakfast

At breakfast, top your cereal with bananas, peaches, or strawberries; add blueberries to pancakes; drink 100% orange or grapefruit juice. Or, try a fruit mixed with fat-free or low-fat yogurt.

6 Try fruit at lunch

At lunch, pack a tangerine, banana, or grapes to eat or choose fruits from a salad bar. Individual containers of fruits like peaches or applesauce are easy to carry and convenient for lunch.

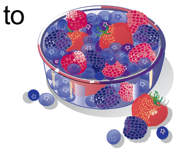

7 Enjoy fruit at dinner, too

At dinner, add crushed pineapple to coleslaw or include orange sections, dried cranberries, or grapes in a tossed salad. Try fruit salsa on top of fish.

8 Snack on fruits

Fruits make great snacks. Try dried fruits mixed with nuts or whole fruits like apples. They are easy to carry and store well.

9 Be a good role model

Set a good example for children by eating fruit every day with meals or as snacks.

10 Keep fruits safe

Rinse fruits before preparing or eating them. Under clean, running water, rub fruits briskly to remove dirt and surface microorganisms. After rinsing, dry with a clean towel.

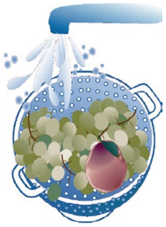

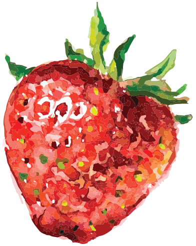

STRAWBERRY

WHAT IS IT?

Available fresh in the spring and summer, strawberries are a fruit grown on flowering plants in the rose family.

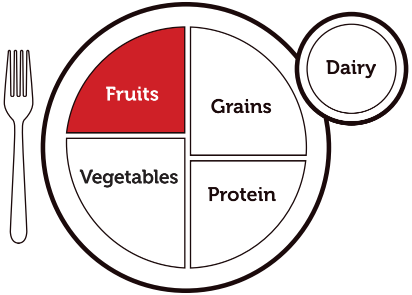

FOCUS ON WHOLE FRUITS

Strawberries are a lower-calorie sweet treat that can be easily consumed fresh or frozen in a variety of snacks and meals.

HOW IT FITS INTO MYPLATE

A 2,000 calorie diet has a daily Fruit Group target of 2 cups. Snacking on strawberries adds up quickly toward your goal!

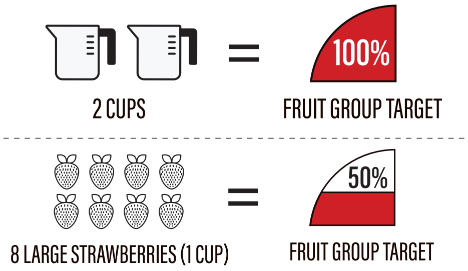

To find your food group targets, go to **ChooseMyPlate.gov/Checklist**

FUN FACTS & TIPS

- Strawberries were first commercially grown in America in the 1800s.
- 90% Approximately 90% of America's strawberries are produced in California.
- For a fun family outing, visit a farm that offers pick-your-own strawberries.
- Blend fat-free or low-fat yogurt with fresh or frozen strawberries for a sweet smoothie.

MyPlate MyWins

For more information go to **ChooseMyPlate.gov**
USDA is an equal opportunity provider, employer, and lender.

USDA

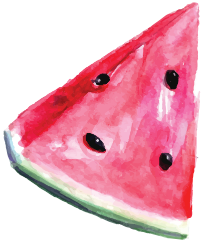

WATERMELON

WHAT IS IT?

Commercially grown in 44 different states, watermelon is a flowering plant that grows in the summer months in the U.S.

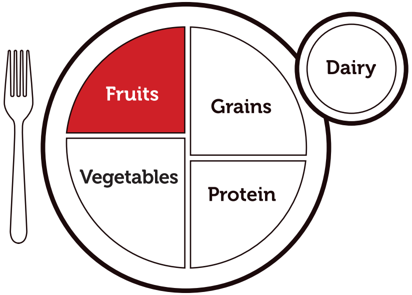

FOCUS ON WHOLE FRUITS

Watermelon is one of the most affordable fruits available and is a simple, sweet way to add fruit to your plate.

HOW IT FITS INTO MYPLATE

A 2,000 calorie diet has a daily Fruit Group target of 2 cups. By eating just 1 medium watermelon wedge, you'll already meet your goal!

1 MEDIUM WEDGE = 2 CUPS

2 CUPS = 100% FRUIT GROUP TARGET

To find your food group targets, go to **ChooseMyPlate.gov/Checklist**

FUN FACTS & TIPS

USDA developed the classic variety, called Charleston Grey, in 1954.

66% — Texas, Florida, Georgia & California grow 66% of the watermelon in the U.S.

Cool off! Freeze slices of watermelon into "popsicles" for an icy cold sweet treat.

canned Peaches 5 ways

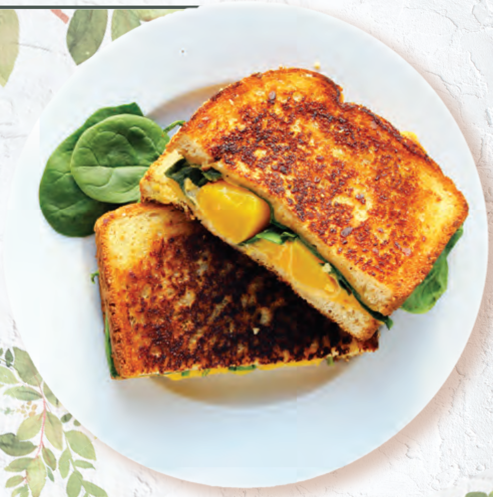

Grilled Cheese with Peaches

+ Canned peaches
+ Whole grain bread
+ Cheddar cheese [low-fat]
+ Spinach

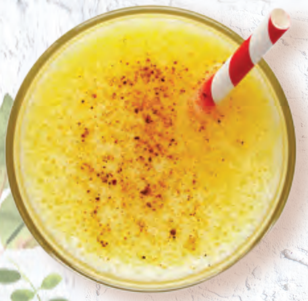

Peach Cooler

+ Canned peaches
+ Milk [low-fat]
+ Lemon juice
+ Nutmeg

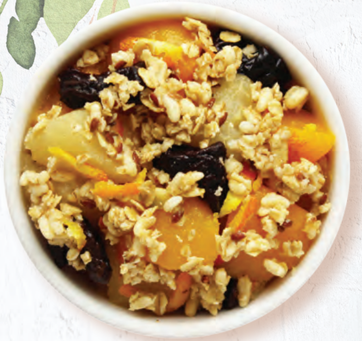

Rise & Shine Cobbler

+ Canned peaches
+ Canned pears
+ Prunes
+ Vanilla extract
+ Orange
+ Granola

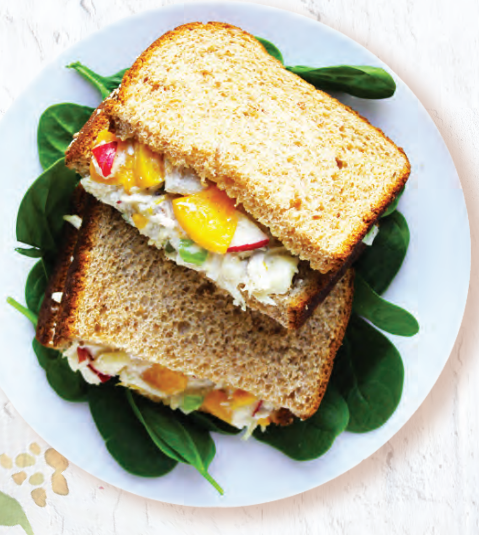

Chicken Salad & Peach Sandwich

+ Canned peaches
+ Whole grain bread
+ Cooked chicken
+ Celery stalk
+ Apples
+ Mayo [nonfat]
+ Walnuts

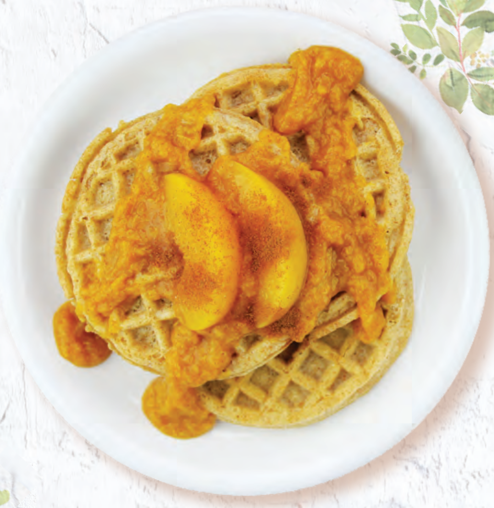

Peach Sauce

+ Canned peaches
+ Cinnamon

Find these peach recipes here:
https://choosemyplate.gov/5-ways-series

What's Cooking? USDA MIXING BOWL

BERRIES 5 WAYS

Overnight Oats & Berries

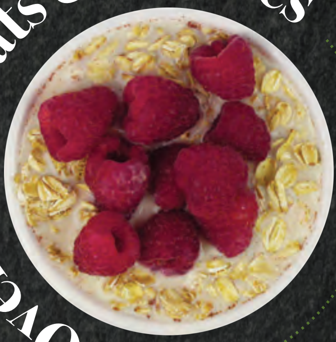

WHAT YOU'LL NEED:

+ Frozen raspberries
+ Lowfat milk
+ Greek yogurt (lowfat)
+ Uncooked rolled oats
+ Honey
+ Vanilla extract
+ Cinnamon

Broccoli Strawberry Orzo Salad

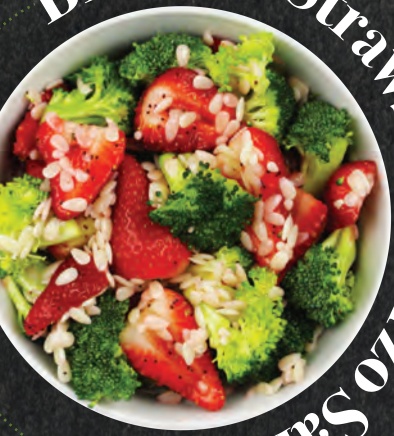

WHAT YOU'LL NEED:

+ Orzo pasta
+ Broccoli
+ Strawberries
+ Sunflower seeds
+ Poppy seeds
+ Lemon juice
+ Apple cider vinegar
+ Olive oil
+ Sugar

Fabulous Fruit Muffins

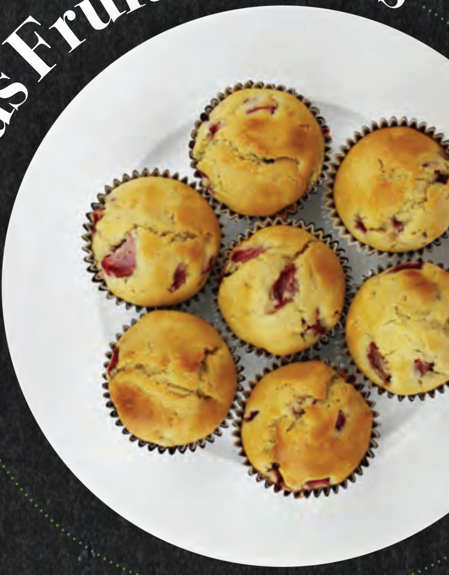

WHAT YOU'LL NEED:

+ Flour
+ Sugar
+ Baking powder
+ Buttermilk (lowfat)
+ Margarine
+ Egg
+ Vanilla extract
+ Frozen strawberries

Spicy Fruit Cup

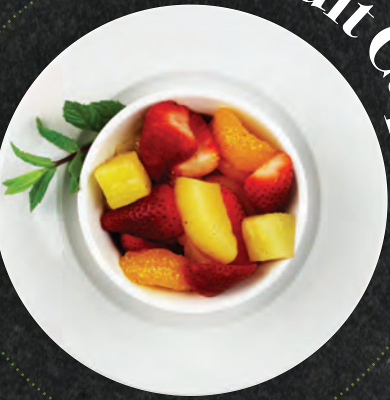

WHAT YOU'LL NEED:

+ Strawberries
+ Mandarin oranges
+ Pears
+ Orange juice
+ Pineapple chunks
+ Cinnamon
+ Nutmeg

Very Berry Smoothie

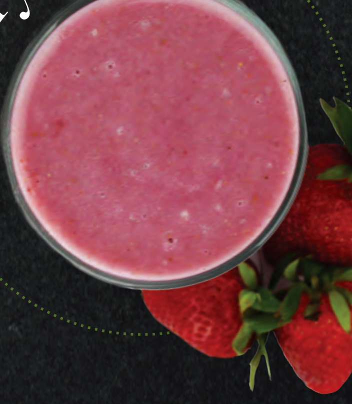

WHAT YOU'LL NEED:

+ Frozen strawberries
+ Frozen raspberries
+ Banana
+ Milk (lowfat)
+ Ice

Find these berry recipes here: **https://go.usa.gov/x59VS**
For more recipes go to: **www.whatscooking.fns.usda.gov**

What's Cooking? USDA MIXING BOWL

American Heart Association. life is why™ | Healthy For Good™

The best way to get all of the vitamins, minerals and nutrients you need is to eat a variety of colorful fruits and veggies. Add color to your plate each day with the five main color groups.

RED & PINK

beets
cherries
cranberries
pink grapefruit
pomegranates
radicchio
red radishes
raspberries
red apples
red grapes
red peppers
red potatoes
rhubarbs
strawberries
tomatoes
watermelons

BLUE & PURPLE

blackberries
blueberries
eggplants
grapes
plums
prunes
purple figs
purple onions
radicchio
red cabbage
red onions

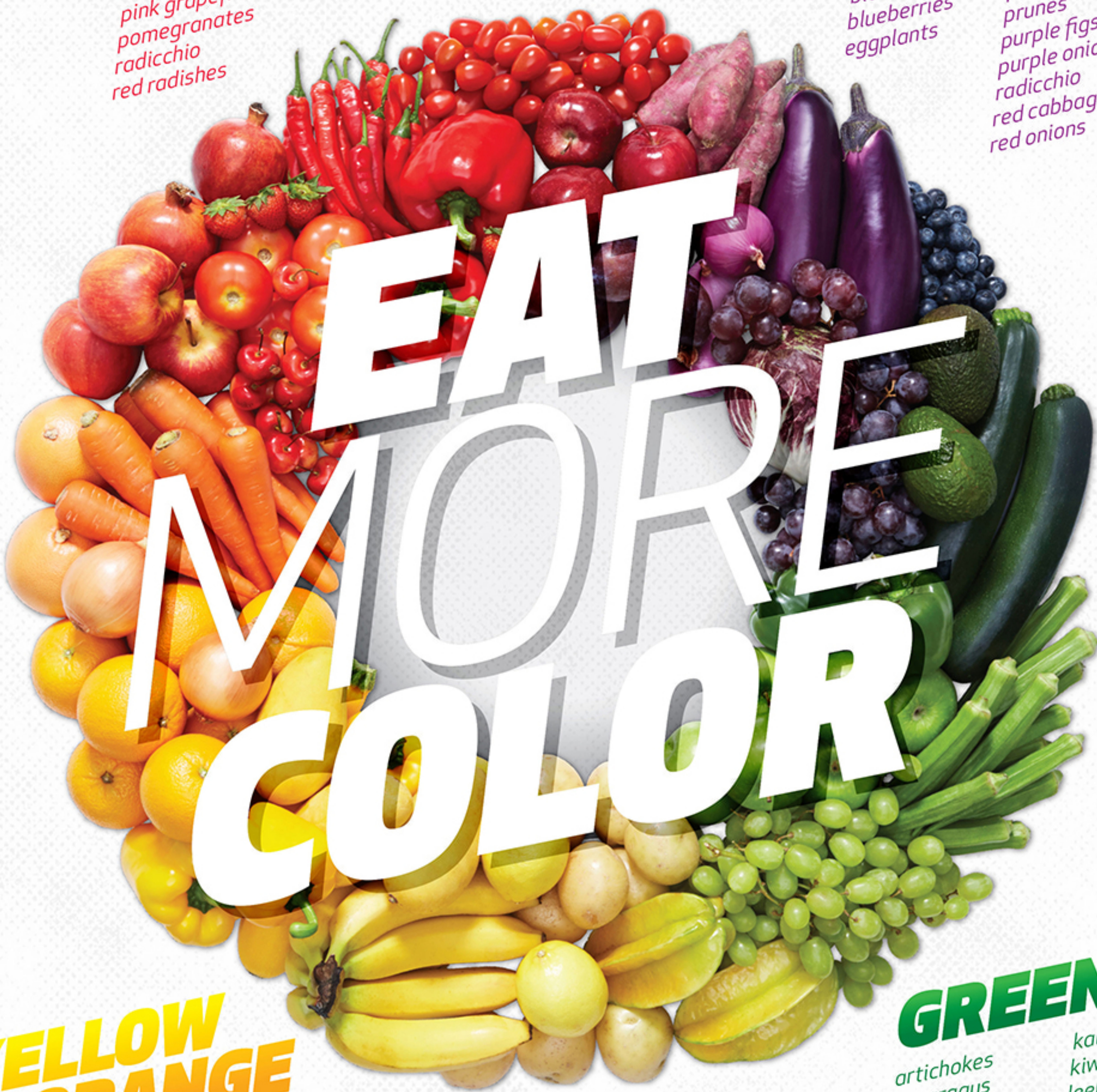

YELLOW & ORANGE

acorn squash
butternut squash
apricots
cantaloupes
carrots
corn
grapefruit
lemons
mangoes
nectarines
oranges
orange peppers
papayas
peaches
pineapples
pumpkins
summer squash
sweet potatoes
tangerines
yams
yellow apples
yellow peppers
yellow squash

WHITE & BROWN

bananas
brown pears
cauliflower
currants
dates
garlic
Jerusalem artichokes
mushrooms
onions
potatoes
parsnips
raisins
shallots
turnips

GREEN

artichokes
asparagus
avocados
bok choy
broccoli
Brussels sprouts
celery
collard greens
cucumbers
green beans
green cabbage
green grapes
green onions
green peppers
kale
kiwis
leeks
limes
mustard greens
okra
pears
peas
romaine lettuce
snow peas
spinach
sugar snap peas
watercress
zucchini

heart.org/addcolor
#ADDCOLOR

+color™

10 tips
Nutrition Education Series

be active adults

10 tips to help adults include physical activity into their lifestyle

ChooseMyPlate.gov

Being physically active is important for your health. Adults who are physically active are less likely to develop some chronic diseases than adults who are inactive. Physical activity is any form of exercise or movement of the body that uses energy. People of all ages, shapes, sizes, and abilities can benefit from a physically active lifestyle.

1 start activities slowly and build up over time

If you are just starting physical activity, build up slowly. This will help to prevent injury. After a few weeks, increase how often and how long you are active.

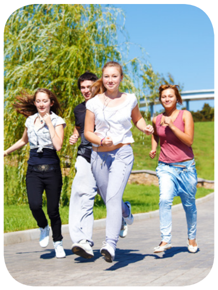

2 get your heart pumping

For health benefits, do at least 2½ hours each week of physical activity that requires moderate effort. A few examples include brisk walking, biking, swimming, and skating. Spread activities over the week, but do them at least 10 minutes at a time.

3 strength-train for healthy muscles and bones

Do strengthening activities twice a week. Activities that build strength include lifting weights, doing push-ups and sit-ups, working with resistance bands, or heavy gardening.

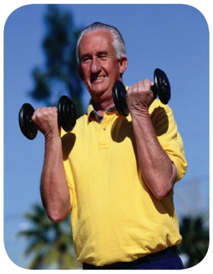

4 make active choices throughout the day

Every little bit of activity can add up and doing something is better than nothing. Take the stairs instead of the elevator, go for a 10-minute walk on your lunch break, or park further away from work and walk.

5 be active your way

Mix it up—there are endless ways to be active. They include walking, biking, dancing, martial arts, gardening, and playing ball. Try out different activities to see what you like best and to add variety.

6 use the buddy system

Activities with friends or family are more enjoyable than doing them alone. Join a walking group, attend fitness classes at a gym, or play with the kids outside. Build a support network—your buddies will encourage you to keep being active.

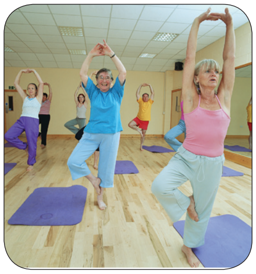

7 set goals and track your progress

Plan your physical activity ahead of time and keep records. It's a great way to meet your goals. Track your activities with the Physical Activity Tracker on **SuperTracker**.* Use the My Journal feature to record what you enjoyed so you can build a plan that is right for you.

8 add on to your active time

Once you get used to regular physical activity, try to increase your weekly active time. The more time you spend being physically active, the more health benefits you will receive.

9 increase your effort

Add more intense activities once you have been moderately active for a while. You can do this by turning a brisk walk into a jog, swimming or biking faster, playing soccer, and participating in aerobic dance.

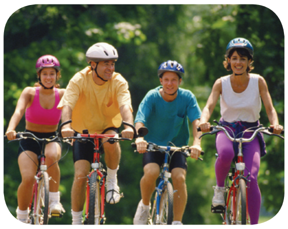

10 have fun!

Physical activity shouldn't be a chore. It can help you feel better about yourself and the way you live your life. Choose activities that you enjoy and that fit your lifestyle.

*Find the SuperTracker at https://www.supertracker.usda.gov.

10 tips
Nutrition Education Series

physical activity at home, work, and play

ChooseMyPlate.gov

10 tips to make physical activity a regular part of the day

Adding activity into your day is possible. Choose activities that you enjoy. Adults should aim for at least 2½ hours or 150 minutes of physical activity each week. Every little bit adds up, and doing something is better than doing nothing. Most important—have fun while being active!

1 take 10

Do at least 10 minutes of activity at a time to reach your weekly goal. Walk the dog for 10 minutes before and after work and add a 10-minute walk at lunchtime.

2 mix it up

Start the week with a swim at the pool, take a yoga class during a weekday lunch, lift weights in the evening, and end the week by working in the garden.

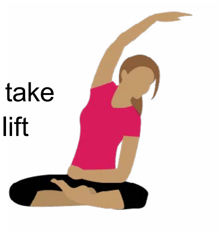

3 be ready anytime

Keep comfortable clothes and walking or running shoes in the car and at the office.

4 find ways to move

Take a brisk walk around the parking lot, jog to the bus stop, or ride your bike to the subway station. If you have an infant or toddler, take a long walk using the stroller and everyone gets some fresh air.

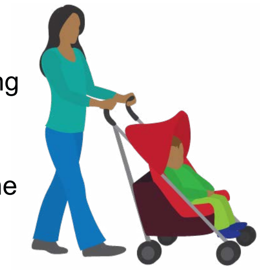

5 work out during TV time

Watch a movie while you jog on a treadmill or download a video on your phone and watch while you ride a stationary bike.

6 be an active parent

Instead of standing on the sidelines, walk up and down the soccer, football, or softball field while the kids play their game.

7 find support

Join a walking group, play wheelchair sports, practice martial arts, or sign up for an exercise class in your community. Recruit family or friends for support.

8 enjoy the great outdoors

Tumble in the leaves, build a snowman with your kids, or ski cross-country. Visit a county or national park and spend time hiking, canoeing, or boating.

9 look for wellness at work

Find a softball, basketball, or volleyball team at your job. You can also take the lead by starting a wellness or exercise group in your office.

10 the chores count, too!

Clean the house, wash the car, or mow the lawn with a push mower. Know that these activities count toward your goal of at least 150 minutes each week.

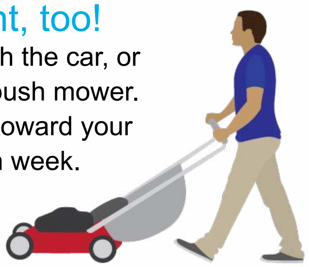

healthy eating for an active lifestyle

10 tips for combining good nutrition and physical activity

For youth and adults engaging in physical activity and sports, healthy eating is essential for optimizing performance. Combining good nutrition with physical activity can lead to a healthier lifestyle.

1 maximize with nutrient-packed foods

Give your body the nutrients it needs by eating a variety of nutrient-packed food, including whole grains, lean protein, fruits and vegetables, and low-fat or fat-free dairy. Eat less food high in solid fats, added sugars, and sodium (salt).

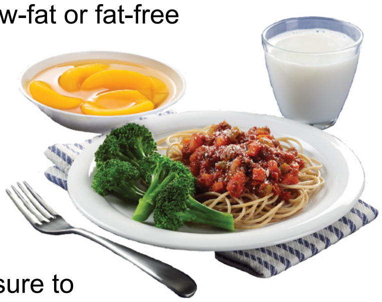

2 energize with grains

Your body's quickest energy source comes from foods such as bread, pasta, oatmeal, cereals, and tortillas. Be sure to make at least half of your grain food choices whole-grain foods like whole-wheat bread or pasta and brown rice.

3 power up with protein

Protein is essential for building and repairing muscle. Choose lean or low-fat cuts of beef or pork, and skinless chicken or turkey. Get your protein from seafood twice a week. Quality protein sources come from plant-based foods, too.

4 mix it up with plant protein foods

Variety is great! Choose beans and peas (kidney, pinto, black, or white beans; split peas; chickpeas; hummus), soy products (tofu, tempeh, veggie burgers), and unsalted nuts and seeds.

5 vary your fruits and vegetables

Get the nutrients your body needs by eating a variety of colors, in various ways. Try blue, red, or black berries; red and yellow peppers; and dark greens like spinach and kale. Choose fresh, frozen, low-sodium canned, dried, or 100 percent juice options.

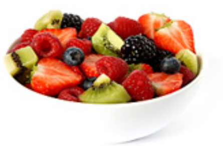

6 don't forget dairy

Foods like fat-free and low-fat milk, cheese, yogurt, and fortified soy beverages (soymilk) help to build and maintain strong bones needed for everyday activities.

7 balance your meals

Use MyPlate as a reminder to include all food groups each day. Learn more at www.ChooseMyPlate.gov.

8 drink water

Stay hydrated by drinking water instead of sugary drinks. Keep a reusable water bottle with you to always have water on hand.

9 know how much to eat

Get personalized nutrition information based on your age, gender, height, weight, current physical activity level, and other factors. Use SuperTracker to determine your calorie needs, plan a diet that's right for you, and track progress toward your goals. Lean more at www.SuperTracker.usda.gov.

10 reach your goals

Earn Presidential recognition for reaching your healthy eating and physical activity goals. Log on to www.presidentschallenge.org to sign up for the Presidential Active Lifestyle Award (PALA+).

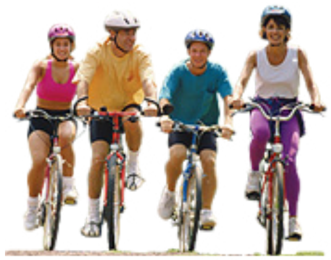

Go to www.ChooseMyPlate.gov and www.Fitness.gov for more information.

DG TipSheet No. 25
March 2013
Center for Nutrition Policy and Promotion
USDA is an equal opportunity provider and employer.

American Heart Association® life is why™ | Healthy For Good™

MAKE EVERY MOVE COUNT

When you Move More every day, you can reach some pretty big goals over time. With motivation, dedication and great foot support, your small daily steps can add up to huge strides. Let your feet take you places you never thought possible!

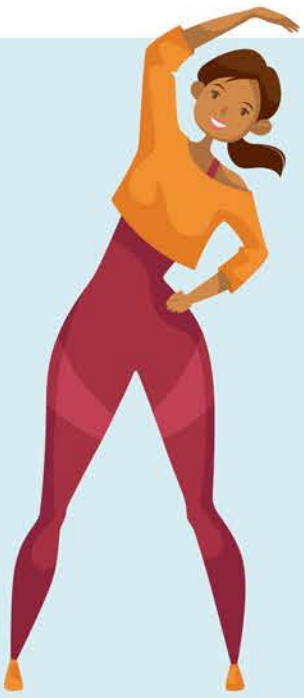

10 Minutes

of stretching is like walking the length of a football field

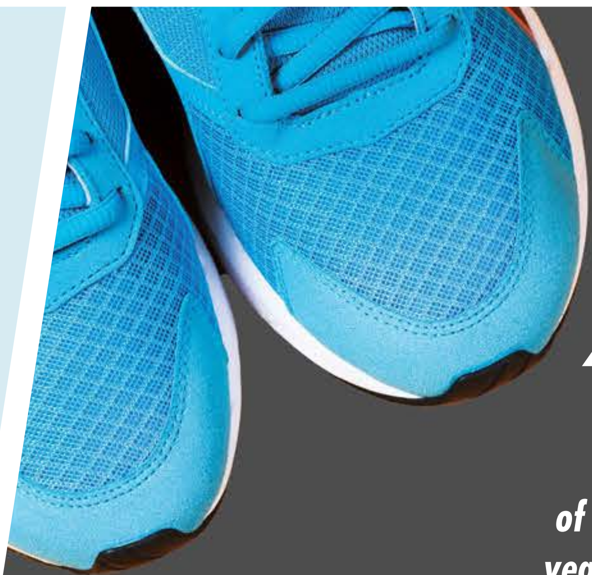

2.5 Hours

of walking every week for a year is like walking across the state of Wyoming

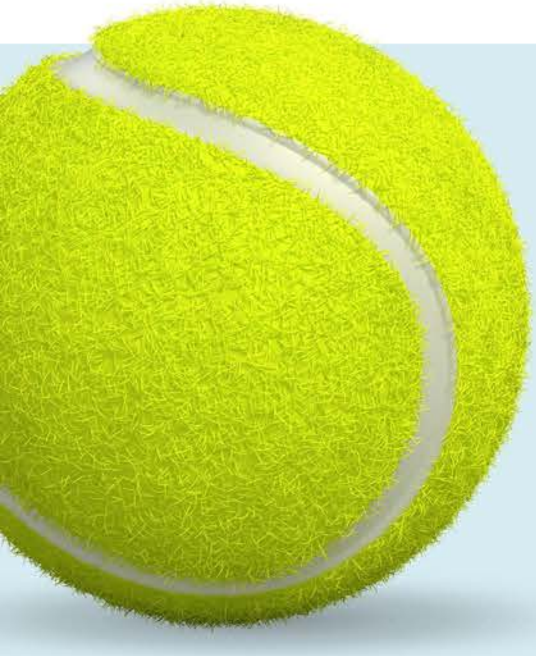

30 Minutes

of singles tennis is like walking a 5K

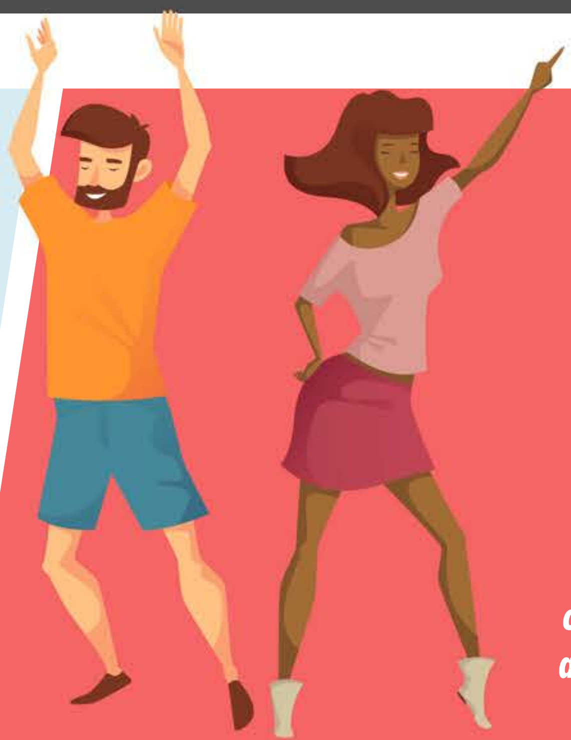

1 Hour

of dancing every week for a year is like walking from Chicago to Indianapolis

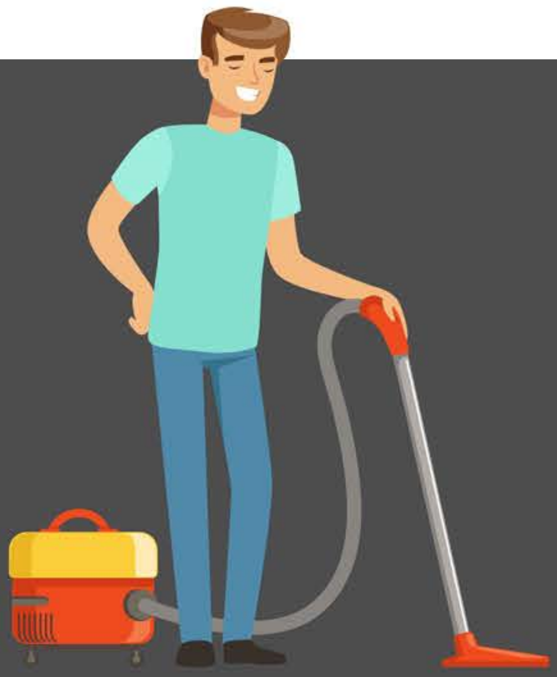

20 Minutes

of vacuuming is like walking one mile

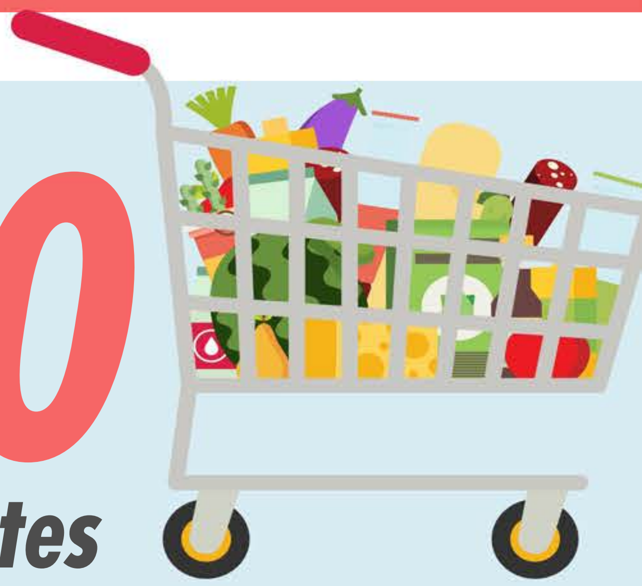

30 Minutes

of grocery shopping every other week for a year is like walking a marathon

EAT SMART ADD COLOR MOVE MORE BE WELL

Source: http://www.purdue.edu/walktothemoon/activities.html

FOR MORE WAYS TO ADD ACTIVITY TO YOUR LIFE, VISIT

HEART.ORG/MOVEMORE

Nationally supported by Dr. Scholl's®

American Heart Association | American Stroke Association
life is why™

The American Heart Association Recommendations for Physical Activity in Adults

For Overall Cardiovascular Health:

At least **30** minutes of *moderate-intensity* aerobic activity at least **5** days *per week* for a total of **150** minutes

OR

At least **25** minutes of *vigorous* aerobic activity at least **3** days *per week* for a total of **75** minutes

or a combination of the two

AND

Moderate to **HIGH INTENSITY** muscle-strengthening activity at least **2** days *per week* for additional health benefits

For Lowering Blood Pressure and Cholesterol:

An average of **40** minutes of *moderate- to vigorous-intensity* aerobic activity **3** or **4** days *per week*

© 2015

Learn more at **heart.org/ActivityRecommendations**.

American Heart Association. | Healthy For Good™

CLEAN UP YOUR SLEEP HYGIENE

Is your phone keeping you up at night? With a few tweaks to your tech habits, you can wake up more refreshed and ready to face the day.

MOVE IT

Charge your device as far away from your bed as possible. Added bonus? The distance may help you feel less overwhelmed in general.

DIM IT

Dim your screen or use a red filter app at night. The bright blue light of most devices can mess with your circadian rhythm and melatonin production.

SET IT

Alarms aren't just for waking up – set a bedtime alarm to remind you that it's time to wrap it up for the night.

LOCK IT

If you've got a scrolling habit you need to kick, try an app-blocking app that makes it impossible to get lost in after-hours emails, social media or gaming.

BLOCK IT

Tell notifications to buzz off if they're waking you up at night. Put your phone on "do not disturb" mode to block it all out when you're trying to sleep.

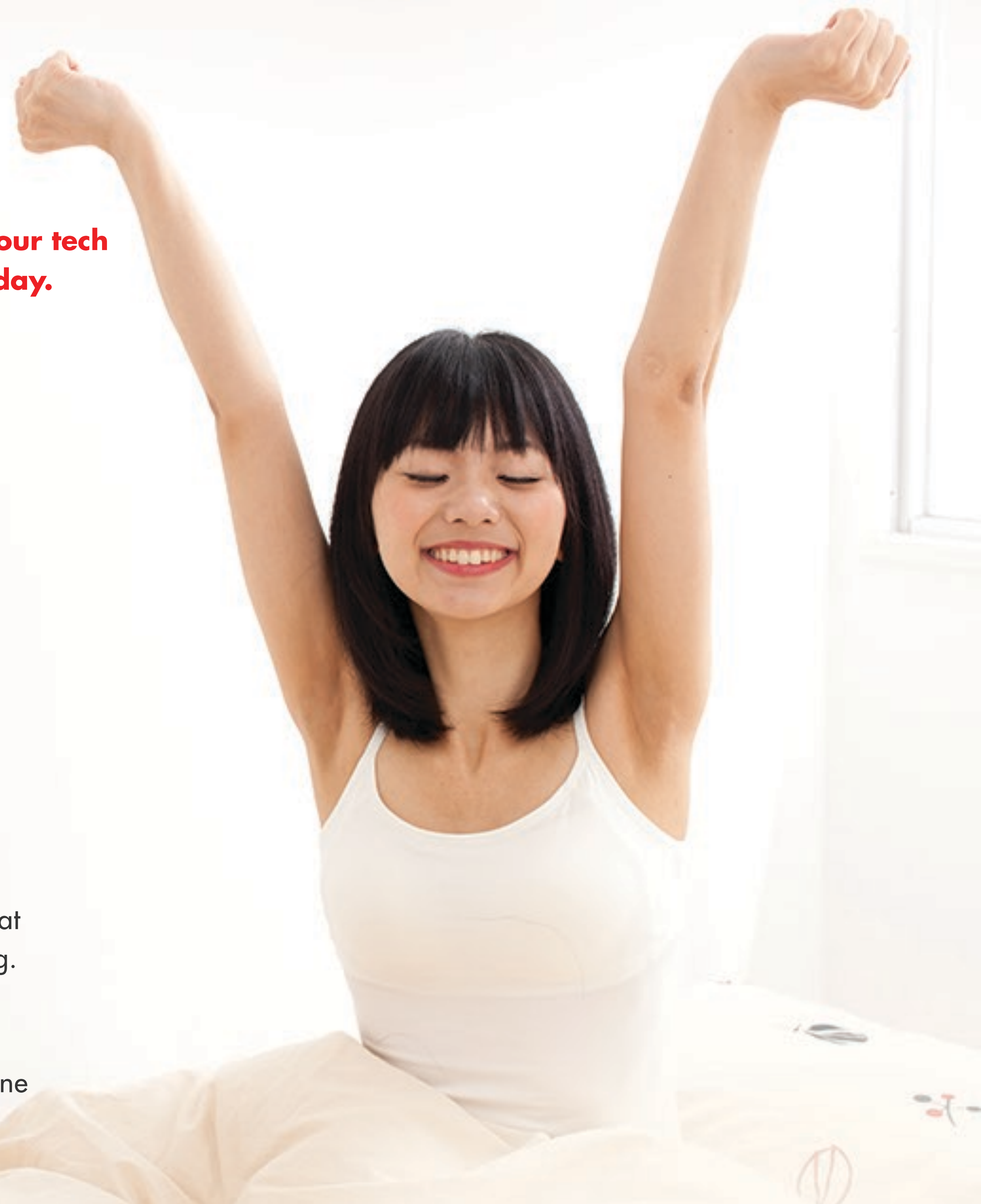

GET MORE WELLNESS TIPS AT
HEART.ORG/HEALTHYFORGOOD

©American Heart Association 2018

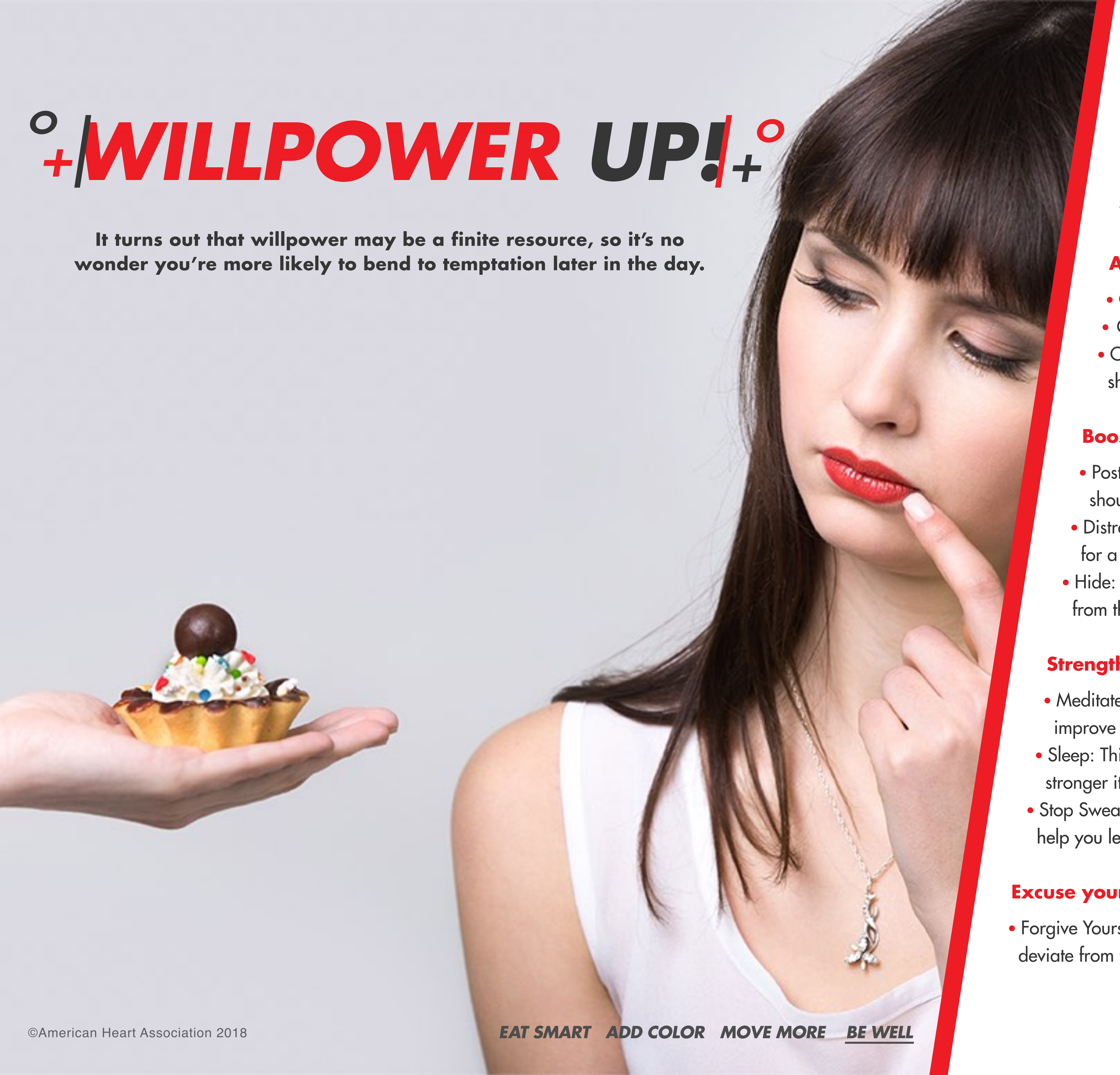

American Heart Association | Healthy For Good™

WILLPOWER UP!

It turns out that willpower may be a finite resource, so it's no wonder you're more likely to bend to temptation later in the day.

Try a few of these tips to build up your willpower so you can keep positive habits going strong.

Arrange your environment.

- Clean: Get rid of your temptations, so you don't have to resist them.
- Commit: Make plans with a friend who can hold you accountable.
- Optimize: Time tasks so they align with your willpower – like grocery shopping when you're already full.

Boost your willpower in the moment.

- Postpone: Say "not now, maybe later" to get the devil off your shoulder when you're tempted.
- Distract: Give your impulse a chill pill by focusing on something else for a few minutes.
- Hide: Remove the temptation from plain sight or remove yourself from the situation.

Strengthen your ongoing willpower.

- Meditate: a 10-minute mindful meditation sesh every morning can help improve impulse control over time.
- Sleep: Think of rest as a shield from temptations – the more you sleep, the stronger it gets.
- Stop Swearing: Changing a speech pattern, like avoiding expletives, can help you learn to extend your willpower.

Excuse your setbacks.

- Forgive Yourself: You're human, after all! Give yourself a break when you deviate from the path, and you'll be more likely to get back on it.

©American Heart Association 2018

GET MORE WELLNESS TIPS AT
HEART.ORG/HEALTHYFORGOOD

USDA

United States Department of Agriculture

10 tips
Nutrition Education Series

MyPlate MyWins

Based on the **Dietary Guidelines for Americans**

Choosing whole-grain foods

Whole grains are important sources of nutrients such as zinc, magnesium, B vitamins, and fiber. There are many choices available to make half your grains whole grains. But whole-grain foods should be handled with care. Over time and if not properly stored, oils in whole grains can cause spoilage. Consider these tips to select whole-grain products and keep them fresh to eat.

1 Search the label

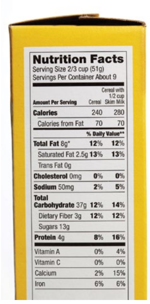

Whole grains can be an easy choice when preparing meals. Choose whole-grain breads, breakfast cereals, and pastas. Look at the Nutrition Facts labels and ingredients lists to find choices lower in sodium, saturated fat, and added sugars.

2 Look for the word "whole" at the beginning of the ingredients list

Some whole-grain ingredients include whole oats, whole-wheat flour, whole-grain corn, whole-grain brown rice, and whole rye. Foods that say "multi-grain," "100% wheat," "high fiber," or are brown in color may not be a whole-grain product.

3 Choose whole grains at school

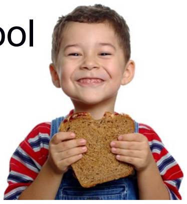

Prepare meals and snacks with whole grains at home so your kids are more likely to choose whole-grain foods at school.

4 Find the fiber on label

If the product provides at least 3 grams of fiber per serving, it is a good source of fiber. If it contains 5 or more grams of fiber per serving, it is an excellent source of fiber.

5 Is gluten in whole grains?

People who can't eat wheat gluten can eat whole grains if they choose carefully. There are many whole-grain products, such as buckwheat, certified gluten-free oats or oatmeal, popcorn, brown rice, wild rice, and quinoa that fit gluten-free diet needs.

6 Check for freshness

Buy whole-grain products that are tightly packaged and well sealed. Grains should always look and smell fresh. Also, check the expiration date and storage guidelines on the package.

7 Keep a lid on it

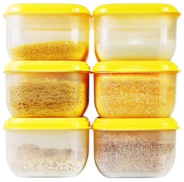

When storing whole grains from bulk bins, use containers with tight-fitting lids and keep in a cool, dry location. A sealed container is important for maintaining freshness and reducing bug infestations.

8 Buy what you need

Purchase smaller quantities of whole-grain products to reduce spoilage. Most grains in sealed packaging can be kept in the freezer.

9 Wrap it up

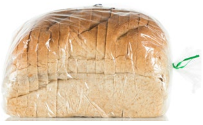

Whole-grain bread is best stored at room temperature in its original packaging, tightly closed with a quick-lock or twist tie. The refrigerator will cause bread to lose moisture quickly and become stale. Properly wrapped bread will store well in the freezer.

10 What's the shelf life?

Since the oil in various whole-grain flours differs, the shelf life varies too. Most whole-grain flours keep well in the refrigerator for 2 to 3 months and in the freezer for 6 to 8 months. Cooked brown rice can be refrigerated 3 to 5 days and can be frozen up to 6 months.

Center for Nutrition Policy and Promotion
USDA is an equal opportunity provider, employer, and lender.

Go to ChooseMyPlate.gov for more information.

DG TipSheet No. 22
September 2012
Revised October 2016

United States Department of Agriculture

10 tips Nutrition Education Series

MyPlate MyWins

Based on the **Dietary Guidelines for Americans**

Make half your grains whole grains

Any food made from wheat, rice, oats, cornmeal, barley, or another cereal grain is a grain product. Grains are divided into two subgroups, whole grains and refined grains. Whole grains contain the entire grain kernel—the bran, germ, and endosperm. People who eat whole grains as part of a healthy eating style have a reduced risk of some chronic diseases.

1 Make simple shifts

To make half your grains whole grains, choose 100% whole-wheat bread, bagels, pasta, or tortillas; brown rice; oatmeal; or grits.

2 Whole grains can be healthy snacks

Popcorn is a whole grain. Make it with little or no added salt or butter. Also, try 100% whole-wheat or rye crackers.

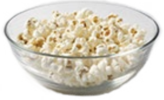

3 Save some time

Cook extra brown rice or oatmeal when you have time. Refrigerate half of what you cook to heat and serve later in the week.

4 Mix it up with whole grains

Use whole grains in mixed dishes, such as barley in vegetable soups or stews and bulgur wheat in casseroles or stir-fries. Try a quinoa salad or pilaf.

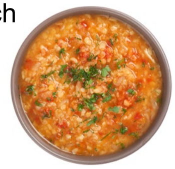

5 Try whole-wheat versions

Change up your favorite meal with whole grains. Try brown rice stuffing in baked green peppers or tomatoes, and whole-wheat noodles in lasagna.

6 Bake up some whole-grain goodness

Experiment by substituting buckwheat, millet, or oat flour for up to half of the flour in your favorite pancake or waffle recipes. To limit saturated fat and added sugars, top with fruit instead of butter and syrup.

7 Be a good role model for children

Set a good example for children by serving and eating whole grains every day with meals or as snacks.

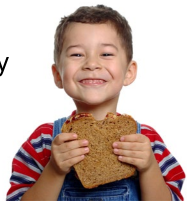

8 Check the label

Most refined grains are enriched. This means that certain B vitamins and iron are added back after processing. Check the ingredients list to make sure the word “enriched” is included in the grain name.

9 Know what to look for on the ingredients list

Read the ingredients list and choose products that name a whole-grain ingredient ***first*** on the list. Look for “whole wheat,” “brown rice,” “bulgur,” “buckwheat,” “oatmeal,” “whole-grain cornmeal,” “whole oats,” or “whole rye.”

10 Be a smart shopper

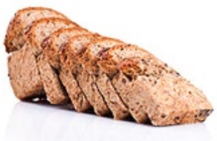

The color of a food is not an indication that it is a whole-grain food. Foods labeled as “multi-grain,” “stone-ground,” “100% wheat,” “cracked wheat,” “seven-grain,” or “bran” are usually not 100% whole-grain products, and may not contain **any** whole grain.

Center for Nutrition Policy and Promotion
USDA is an equal opportunity provider, employer, and lender.

Go to ChooseMyPlate.gov for more information.

DG TipSheet No. 4
June 2011
Revised October 2016

What's Cooking? USDA MIXING BOWL

BROWN RICE 5 WAYS

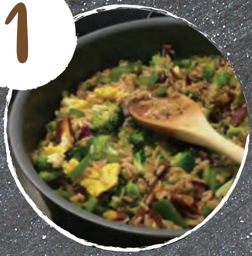

1 Fried Rice

+ Brown rice
+ Carrot
+ Bell pepper
+ Onion
+ Broccoli
+ Soy sauce
+ Vegetable oil
+ Eggs
+ Chicken
+ Spices to taste

2 Caribbean Casserole

+ Brown rice
+ Onion
+ Stewed tomatoes
+ Green pepper
+ Black beans
+ Oregano leaves
+ Canola oil
+ Spices to taste

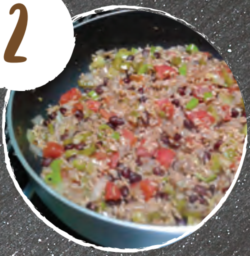

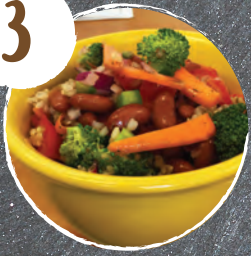

3 Fiesta Rice Salad

+ Brown rice
+ Broccoli
+ Carrots
+ Red onion
+ Tomatoes
+ Green pepper
+ Kidney beans
+ Cilantro
+ Spices to taste

4 Cheesy Broccoli Rice Squares

+ Brown rice
+ Broccoli
+ Onion
+ Cheddar cheese
+ Eggs
+ Fresh parsley
+ Evaporated milk
+ Worcestershire sauce
+ Spices to taste

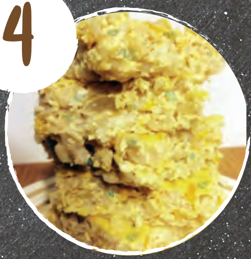

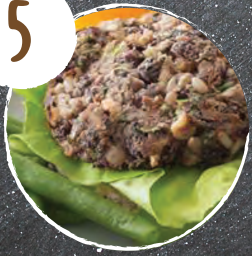

5 Black Bean Burgers

+ Brown rice
+ Black beans
+ Egg
+ Scallions
+ Cilantro
+ Oregano or basil
+ Garlic
+ Vegetable oil
+ Whole wheat buns
+ Spices to taste

For more information about these recipes go to: www.whatscooking.fns.usda.gov

ChooseMyPlate.gov

USDA
United States Department of Agriculture

10 tips
Nutrition
Education Series

MyPlate MyWins

Based on the
Dietary
Guidelines
for Americans

Got your dairy today?

The Dairy Group includes milk, yogurt, cheese, and fortified soymilk. They provide calcium, vitamin D, potassium, protein, and other nutrients needed for good health throughout life. Choices should be low-fat or fat-free—to cut calories and saturated fat. How much is needed? Older children, teens, and adults need 3 cups* a day, while children 4 to 8 years old need 2½ cups, and children 2 to 3 years old need 2 cups.

1 "Skim" the fat

Drink fat-free (skim) or low-fat (1%) milk. If you currently drink whole milk, gradually switch to lower fat versions. This change cuts saturated fat and calories but doesn't reduce calcium or other essential nutrients.

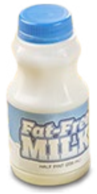

2 Boost potassium and vitamin D, and cut sodium

Choose fat-free or low-fat milk or yogurt more often than cheese. Milk and yogurt have more potassium and less sodium than most cheeses. Also, almost all milk and many yogurts are fortified with vitamin D.

3 Top off your meals

Use fat-free or low-fat milk on cereal and oatmeal. Top fruit salads and baked potatoes with low-fat yogurt instead of higher fat toppings such as sour cream.

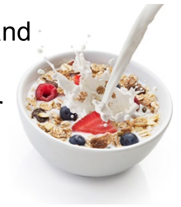

4 Choose cheeses with less fat

Many cheeses are high in saturated fat. Look for "reduced-fat" or "low-fat" on the label. Try different brands or types to find the one that you like.

5 What about cream cheese?

Cream cheese, cream, and butter are not part of the dairy food group. They are high in saturated fat and have little or no calcium.

* What counts as a cup in the Dairy Group? 1 cup of milk, yogurt, or soy beverage; 1½ ounces of natural cheese; or 2 ounces of processed cheese.

6 Switch ingredients

When recipes such as dips call for sour cream, substitute plain yogurt. Use fat-free evaporated milk instead of cream, and try low-fat or fat-free ricotta cheese as a substitute for cream cheese.

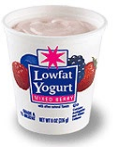

7 Limit added sugars

Flavored milks and yogurts, frozen yogurt, and puddings can contain a lot of added sugars. Get your nutrients from dairy foods with fewer or no added sugars.

8 Caffeinating?

If so, get your calcium along with your morning caffeine boost. Make or order coffee, a latte, or cappuccino with fat-free or low-fat milk.

9 Can't drink milk?

If you are lactose intolerant, try yogurt, lactose-free milk, or soymilk (soy beverage) to get your calcium. Calcium in some leafy greens is well absorbed, but eating several cups each day to meet calcium needs may be unrealistic.

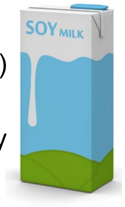

10 Take care of yourself and your family

Parents who drink milk and eat dairy foods show their kids that it is important for their health. Dairy foods are important to build the growing bones of kids and teens and to maintain bone health in adulthood.

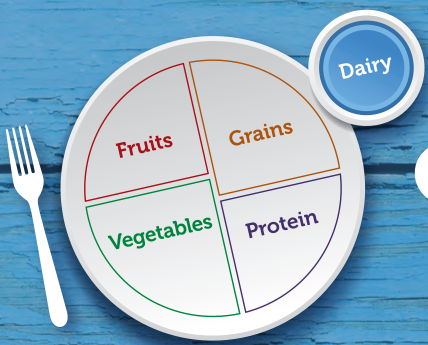

Yogurt 5 WAYS

MOVE TO LOW-FAT OR FAT-FREE DAIRY!

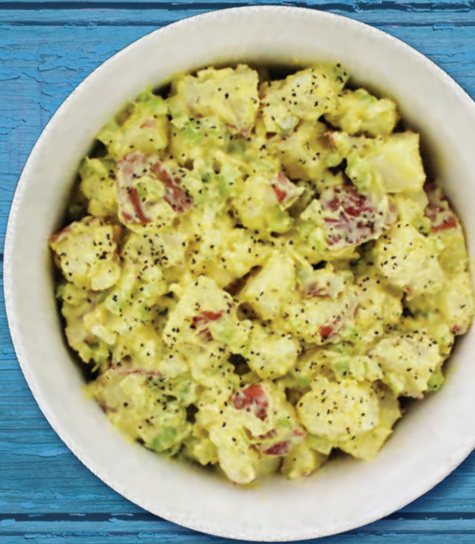

Red Potato Salad

+ Plain yogurt (fat-free)
+ Mayonnaise (low-fat)
+ Yellow mustard
+ Red potatoes
+ Celery
+ Onion
+ Salt & pepper

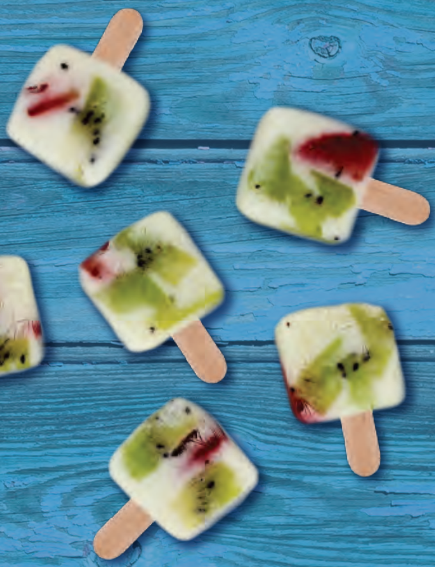

Strawberry Kiwi Pops

+ Vanilla yogurt (low-fat)
+ Kiwi
+ Strawberries
+ Ice cube tray or paper cups

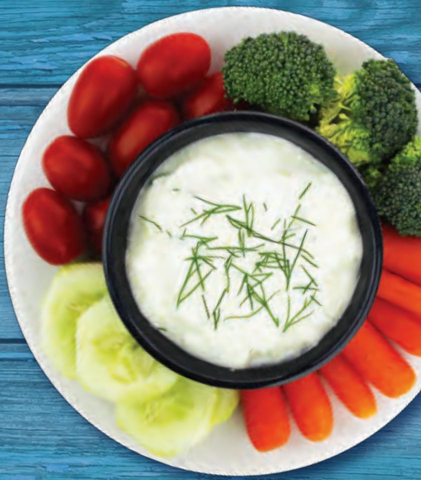

Cucumber Yogurt Dip

+ Plain yogurt (low-fat)
+ Cucumbers
+ Sour cream (low-fat)
+ Lemon juice
+ Dill
+ Garlic clove
+ Cherry tomatoes
+ Broccoli florets
+ Baby carrots

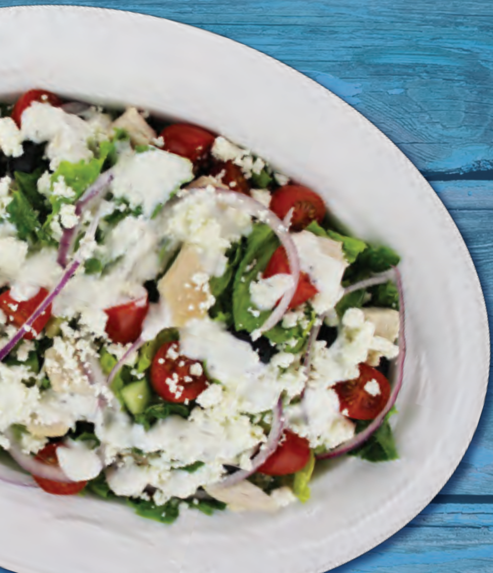

Greek Salad with Chicken

+ Romaine lettuce
+ Cherry tomatoes
+ Cucumber
+ Red onion
+ Black olives
+ Cooked chicken breast
+ Feta cheese
+ Plain Greek yogurt (fat-free)
+ Lemon juice
+ Olive oil
+ Garlic clove
+ Oregano
+ Salt & pepper

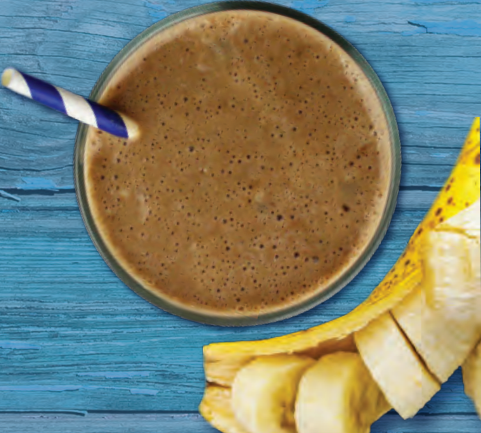

Peanut Butter Banana Smoothie

+ Bananas (frozen, ripe)
+ Milk (low-fat)
+ Vanilla yogurt (low-fat)
+ Peanut butter
+ Unsweetened cocoa powder
+ Ice

Find these yogurt recipes here: **https://go.usa.gov/xN5tQ**
For more recipes go to: **www.whatscooking.fns.usda.gov**

What's Cooking? USDA MIXING BOWL

FOUR WAYS TO GET GOOD FATS

Replace saturated fats with unsaturated fats as part of a healthy eating pattern. Unsaturated fats can help lower bad cholesterol and triglyceride levels, and they provide essential nutrients your body needs. Here are four easy and delicious ways to get more of the good fats.

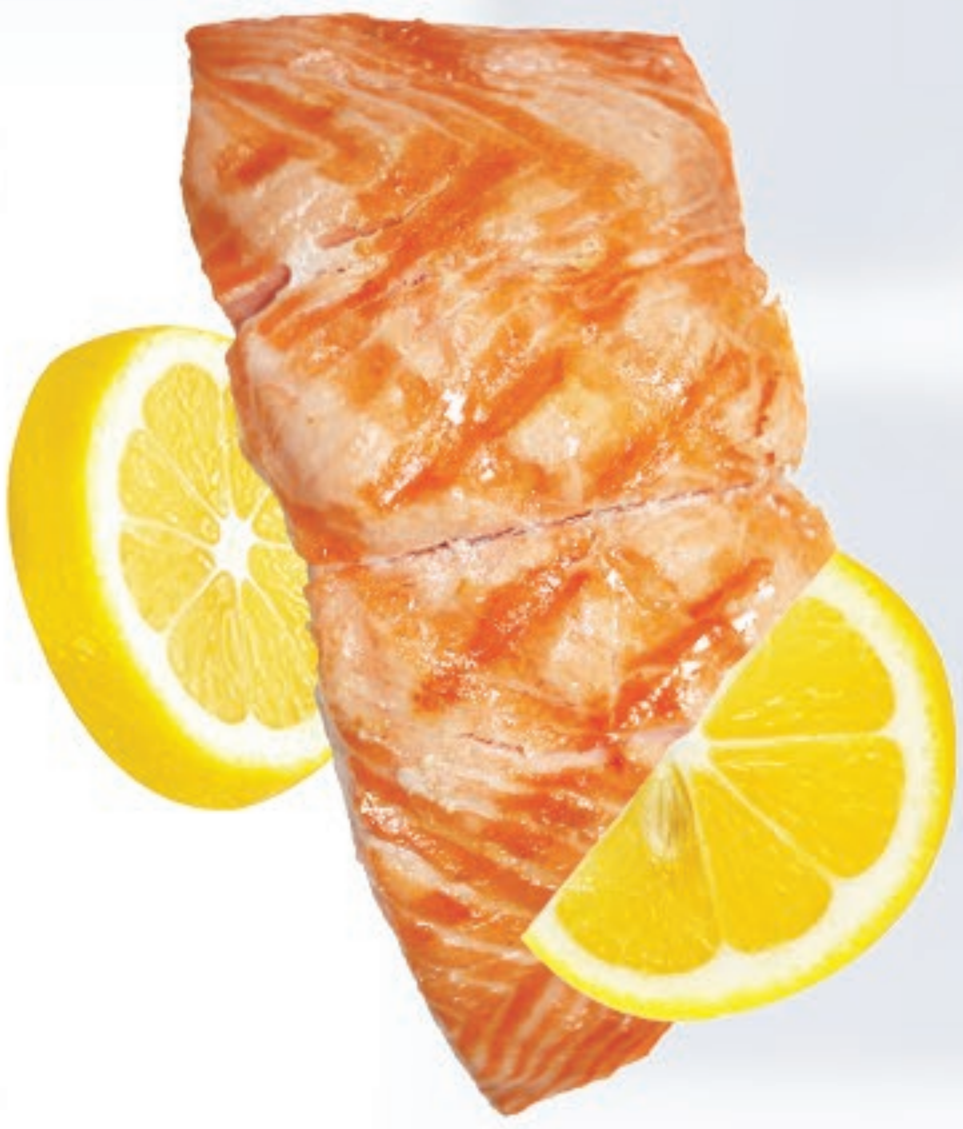

GO FISH

Eat at least 8 ounces of non-fried fish each week, which may be divided over two 3.5- to 4-ounce servings. Choose fatty or oily fish like albacore tuna, herring, lake trout, mackerel, sardines and salmon to get essential omega-3 fatty acids.

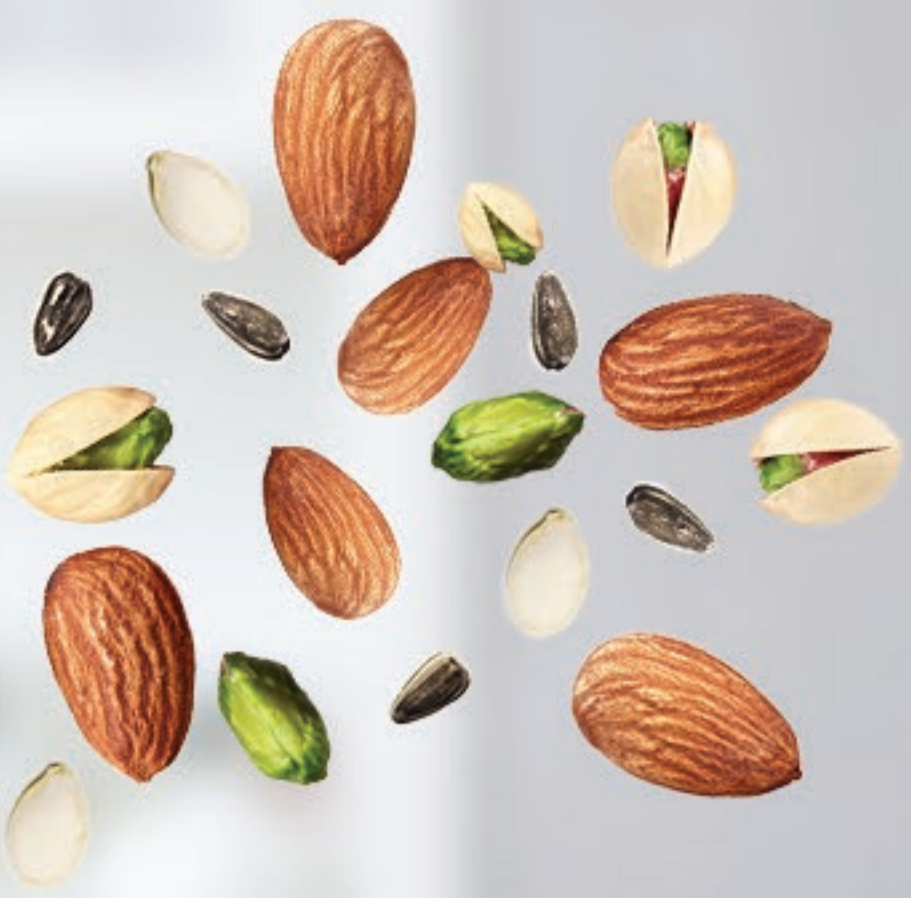

BE NUTTY

Munch on a small handful (about 1 oz.) of unsalted nuts and seeds for good fats, energy, protein and fiber. Good choices include almonds, hazelnuts, peanuts, pistachios, pumpkin seeds, sunflower seeds and walnuts.

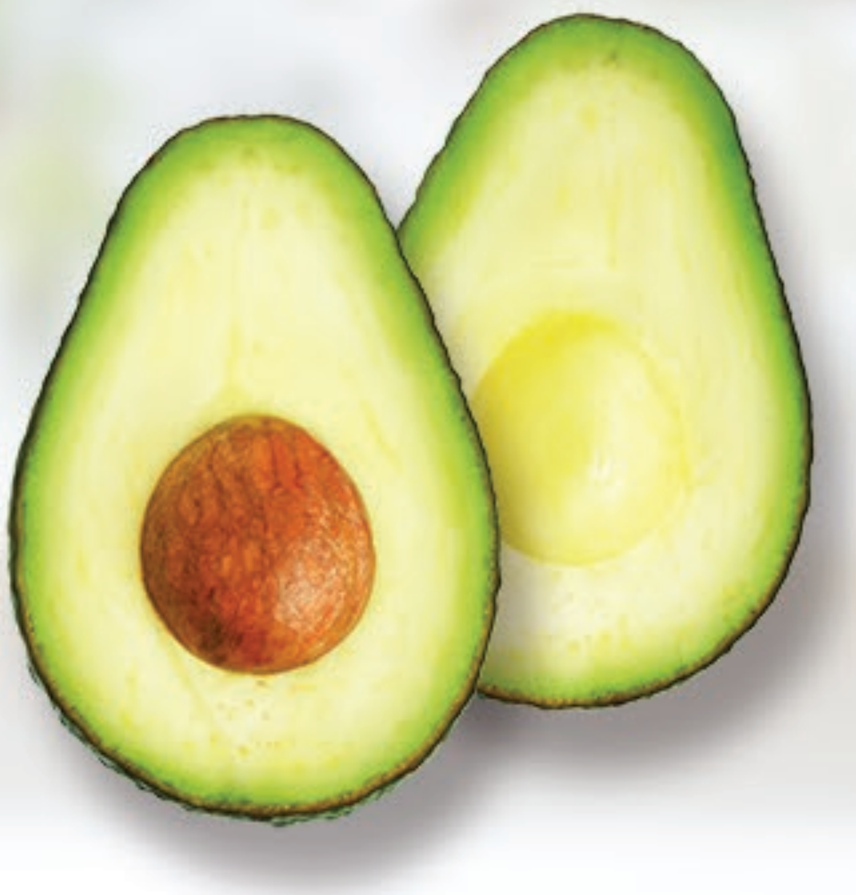

ADD AVOCADO

Snack, cook and bake with avocado to add healthy fats, fiber and essential vitamins and minerals.

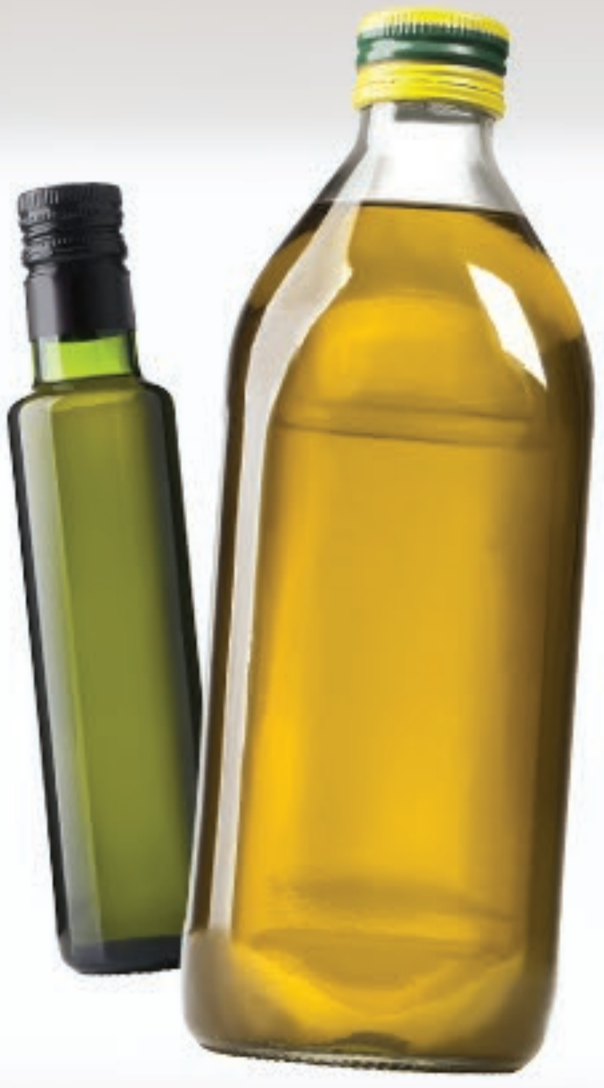

CHECK THE OILS

Use cooking and dressing oils that are lower in saturated fat. Good choices include avocado, canola, corn, grapeseed, olive, peanut, safflower, sesame, soybean and sunflower oils.

LEARN MORE AT
HEART.ORG/HEALTHYFORGOOD

©American Heart Association 2018

American Heart Association® life is why™ | Healthy For Good™

THE FACTS ON FAT

The American Heart Association recommends replacing bad (saturated) fats with good (unsaturated) fats as part of a healthy eating pattern.

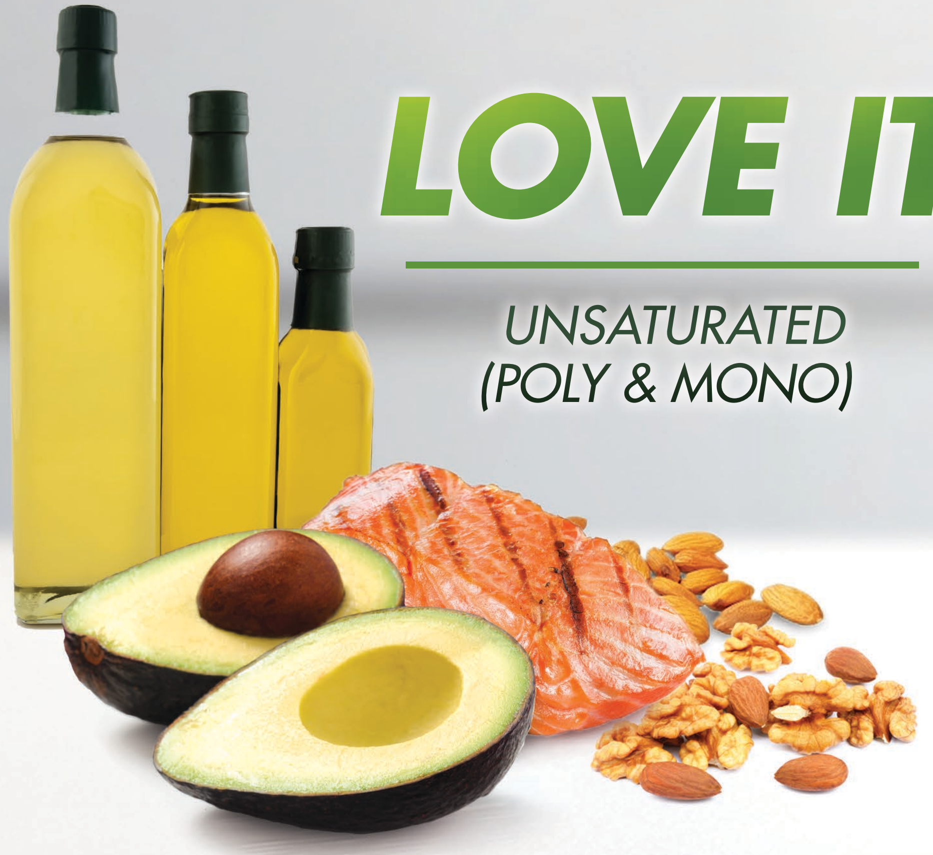

LOVE IT

UNSATURATED (POLY & MONO)

- Lowers rates of cardiovascular and all-cause mortality
- Lowers bad cholesterol & triglyceride levels
- Provides essential fats your body needs but can't produce itself

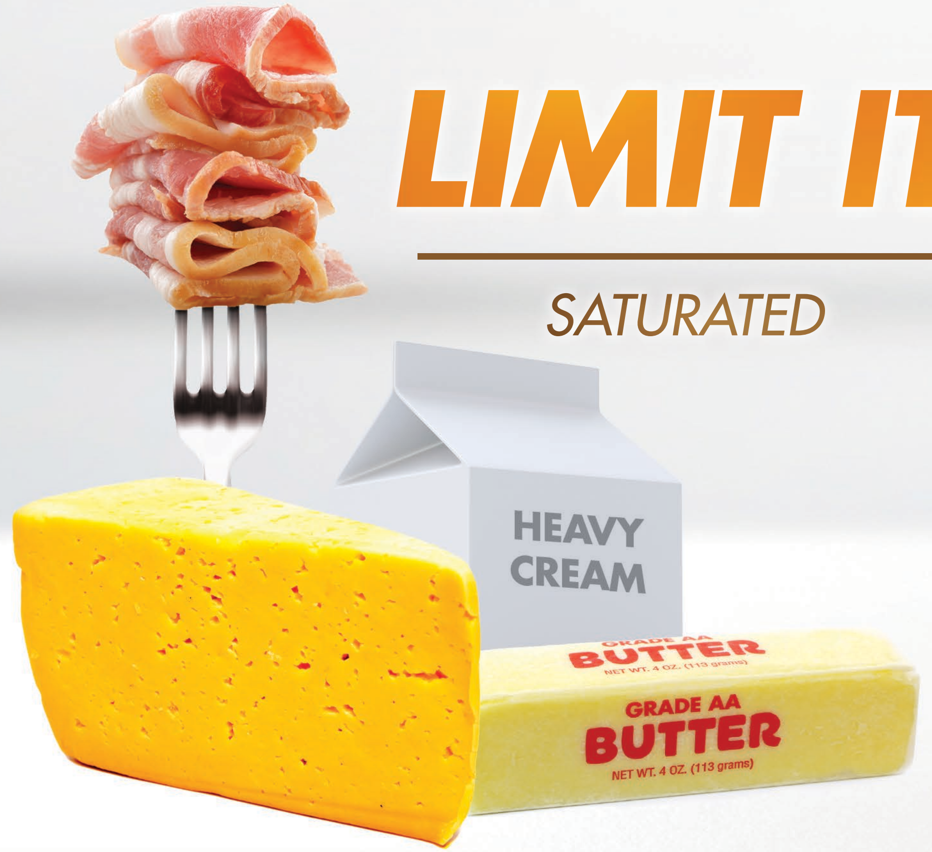

LIMIT IT

SATURATED

- Increases risk of cardiovascular disease
- Raises bad cholesterol levels

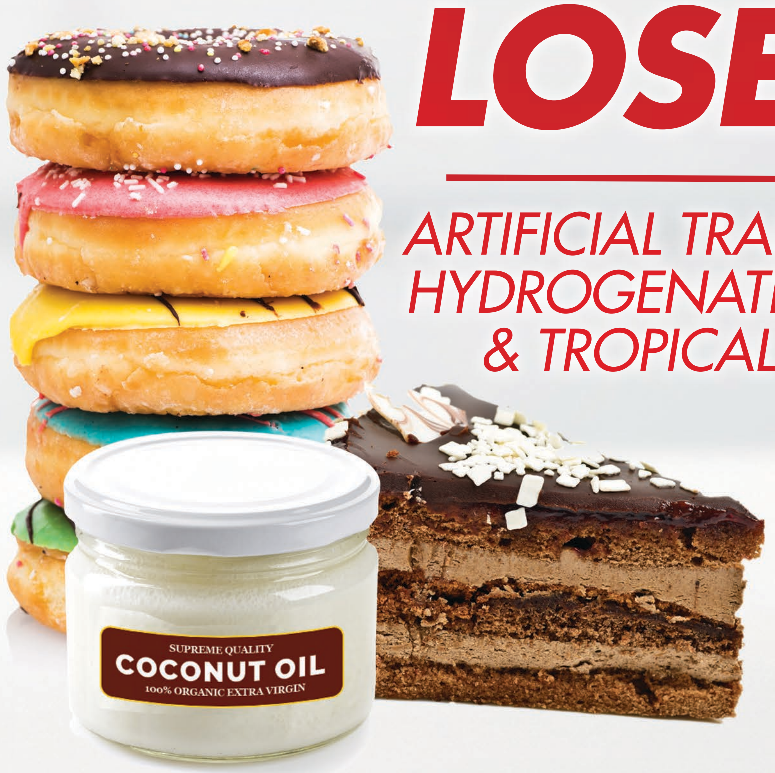

LOSE IT

ARTIFICIAL TRANS FAT, HYDROGENATED OILS & TROPICAL OILS

- Increases risk of heart disease
- Raises bad cholesterol levels

USDA
United States Department of Agriculture

10 tips
Nutrition
Education Series

MyPlate MyWins

Based on the
Dietary
Guidelines
for Americans

Eating better on a budget

Get the most for your budget! There are many ways to save money on the foods that you eat. The three main steps are planning before you shop, purchasing the items at the best price, and preparing meals that stretch your food dollars.

1 Plan, plan, plan!

Before you head to the grocery store, plan your meals for the week. Include meals like stews, casseroles, or soups, which "stretch" expensive items into more portions. Check to see what foods you already have and make a list for what you need to buy.

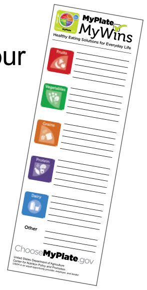

2 Get the best price

Check the local newspaper, online, and at the store for sales and coupons. Ask about a loyalty card for extra savings at stores where you shop. Look for specials or sales on meat and seafood—often the most expensive items on your list.

3 Compare and contrast

Locate the "Unit Price" on the shelf directly below the product. Use it to compare different brands and different sizes of the same brand to determine which is the best buy.

4 Buy in bulk

It is almost always cheaper to buy foods in bulk. Smart choices are large containers of low-fat yogurt and large bags of frozen vegetables. Before you shop, remember to check if you have enough freezer space.

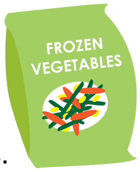

5 Buy in season

Buying fruits and vegetables in season can lower the cost and add to the freshness! If you are not going to use them all right away, buy some that still need time to ripen.

6 Convenience costs... go back to the basics

Convenience foods like frozen dinners, pre-cut fruits and vegetables, and take-out meals can often cost more than if you were to make them at home. Take the time to prepare your own—and save!

7 Easy on your wallet

Certain foods are typically low-cost options all year round. Try beans for a less expensive protein food. For vegetables, buy cabbage, sweet potatoes, or low-sodium canned tomatoes. As for fruits, apples and bananas are good choices.

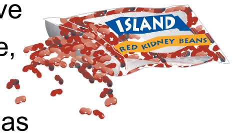

8 Cook once...eat all week!

Prepare a large batch of favorite recipes on your day off (double or triple the recipe). Freeze in individual containers. Use them throughout the week and you won't have to spend money on take-out meals.

9 Get creative with leftovers

Spice up your leftovers—use them in new ways. For example, try leftover chicken in a stir-fry, over a garden salad, or in chili. Remember, throwing away food is throwing away your money!

10 Eating out

Restaurants can be expensive. Save money by getting the early bird special, going out for lunch instead of dinner, or looking for "2 for 1" deals. Ask for water instead of ordering other beverages, which add to the bill.

Center for Nutrition Policy and Promotion
USDA is an equal opportunity provider, employer, and lender.

Go to ChooseMyPlate.gov
for more information.

DG TipSheet No. 16
November 2011
Revised October 2016

10 tips
Nutrition Education Series

be choosy in the dining hall

10 tips for healthy eating in the dining hall

Dining halls are full of healthy food options. You just need to know which foods to put on your tray. Use these tips to plan your food choices and know which options are best for you.

1 know what you're eating
Many dining halls post menus with nutrition information. Look at the menus ahead of time, so you can be ready to create healthy, balanced meals when you get there. Having a plan is the first step in making smarter eating decisions! Visit ChooseMyPlate.gov to find information and tools like SuperTracker to help you make meal selection a breeze.

2 enjoy your food, but eat less
Everybody loves the all-you-can-eat dining hall! To resist the urge of eating too much, take smaller portions and use a smaller plate. Remember you can always go back if you are still hungry.

3 make half your grains whole grains!
Whether you're at the sandwich station or pouring yourself a bowl of cereal in the morning, make the switch to whole grains like 100% whole-grain bread and oatmeal.

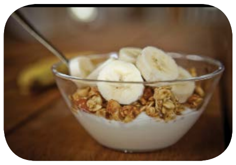

4 re-think your drink
Americans drink about 400 calories every day. Consider how often you drink sugary beverages such as sodas, cappuccinos, energy drinks, fruit beverages, sweetened teas, and sports drinks. Drinking water instead of sugary beverages can help you manage your calories.

5 make half your plate fruits and veggies
Fruits and veggies can make your meals more nutritious, colorful, and flavorful. Add to pastas, eggs, pizza, sandwiches, and soups. Try spinach in a wrap or add pineapple to your pizza.

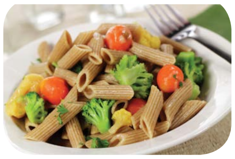

6 make it your own!
Don't feel like you have to choose pre-made plates. Design your own meal! Fresh veggies from the salad bar can be thrown into your omelet for brunch, or grab some tofu on your way to the pasta station for lean protein.

7 slow down on the sauces
Sauces, gravies, and dressings tend to be high in fat and sodium. Watch out for foods prepared with a lot of oil, butter, or topped with heavy condiments, such as mayonnaise. You don't have to do away with sauces and condiments all together; just ask for less or put them on the side. Reducing extras will help you manage your weight.

8 be on your guard at the salad bar
Most veggies get the green light but limit foods high in fat and sodium such as olives, bacon bits, fried noodles, croutons, and pasta or potato salads that are made with mayo and oil. Stick to fat-free or low-fat dressings on the side.

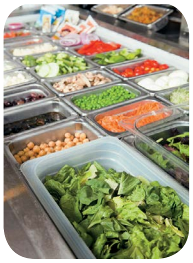

9 make dessert special
Save dessert for a Friday night treat or on special occasions. When you can't resist, opt for something healthy, such as a fruit and yogurt parfait.

10 don't linger
Dining halls should be just that, where you eat. Although it's great to chat with friends while you eat, avoid staying for long periods of time to reduce your temptation to keep eating.

United States Department of Agriculture

Go to www.ChooseMyPlate.gov for more information.

DG TipSheet No. 26
April 2013
Center for Nutrition Policy and Promotion
USDA is an equal opportunity provider and employer.

10 tips
Nutrition Education Series

MyPlate MyWins

Based on the Dietary Guidelines for Americans

Eating foods away from home

Full-service and fast-food restaurants, convenience stores, and grocery stores offer a variety of meal options. Typically, these meals are higher in calories, saturated fat, sodium, and added sugars than the food you prepare at home. Think about ways to make healthier choices when eating food away from home.

1 Consider your drink

Choose water, unsweetened tea, and other drinks without added sugars to complement your meal. If you drink alcohol, choose drinks lower in added sugars and be aware of the alcohol content of your beverage. Keep in mind that many coffee drinks may be high in saturated fat and added sugar.

2 Savor a salad

Start your meal with a salad packed with vegetables to help you feel satisfied sooner. Ask for dressing on the side and use a small amount of it.

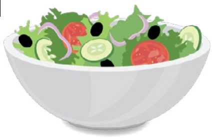

3 Share a dish

Share a dish with a friend or family member. Or, ask the server to pack up half of your entree before it comes to the table to control the amount you eat.

4 Customize your meal

Order a side dish or an appetizer-sized portion instead of a regular entree. They're usually served on smaller plates and in smaller amounts.

5 Pack your snack

Pack fruit, sliced vegetables, low-fat string cheese, or unsalted nuts to eat during road trips or long commutes. No need to stop for other food when these snacks are ready-to-eat.

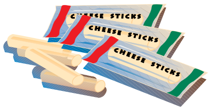

6 Fill your plate with vegetables and fruit

Stir-fries, kabobs, or vegetarian menu items usually have more vegetables. Select fruits as a side dish or dessert.

7 Compare the calories, fat, and sodium

Many menus now include nutrition information. Look for items that are lower in calories, saturated fat, and sodium. Check with your server if you don't see them on the menu. For more information, check www.FDA.gov.

8 Pass on the buffet

Have an item from the menu and avoid the "all-you-can-eat" buffet. Steamed, grilled, or broiled dishes have fewer calories than foods that are fried in oil or cooked in butter.

9 Get your whole grains

Request 100% whole-wheat breads, rolls, and pasta when choosing sandwiches, burgers, or main dishes.

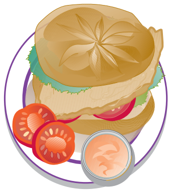

10 Quit the "clean your plate club"

You don't have to eat everything on your plate. Take leftovers home and refrigerate within 2 hours. Leftovers in the refrigerator are safe to eat for about 3 to 4 days.

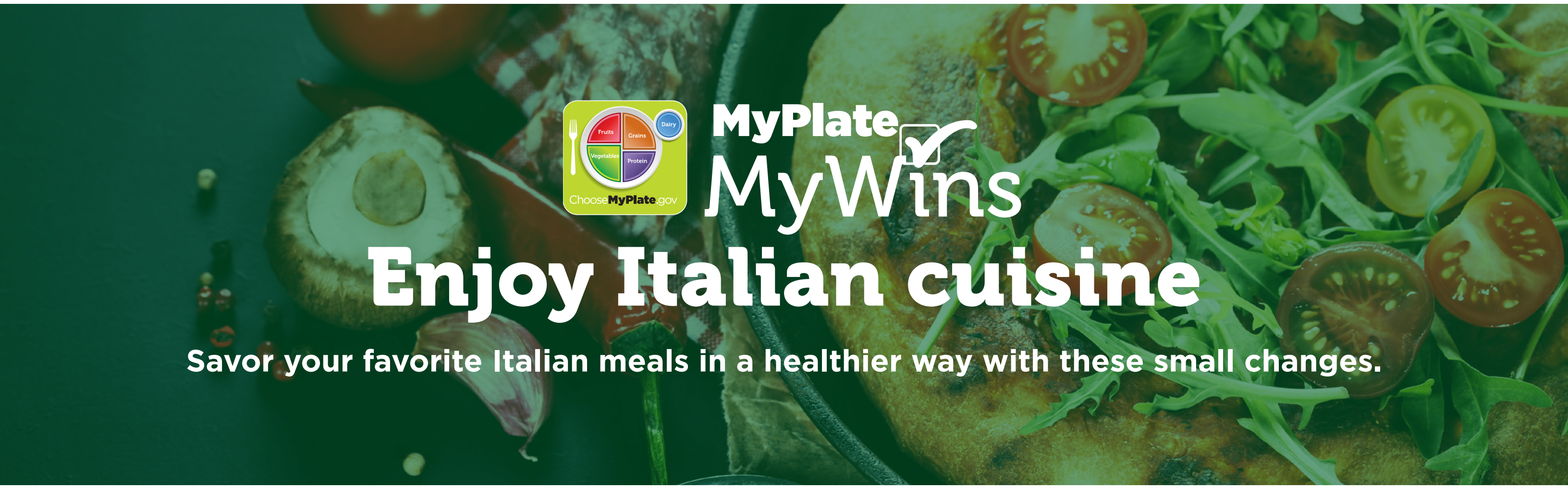

MyPlate MyWins

Enjoy Italian cuisine

Savor your favorite Italian meals in a healthier way with these small changes.

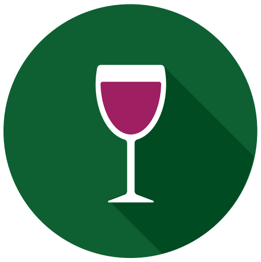

Balance your options

You don't necessarily have to skip the bread, wine, and dessert. Consider choosing just one of these options and pass on refills.

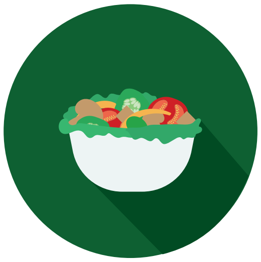

Start with salad

Choose a mixed green salad with vinaigrette instead of fried appetizers, which are higher in sodium and saturated fat.

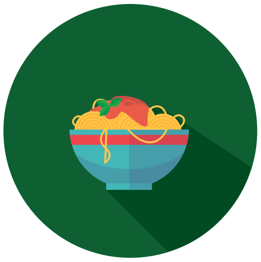

Go for whole grains

Ask for whole-wheat pasta or pizza crust. Whole grains have more fiber and nutrients than refined grains.

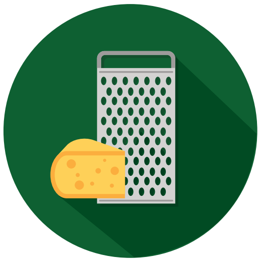

Lighten up on cheese

Ask for a lighter sprinkling of cheese on pizza or pasta to cut back on the saturated fat and sodium.

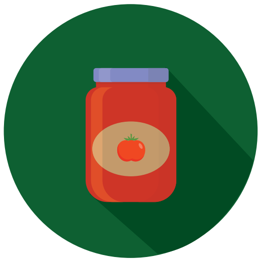

Choose red sauces

Pick sauces made from vegetables, like marinara sauce, rather than heavy cream or butter sauce.

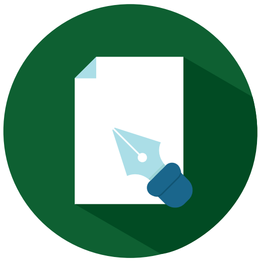

List more tips

____

____

____

____

MPMW Tipsheet No. 6
December 2016
Center for Nutrition Policy and Promotion

USDA is an equal opportunity provider, employer, and lender.

USDA
United States Department of Agriculture

MyPlate MyWins

Redo your coffee shop stop

Whether going to your local coffee shop is a daily ritual or a special weekend outing, consider these ways you can move toward better choices.

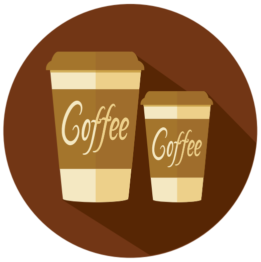

Downsize your drink

If a large coffee drink is your go-to, consider whether a smaller size would hit the spot.

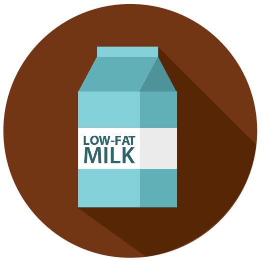

Do dairy right

For lattes and cappuccinos, shift from whole milk to low-fat or fat-free (skim) milk to reduce the amount of saturated fat.

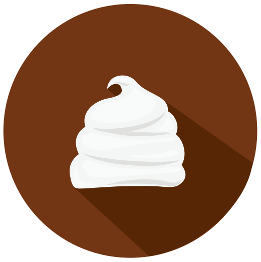

Skip the "whip"

Leave off the extras like whipped cream and caramel drizzle. The calories from added sugars in the toppings alone can really add up.

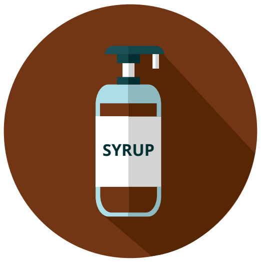

Cut the syrup in half

Ask for fewer pumps of sweetener in your drink. A sprinkle of cinnamon or cocoa powder can add flavor without added sugars.

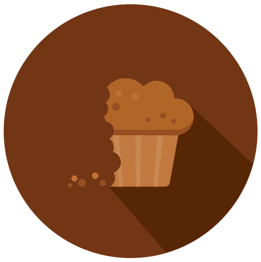

Split the sweets

Share a muffin or pastry with a friend. It can be high in calories from added sugars and saturated fat.

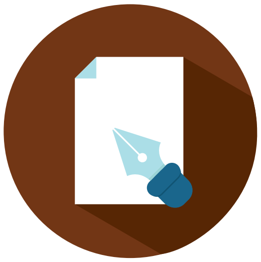

List more tips

____

____

____

____

MPMW Tipsheet No. 8
December 2016
Center for Nutrition Policy and Promotion

Based on the Dietary Guidelines for Americans
Go to ChooseMyPlate.gov for more information.

USDA is an equal opportunity provider, employer, and lender.

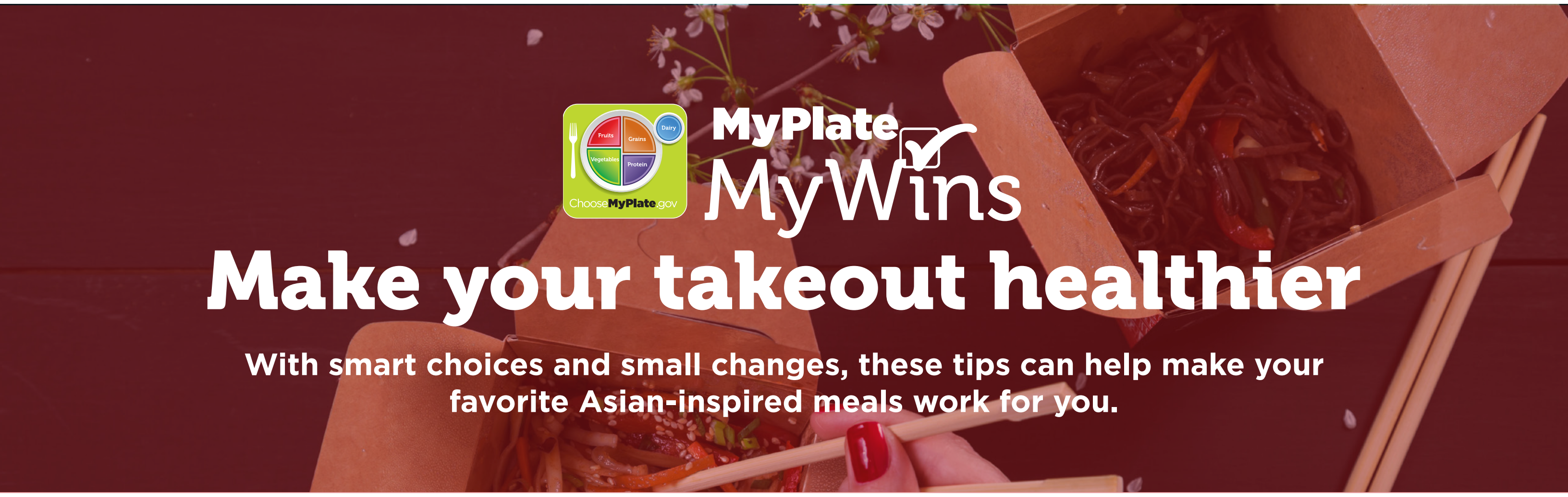

MyPlate MyWins

Make your takeout healthier

With smart choices and small changes, these tips can help make your favorite Asian-inspired meals work for you.

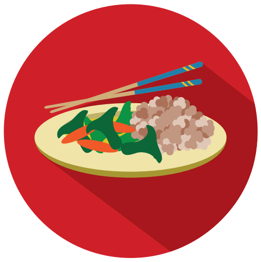

Look for veggies

Pick dishes that highlight veggies, like chicken and broccoli or a vegetable stir-fry. Be mindful of the type and amount of sauce used.

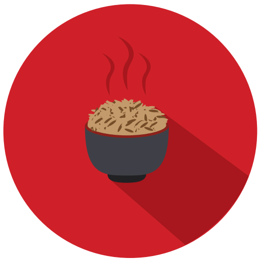

Try steamed foods

Many foods can be steamed rather than fried. Steamed dumplings and rice are lower in saturated fat than the fried versions.

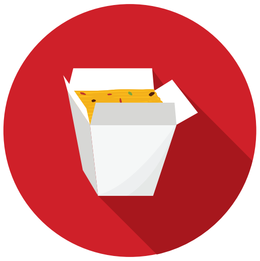

Adjust your order

Most restaurants are happy to accommodate your requests. Ask that your food be cooked with less oil or half the sauce.

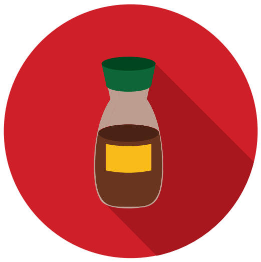

Add sauces sparingly

Sodium in soy sauce and calories from added sugars in duck and teriyaki sauces can add up quickly, so be mindful of how much you use.

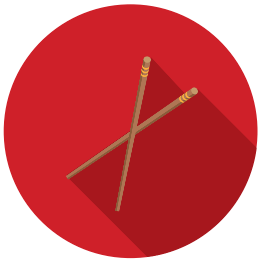

Use chopsticks

Unless you're an expert, eating with chopsticks can help you slow down and recognize when you're full so you don't overeat.

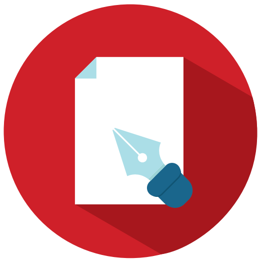

List more tips

____

____

____

____

MPMW Tipsheet No. 9
December 2016
Center for Nutrition Policy and Promotion

Based on the Dietary Guidelines for Americans
Go to ChooseMyPlate.gov for more information.

USDA is an equal opportunity provider, employer, and lender.

Vary your protein routine

Protein foods include both animal (meat, poultry, seafood, and eggs) and plant (beans, peas, soy products, nuts, and seeds) sources. We all need protein—but most Americans eat enough, and some eat more than they need. How much is enough? Most people, ages 9 and older, should eat 5 to 7 ounces* of protein foods each day depending on overall calorie needs.

1 Vary your protein food choices

Eat a variety of foods from the Protein Foods Group each week. Experiment with beans or peas, nuts, soy, and seafood as main dishes.

2 Choose seafood twice a week

Eat seafood in place of meat or poultry twice a week. Select a variety of seafood, including those that are higher in oils and low in mercury, such as salmon, trout, and herring.

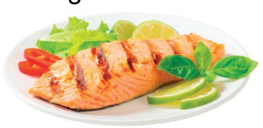

3 Select lean meat and poultry

Choose lean cuts of meat like round or sirloin and ground beef that is at least 93% lean. Trim or drain fat from meat and remove poultry skin.

4 Save with eggs

Eggs can be an inexpensive protein option and part of a healthy eating style. Make eggs part of your weekly choices.

5 Eat plant protein foods more often

Try beans and peas (kidney, pinto, black, or white beans; split peas; chickpeas; hummus), soy products (tofu, tempeh, veggie burgers), nuts, and seeds. They are lower in saturated fat and some are higher in fiber.

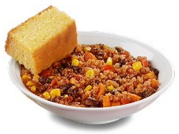

* What counts as an ounce of protein foods? 1 ounce lean meat, poultry, or seafood; 1 egg; ¼ cup cooked beans or peas; ½ ounce nuts or seeds; or 1 tablespoon peanut butter.

6 Consider nuts and seeds

Choose unsalted nuts or seeds as a snack, on salads, or in main dishes. Nuts and seeds are a concentrated source of calories, so eat small portions to keep calories in check.

7 Keep it tasty and healthy

Try grilling, broiling, roasting, or baking—they don't add extra fat. Some lean meats need slow, moist cooking to be tender—try a slow cooker for them. Avoid breading meat or poultry, which adds calories.

8 Make a healthy sandwich

Choose turkey, roast beef, canned tuna or salmon, or peanut butter for sandwiches.Many deli meats, such as regular bologna or salami, are high in fat and sodium—make them occasional treats only.

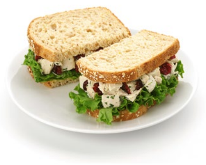

9 Think small when it comes to meat portions

Get the flavor you crave but in a smaller portion. Make or order a small turkey burger or a "petite" size steak.

10 Check the sodium

Check the Nutrition Facts label to limit sodium. Salt is added to many canned foods—including soups, vegetables, beans, and meats. Many processed meats—such as ham, sausage, and hot dogs—are high in sodium. Some fresh chicken, turkey, and pork are brined in a salt solution for flavor and tenderness.

10 tips
Nutrition Education Series

eat seafood twice a week

10 tips to help you eat more seafood

Twice a week, make seafood—fish and shellfish—the main protein food on your plate.* Seafood contains a range of nutrients, including healthy omega-3 fats. According to the *2010 Dietary Guidelines for Americans*, eating about 8 ounces per week (less for young children) of a variety of seafood can help prevent heart disease.

1 eat a variety of seafood

Include some that are higher in omega-3s and lower in mercury, such as salmon, trout, oysters, Atlantic and Pacific mackerel, herring, and sardines.

2 keep it lean and flavorful

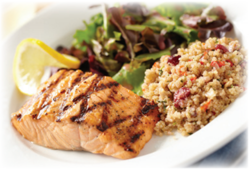

Try grilling, broiling, roasting, or baking—they don't add extra fat. Avoid breading or frying seafood and creamy sauces, which add calories and fat. Using spices or herbs, such as dill, chili powder, paprika, or cumin, and lemon or lime juice, can add flavor without adding salt.

3 shellfish counts too!

Oysters, mussels, clams, and calamari (squid) all supply healthy omega-3s. Try mussels marinara, oyster stew, steamed clams, or pasta with calamari.

4 keep seafood on hand

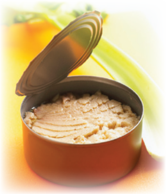

Canned seafood, such as canned salmon, tuna, or sardines, is quick and easy to use. Canned white tuna is higher in omega-3s, but canned "light" tuna is lower in mercury.

5 cook it safely

Check oysters, mussels, and clams before cooking. If shells don't clamp shut when you tap them, throw them away. After cooking, also toss any that didn't open. This means that they may not be safe to eat. Cook shrimp, lobster, and scallops until they are opaque (milky white). Cook fish to 145°F, until it flakes with a fork.

*This recommendation does not apply to vegetarians.

6 get creative with seafood

Think beyond the fish fillet. Try salmon patties, a shrimp stir-fry, grilled fish tacos, or clams with whole-wheat pasta. Add variety by trying a new fish such as grilled Atlantic or Pacific mackerel, herring on a salad, or oven-baked pollock.

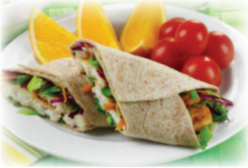

7 put it on a salad or in a sandwich

Top a salad with grilled scallops, shrimp, or crab in place of steak or chicken. Use canned tuna or salmon for sandwiches in place of deli meats, which are often higher in sodium.

8 shop smart

Eating more seafood does not have to be expensive. Whiting, tilapia, sardines, canned tuna, and some frozen seafood are usually lower cost options. Check the local newspaper, online, and at the store for sales, coupons, and specials to help save money on seafood.

9 grow up healthy with seafood

Omega-3 fats from seafood can help improve nervous system development in infants and children. Serve seafood to children twice a week in portions appropriate for their age and appetite. A variety of seafood lower in mercury should also be part of a healthy diet for women who are pregnant or breastfeeding.

10 know your seafood portions

To get 8 ounces of seafood a week, use these as guides: A drained can of tuna is about 3 to 4 ounces, a salmon steak ranges from 4 to 6 ounces, and 1 small trout is about 3 ounces.

USDA United States Department of Agriculture
Center for Nutrition Policy and Promotion

Go to www.ChooseMyPlate.gov for more information.

DG TipSheet No. 15
December 2011
USDA is an equal opportunity provider and employer.

American Heart Association. | Healthy For Good™

PROTEIN PORTION

Our bodies need protein to be healthy and strong, but a serving is probably smaller than you think. Here's what a healthy serving of some common protein foods looks like.

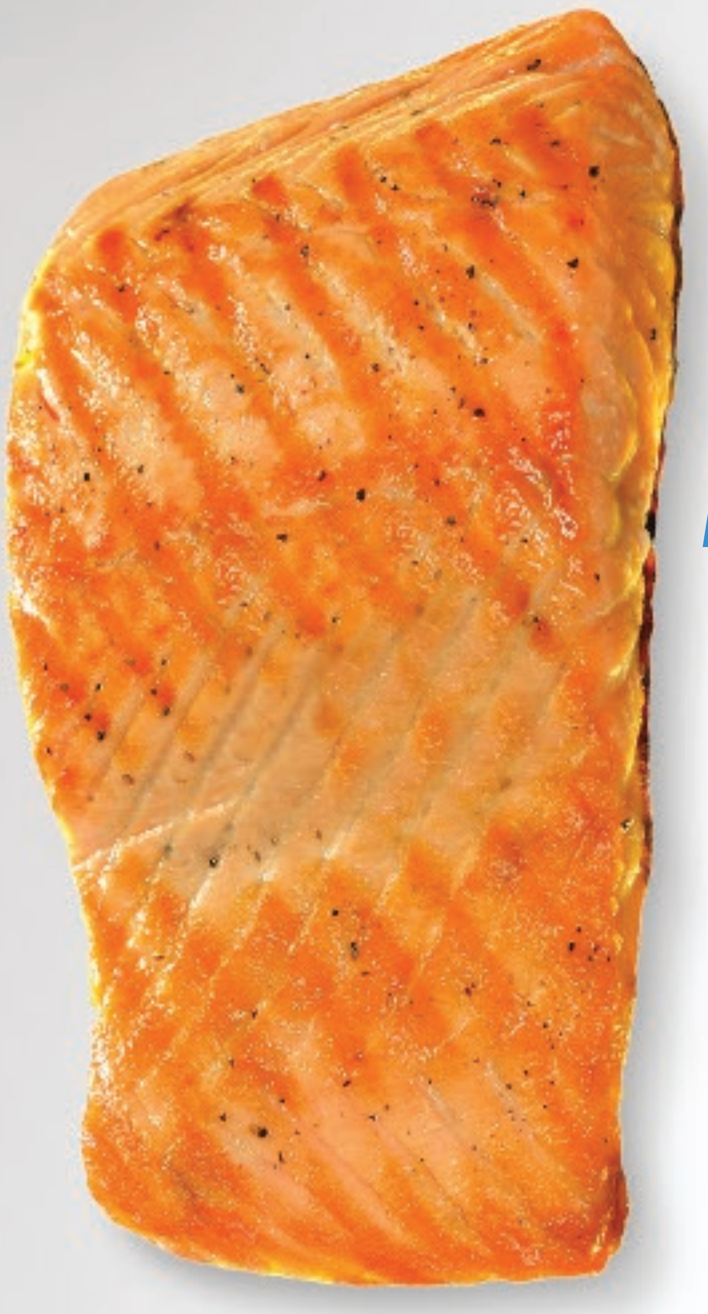

NON-FRIED FISH
3.5 ounces

LEAN BEEF
3 ounces

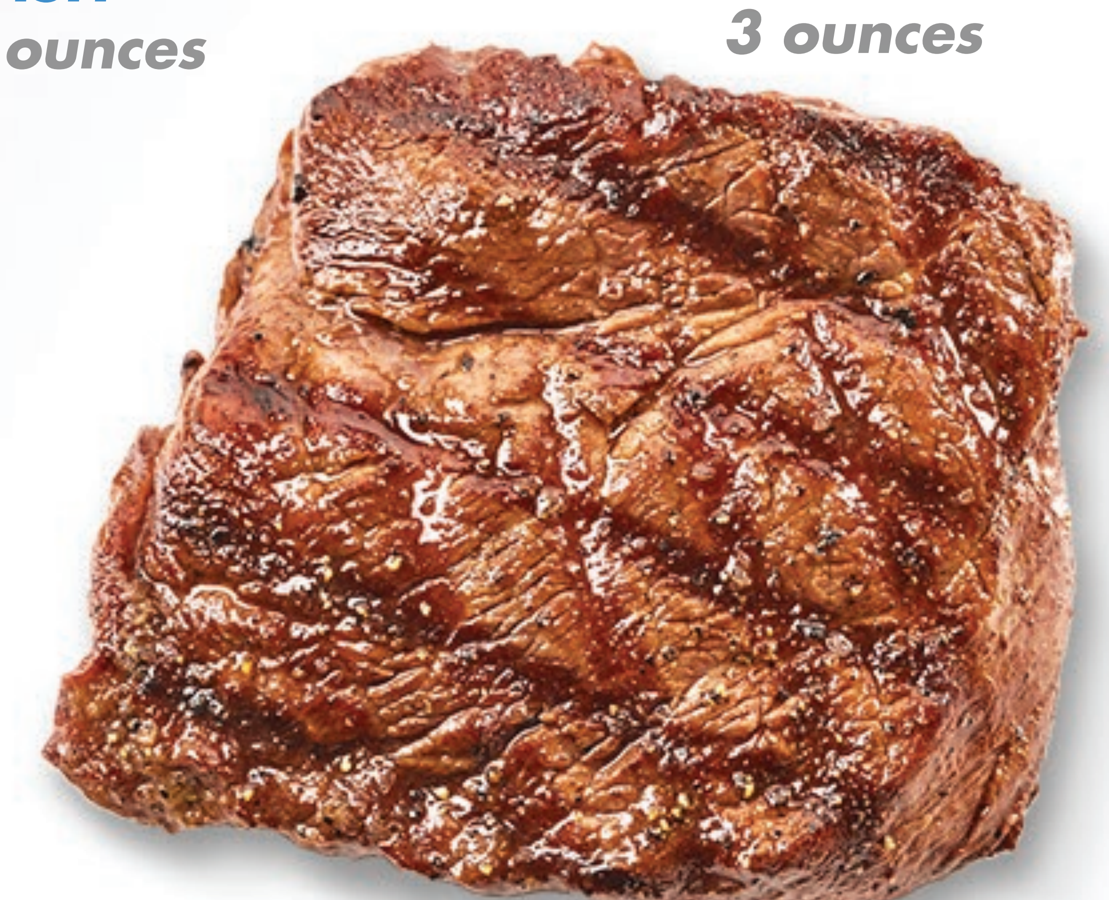

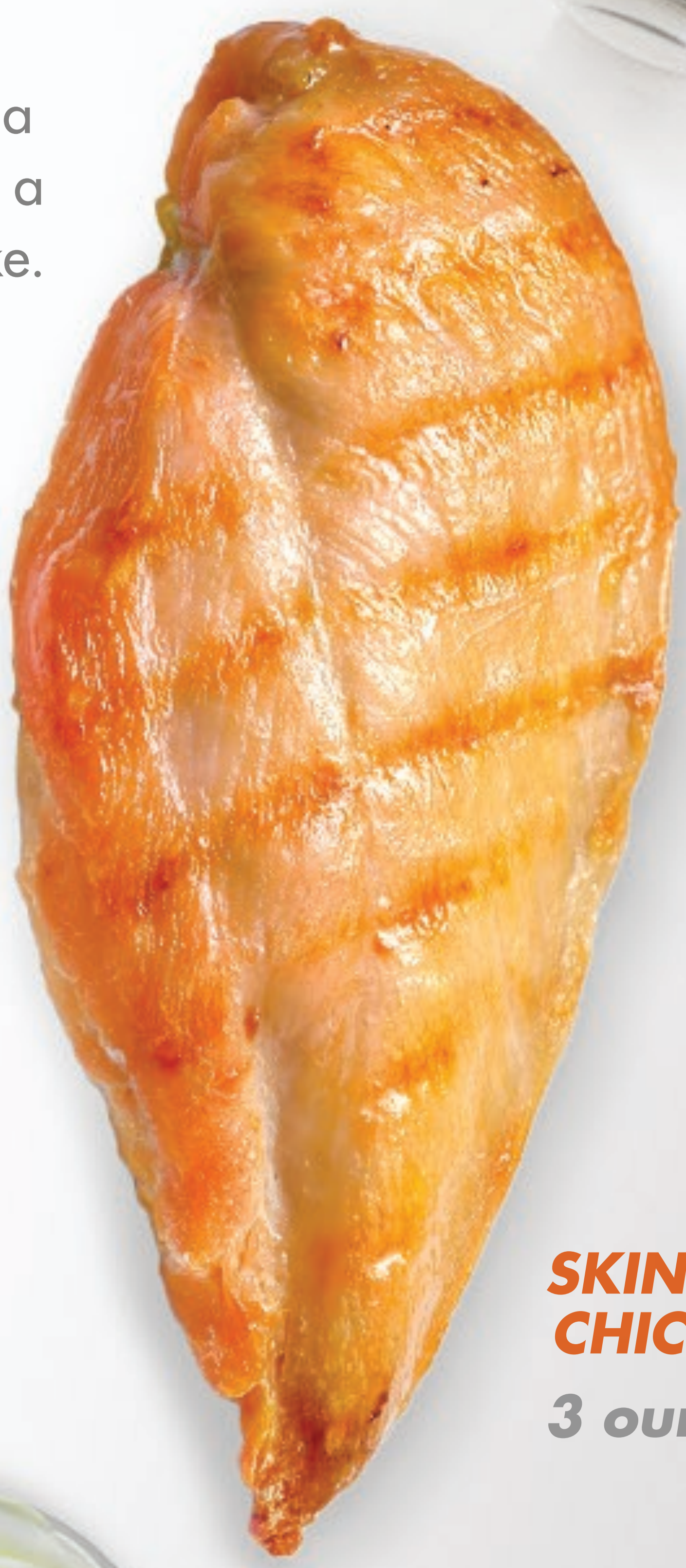

SKINLESS CHICKEN
3 ounces

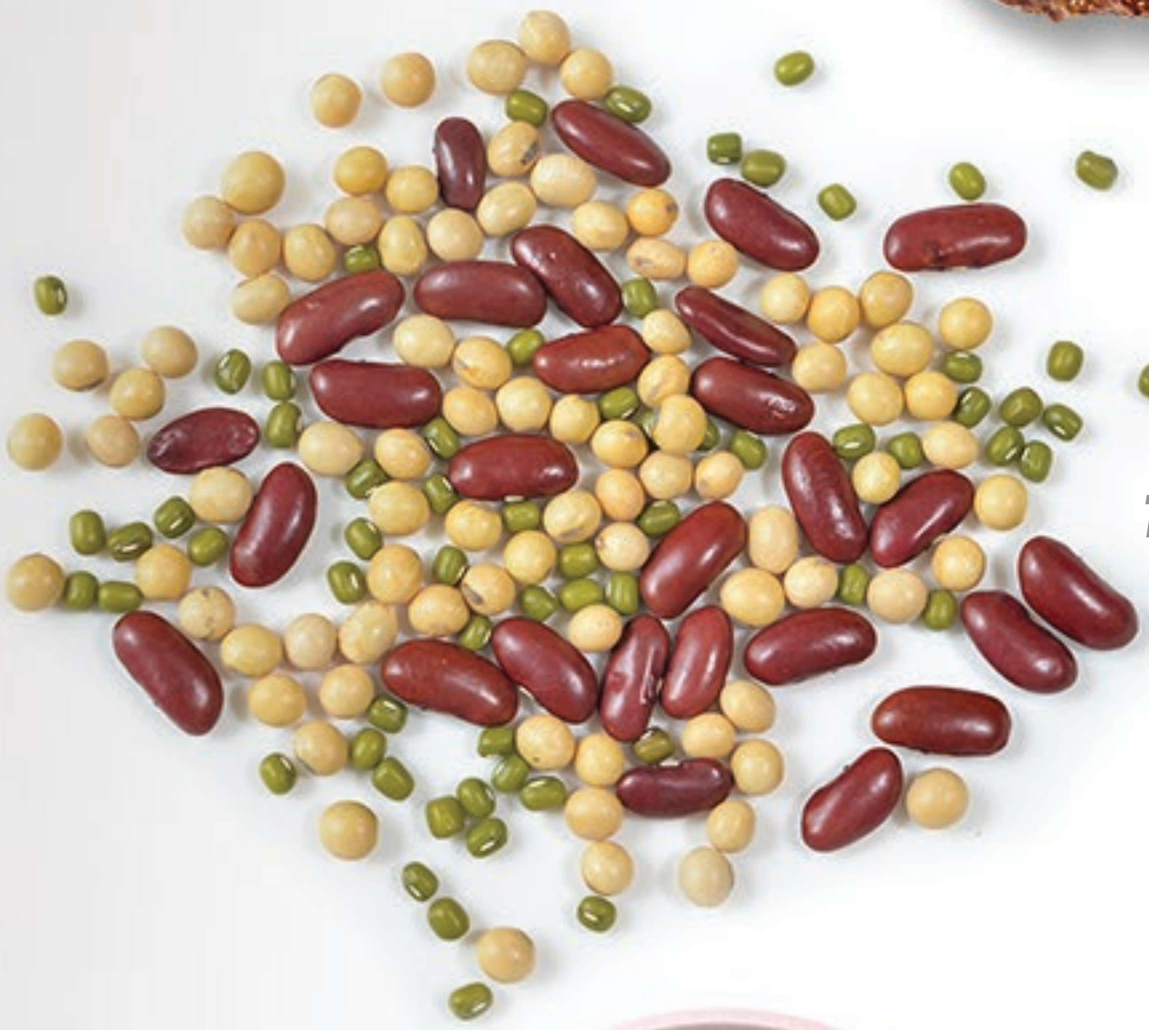

BEANS & LEGUMES
1/2 cup cooked

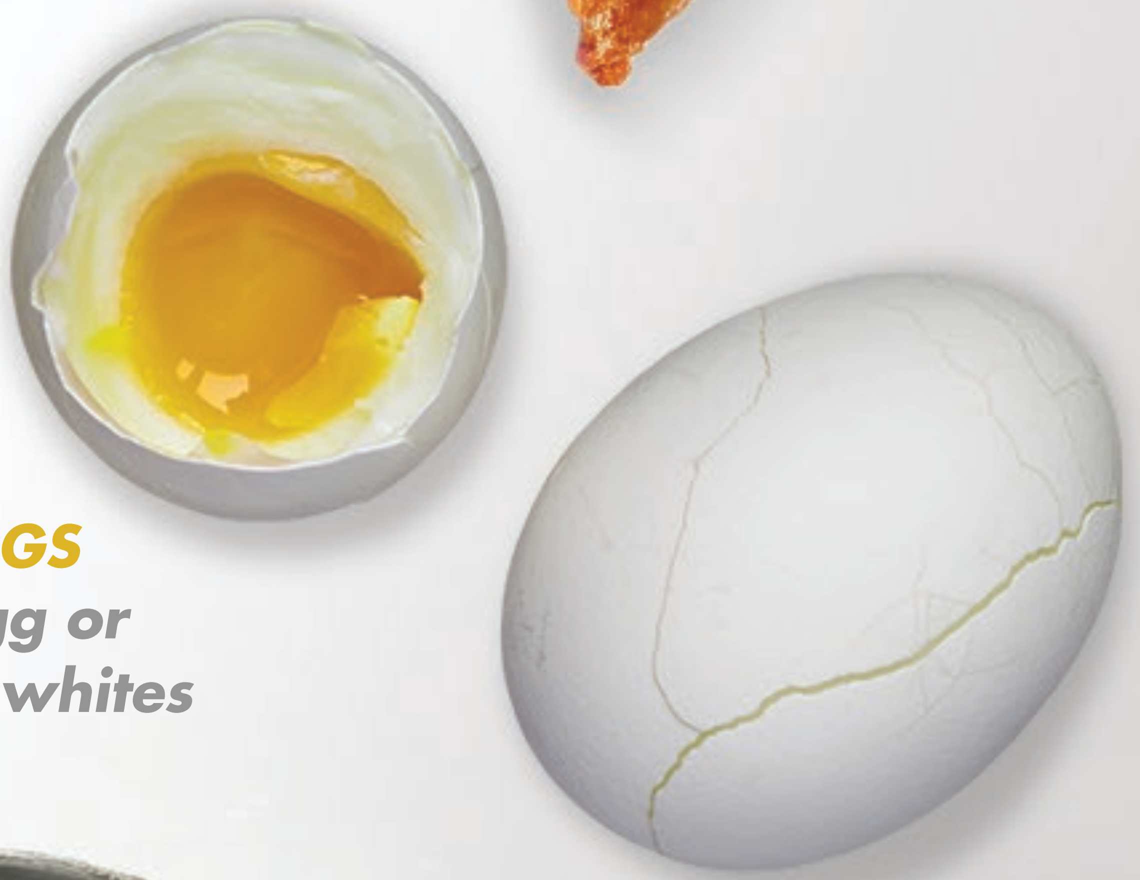

EGGS
1 egg or
2 egg whites

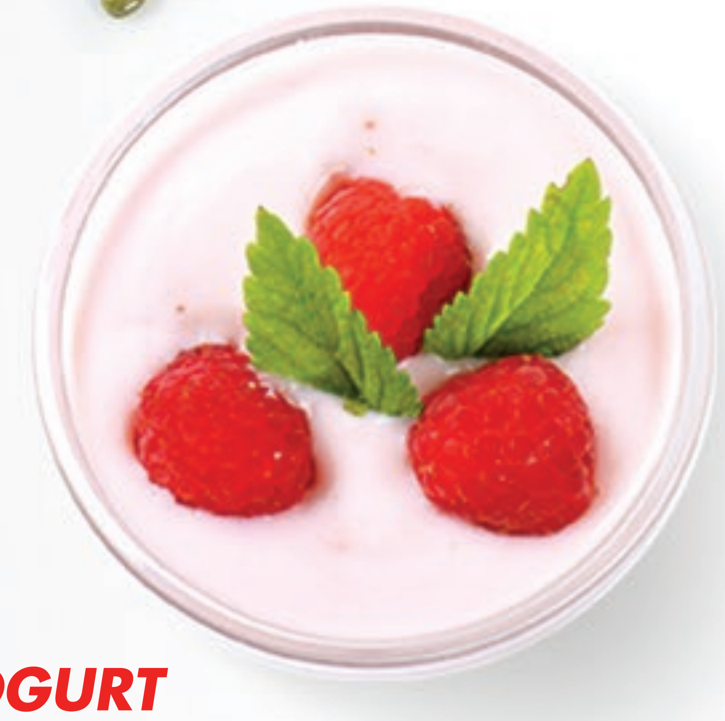

YOGURT
(low-fat or fat-free)
6 ounces

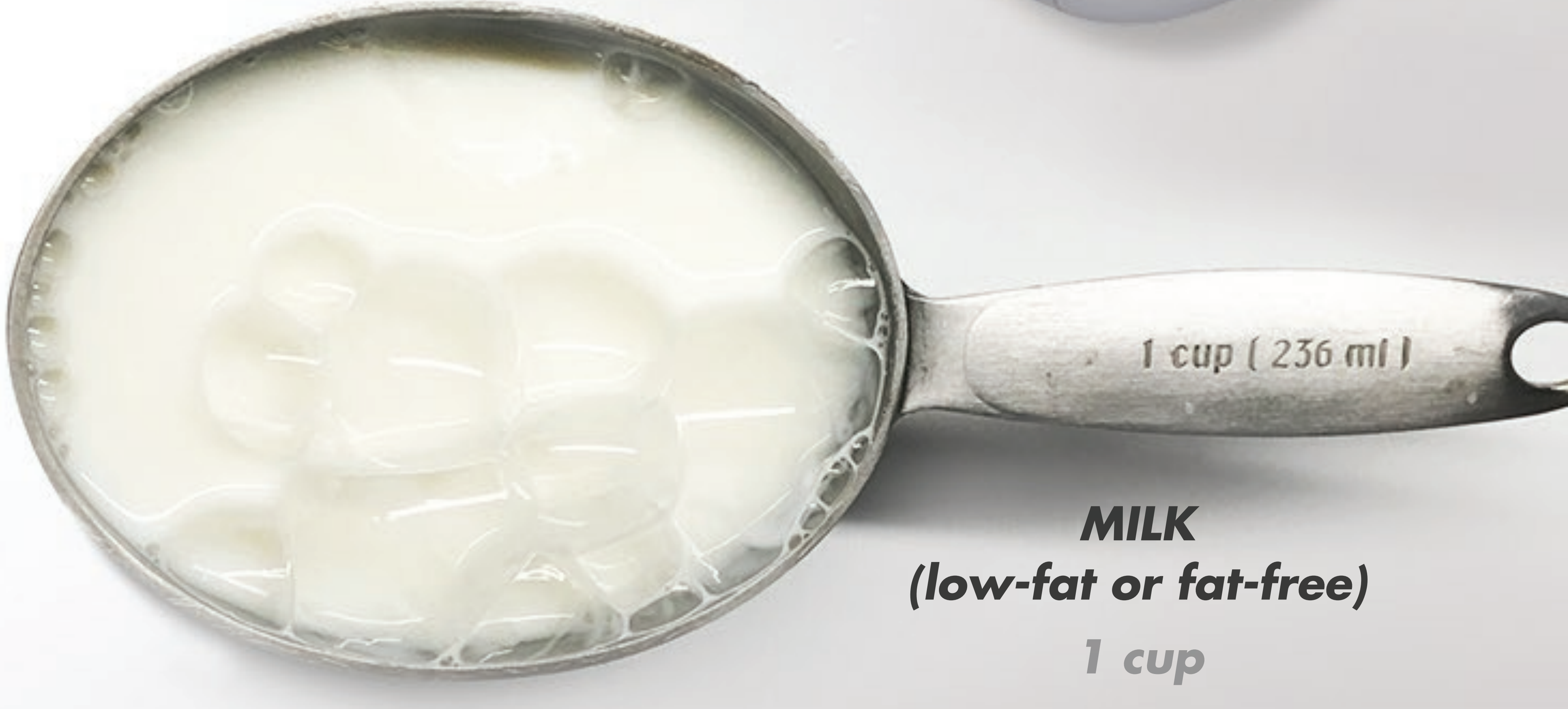

MILK
(low-fat or fat-free)
1 cup

LEARN MORE AT
HEART.ORG/HEALTHYFORGOOD

EAT SMART   ADD COLOR   MOVE MORE   BE WELL

ADDED Sugar IS NOT SO Sweet

American Heart Association®
life is why™

Some sugars are naturally in fruits, vegetables, milk & grains.

Other sugars — the kind added to foods, drinks and condiments during processing — may increase heart disease risk.

A typical 12-ounce can of regular soda has 130 calories and 8 teaspoons of sugar.

Added sugar also sneaks into seemingly "better for you" beverages, such as sports drinks, fruit drinks and flavored milks.

THE AMERICAN HEART ASSOCIATION RECOMMENDS

limiting added sugars to no more than 100 calories a day (6 teaspoons) for most women & no more than 150 calories a day (9 teaspoons) for most men.

ADDED SUGAR SOURCES

Sugar-sweetened beverages are the biggest source of added sugar in the American diet. Other sources are baked items (like cakes, muffins, cookies and pies), ice cream and candy.

FIND IT

Read food labels. Syrup, molasses, cane juice and fruit juice concentrate mean added sugar as well as most ingredients ending with the letters "ose" (like fructose & dextrose).

Enjoy fruit for dessert most days and limit traditional desserts to special occasions.

Cut back on the amount of sugar you add to things you eat or drink often.

Buy 100% juice with no added sugars.

REPLACE IT

Enhance foods with spices — try cinnamon, nutmeg or ginger.

Add fresh or dried fruit to cereal and oatmeal.

Drink sparkling water, unsweetened tea or sugar-free beverages.

Eating and drinking a lot of added sugar is one probable cause of the obesity epidemic in the U.S. It's also linked to increased risks for high blood pressure, high cholesterol, diabetes and inflammation in the body.

Simple Cooking with Heart

FOR MORE TIPS ON HEALTHY EATING, COOKING AND RECIPES: HEART.ORG/SIMPLECOOKING

©2014 American Heart Association. 4/3DS7892

American Heart Association® life is why™ | Healthy For Good™

CUT OUT ADDED SUGARS

Added sugars are sugars added to foods and beverages when they're processed or prepared. Consuming too much may make you sick and may even shorten your life.

WOMEN

MEN

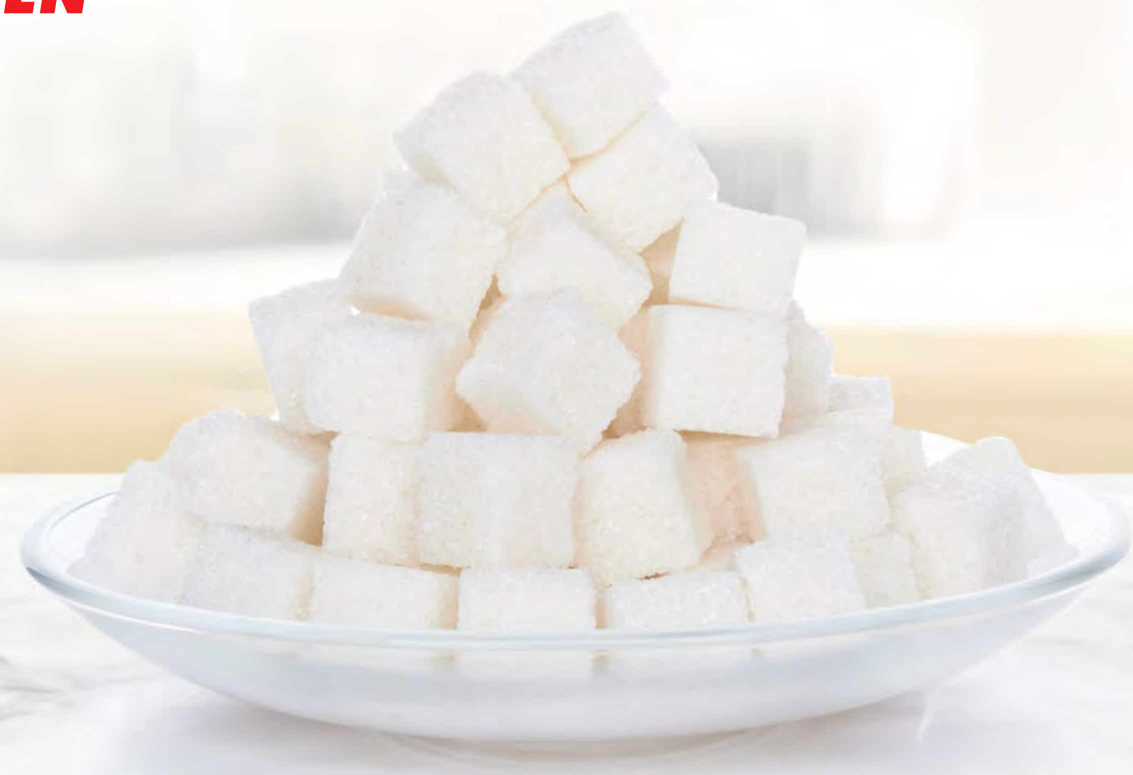

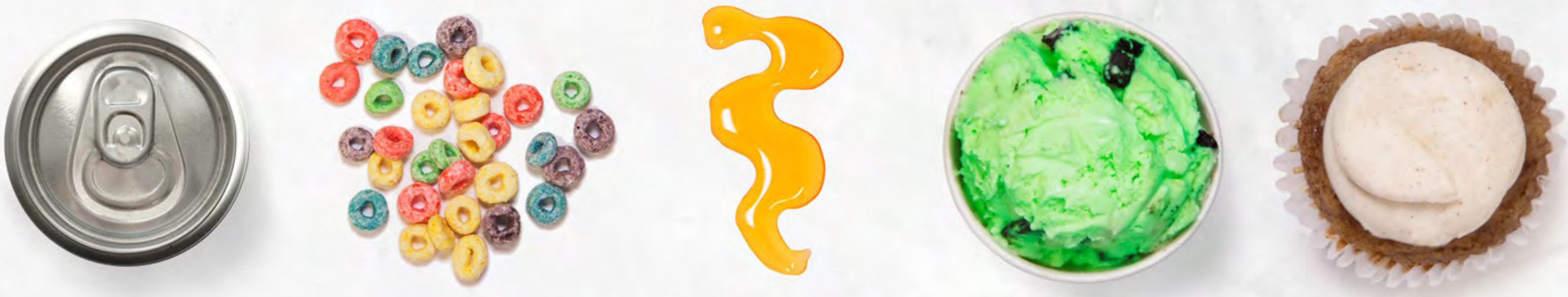

HEART.ORG/HEALTHYFORGOOD

Life is Sweet...
with these Easy Sugar Swaps!

If you're cutting back on added sugars or calories, try these swaps to get the sweet taste you love:

Baking and Cooking

Unsweetened applesauce can substitute for some of the sugar in a recipe. You may need less oil, too—adjust the recipe as needed to get the taste and texture you like. Or try using a no-calorie sweetener suitable for cooking and baking.

Dressings and Sauces

Swap store-bought bottled salad dressings, ketchup, tomato sauce and barbeque sauce — which can have a lot of added sugars — for homemade versions so you can control the amount of sugar added to them.

Soda/Soft Drinks

Swap sugar-sweetened beverages for plain or sparkling water flavored with mint, citrus, cucumber or a splash of 100% fruit juice.

Desserts and Sweets

Instead of indulging in a traditional sugar-based dessert, enjoy the natural sweetness of fruit. Fresh, frozen and canned (in its own juice or water) are all good choices. Try them baked, grilled, stewed or poached.

Snack Mix and Granola

Make your own, without all the added sugars. Combine your favorite nuts and seeds (unsalted or very lightly salted), raisins and dried fruits (unsweetened), rolled oats and whole-grain cereal (non-sugared/non-frosted) — and skip the candy!

Tea and Coffee

Swap sugars (including honey and agave syrup) for a no-calorie sweetener. One packet adds about the same sweetness as two teaspoons of sugar — and typically saves you more than 25 calories.

Get recipes and more tips at
HEART.ORG/RECIPES

The American Heart Association recommends cutting back on added sugars. Using low- and no-calorie sweeteners is one option that may help in an overall healthy diet. Foods and beverages containing low- and no-calorie sweeteners can be included in a healthy eating plan, as long as the calories they save are not added back as a reward or compensation. The FDA has determined that certain low- and no-calorie sweeteners, such as sucralose, are safe.

It's important to eat an overall healthy dietary pattern that **includes** a variety of fruits and vegetables, whole grains, beans and legumes, fish, skinless poultry, nuts and seeds, and fat-free/low-fat dairy products; and **limits** sodium, saturated fat, red meat and added sugars.

Simple Cooking with Heart

FOR MORE TIPS ON HEALTHY EATING, COOKING AND RECIPES: HEART.ORG/SIMPLECOOKING

©2015, American Heart Association. DS9399 7/15

USDA
United States Department of Agriculture

10 tips
Nutrition Education Series

MyPlate MyWins

Based on the Dietary Guidelines for Americans

Make better beverage choices

A healthy eating style includes all foods and beverages. Many beverages contain added sugars and offer little or no nutrients, while others may provide nutrients but too many calories from saturated fat. Here are some tips to help you make better beverage choices.

1 Drink water

Drink water instead of sugary drinks. Non-diet soda, energy or sports drinks, and other sugar-sweetened drinks contain a lot of calories from added sugars and few nutrients.

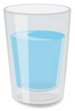

2 How much water is enough?

Let your thirst be your guide. Everyone's needs are different. Most of us get enough water from the foods we eat and the beverages we drink. A healthy body can balance water needs throughout the day. Drink plenty of water if you are very active or live or work in hot conditions.

3 A thrifty option

Water is usually easy on the wallet. You can save money by drinking water from the tap at home or when eating out.

4 Manage your calories

Drink water with and between your meals. Adults and children take in about 400 calories per day as beverages—drinking water can help you manage your calories.

5 Kid-friendly drink zone

Make water, low-fat or fat-free milk, or 100% juice an easy option in your home. Have ready-to-go containers available in the refrigerator. Place them in lunch boxes or backpacks for easy access when kids are away from home. Depending on age, children can drink ½ to 1 cup, and adults can drink up to 1 cup of 100% fruit or vegetable juice* each day.

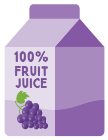

*100% juice is part of the Fruit or Vegetable Group.

6 Don't forget your dairy**

Select low-fat or fat-free milk or fortified soy beverages. They offer key nutrients such as calcium, vitamin D, and potassium. Older children, teens, and adults need 3 cups of milk per day, while children 4 to 8 years old need 2½ cups and children 2 to 3 years old need 2 cups.

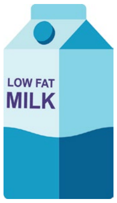

7 Enjoy your beverage

When water just won't do—enjoy the beverage of your choice, but just cut back. Remember to check the serving size and the number of servings in the can, bottle, or container to stay within calorie needs. Select smaller cans, cups, or glasses instead of large or supersized options.

8 Water on the go

Water is always convenient. Fill a clean, reusable water bottle and toss it in your bag or briefcase to quench your thirst throughout the day. Reusable bottles are also easy on the environment.

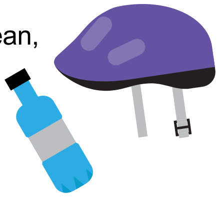

9 Check the facts

Use the Nutrition Facts label to choose beverages at the grocery store. The food label and ingredients list contain information about added sugars, saturated fat, sodium, and calories to help you make better choices.

10 Compare what you drink

Food-A-Pedia, an online feature available at SuperTracker.usda.gov, can help you compare calories, added sugars, and fats in your favorite beverages.

** Milk is a part of the Dairy Group. A cup = 1 cup of milk or yogurt, 1½ ounces of natural cheese, or 2 ounces of processed cheese.

American Heart Association. | Healthy For Good™

SIP SMARTER

Replace sweetened drinks to cut back on added sugars and empty calories.

REPLACE SUGARY BEVERAGES...

- full-calorie soft drinks
- energy/sports drinks
- sweetened "enhanced water" drinks
- sweet tea
- sweetened coffee drinks

WITH BETTER CHOICES!

- The best thing you can drink is water! Try it plain, sparkling or naturally flavored with fruit or herbs.
- Drink coffee and tea without added sugars for a healthier energy boost.
- For adults, diet drinks may help replace high-calorie sodas and other sugary drinks.

THE FACTS MAY SURPRISE YOU.

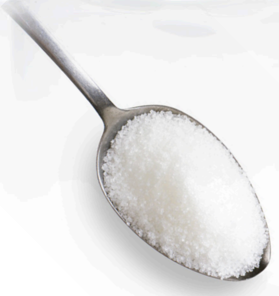

Most Americans consume nearly **20 TEASPOONS** of added sugars **EACH DAY.**

That's more than **TRIPLE** the recommended daily limit for women and **DOUBLE** for men!

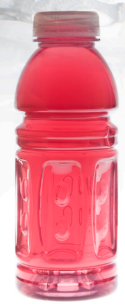

Sugar-sweetened beverages like soda and energy/sports drinks are the **#1 SOURCE OF ADDED SUGARS IN OUR DIET.**

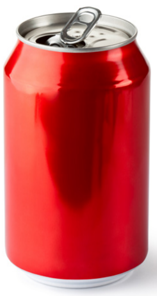

A can (12 FL OZ) of regular soda has about **150 CALORIES AND 10 TEASPOONS** of added sugar.

TRY THESE TIPS TO QUENCH YOUR THIRST WITH LESS ADDED SUGARS

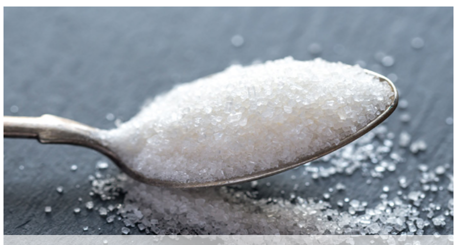

START CUTTING BACK.

Take steps to reduce or replace sugary drinks in your diet:

REPLACE most of your drinks with water.

REDUCE the amount of sugar in your coffee or tea gradually until your taste adjusts to less sweetness.

ADD plain or sparkling water to drinks to keep some of the flavor with less added sugars per servings.

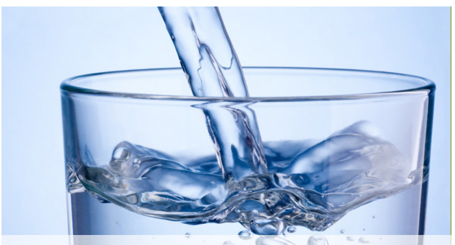

CHOOSE WATER.

Make water the easy, more appealing go-to choice:

CARRY a refillable water bottle.

ADD a splash of 100% fruit juice or slices of citrus, berries and even cucumbers for a boost of flavor.

TRY seltzer, club soda or sparkling water if you crave the fizz.

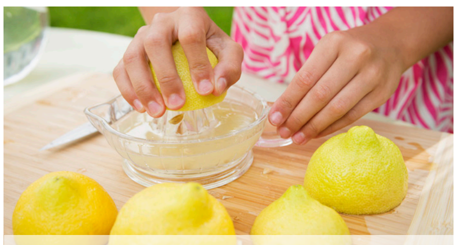

MAKE IT AT HOME.

Family favorites like hot chocolate, lemonade, smoothies, fruit punch, chocolate milk and coffee drinks easily can be made at home with less added sugars.

START WITH UNSWEETENED beverages, then flavor to taste with additions like fruit, low-fat or fat-free milk, and herbs and spices.

Get great recipes for beverages and more at **HEART.ORG/RECIPES**.

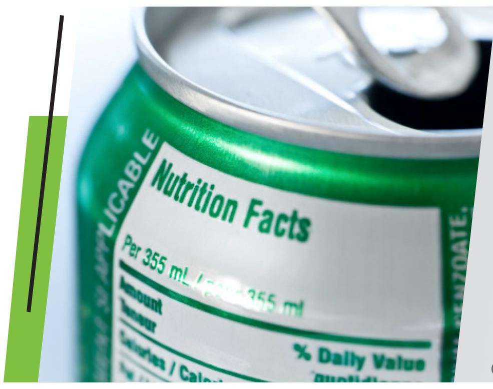

READ THE LABEL, AND CHOOSE WISELY.

Some drinks that appear to be healthy may be high in calories and added sugars. Check servings per container and ingredients list.

Added sugars go by many names, including sucrose, glucose, maltose, dextrose, high fructose corn syrup, cane syrup, concentrated fruit juice, agave nectar and honey.

EAT SMART ADD COLOR MOVE MORE BE WELL

FOR MORE TIPS ON HEALTHY EATING, COOKING AND RECIPES: HEART.ORG/EATSMART

©2018, American Heart Association 7/18DS13619

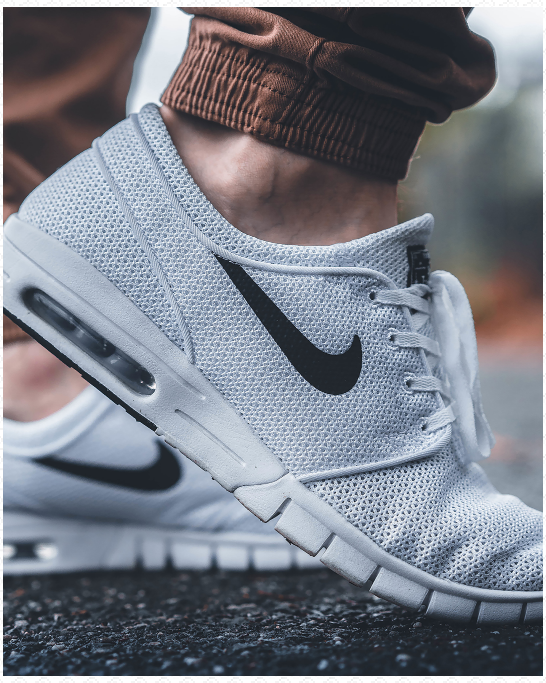

2015-2020

Physical Activity Guidelines for Americans

In addition to consuming a healthy eating pattern, regular physical activity is one of the most important things Americans can do to improve their health.

18 to 64 years

Adults should do at least 150 minutes a week of moderate-intensity, or 75 minutes a week of vigorous-intensity aerobic physical activity.

Aerobic activity should be performed in episodes of at least 10 minutes, and preferably, it should be spread throughout the week.

Adults should also include muscle-strengthening activities that involve all major muscle groups on 2 or more days a week.

For additional and more extensive health benefits...

adults should increase their aerobic physical activity to 300 minutes a week of moderate-intensity, or 150 minutes a week of vigorous-intensity aerobic physical activity.

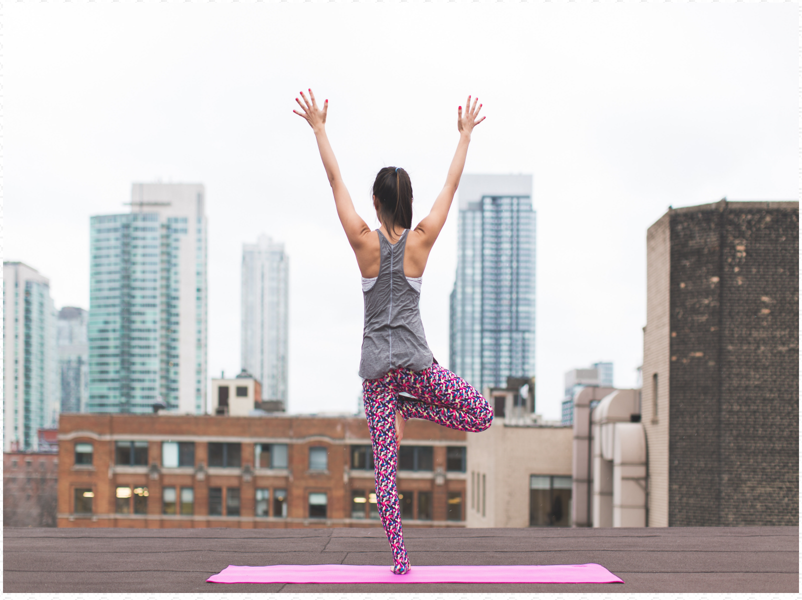

Health Tip!

All adults should avoid inactivity. Some physical activity is better than none. Any amount of physical activity helps you gain some health benefits.

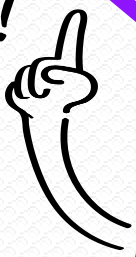

65 years and older

Older adults should follow the adult guidelines. When older adults cannot meet the adult guidelines, they should be as physically active as their abilities and conditions will allow.

Older adults with chronic conditions should understand whether and how their conditions affect their ability to do regular physical activity safe.

SAFETY FIRST!

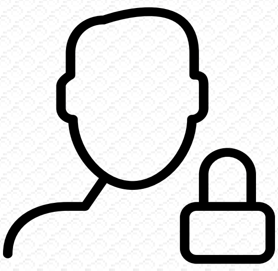

We are MPN

For a description of moderate-intensity and vigorous-intensity physical activity, as well as muscle-strengthening activity
visit: https://health.gov/dietaryguidelines/2015/guidelines/appendix-1/
